# Activation and Catalytic Degradation of SF_6_ and PhSF_5_ at a Bismuth Center

**DOI:** 10.1021/jacs.4c07044

**Published:** 2024-09-03

**Authors:** Vanessa A. Béland, Nils Nöthling, Markus Leutzsch, Josep Cornella

**Affiliations:** Max-Planck-Institut für Kohlenforschung, Kaiser-Wilhelm-Platz 1, Mülheim an der Ruhr 45470, Germany

## Abstract

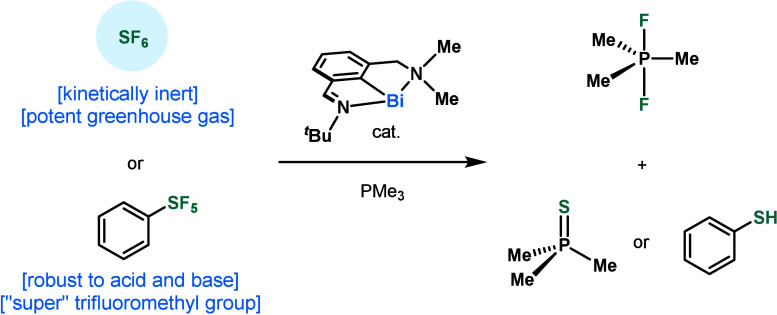

In this work, we report the catalytic degradation of SF_6_ and PhSF_5_ using *N*,*C*,*N* pincer bismuthinidene complexes (**1** and **5**). Exposure of SF_6_ and PhSF_5_ to **1** results in the reduction of the S(VI) substrates and concomitant formation of Bi(III) and Bi(II) compounds, which were isolated and characterized. The oxidized bismuth-based products were demonstrated to undergo reduction with PMe_3_, recovering the starting complex **1**. Having established a synthetic redox cycle, the catalytic degradation of SF_6_ and PhSF_5_ was developed through ligand optimization to **5**, leading to a 528 TON for SF_6_ and the first reported TON for PhSF_5_ (3.2).

The chemical inertness and high dielectric constant of sulfur hexafluoride (SF_6_) led to its industrial production starting in 1953 for use in a range of applications.^1^ Yet, its cherished inertness comes at a cost: SF_6_ is a potent greenhouse gas making it a major risk factor for the global climate. Emissions of SF_6_ have been steadily increasing since 1978 and were recorded to be 9,000 ± 400 tons per year in 2018.^2^ Aside from the negligible amount of naturally occurring SF_6_ (ca. 0.1 pptv), its presence in the atmosphere (ca. 10 pptv) is anthropogenic.^3^ Although it is currently found in low concentrations relative to other greenhouse gases, it is estimated to have 23,500 times more warming potential than CO_2_, due to its lifetime of 580–3,200 years.^4^ In response, multidisciplinary solutions to the SF_6_ problem have emerged,^5−8^ building tangible precedence from which alternative processes for its catalytic degradation can be developed.

Although SF_6_ succumbs to decomposition under harsh conditions,^9^ the key to activating the kinetically stabilized molecule under mild conditions hinges on accessing the unstable SF_6_^•–^, followed by fragmentation.^1,10^ Indeed, mild activation of SF_6_ has been reported with a plethora of transition metal compounds, including Ti, V, Cr, Fe, Ni, Zr, Rh, Ir or Pt (Figure 1A).^11−16^ More recently, main group complexes have also been shown to act as stoichiometric reducing agents and activate SF_6_, leading to diverse compounds.^17−20^ In particular, electron-rich Al, N, and P-based compounds as well as photoexcited complexes have proven to be promising candidates (Figure 1B).^15,20,21^ In a similar manner, the monodefluorinated analog PhSF_5_ can also be degraded with transition metal complexes based on Rh and Ni.^18,19^ Despite the great advances in the area, a catalytic degradation protocol of SF_6_ and PhSF_5_ with a main group catalyst still remains elusive.

**Figure 1:**
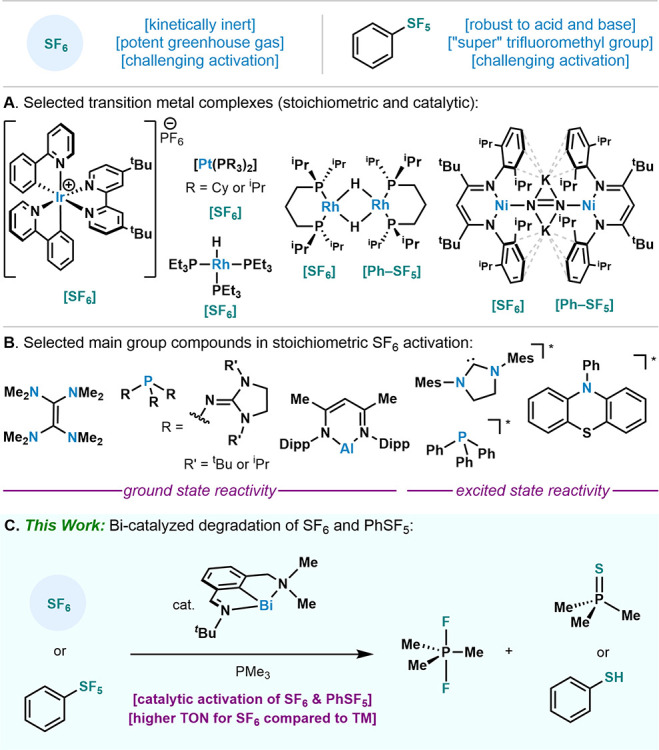
SF_6_ and PhSF_5_ degrader toolbox. * denotes excited state reactivity.

Based on precedents in group 15 and previous work on the redox properties of low-valent *N*,*C*,*N*-bismuthnidenes,^22−26^ we envisioned that **1** would be a good candidate to activate SF_6_. In this work, we report a unique low-valent bismuth redox cycle capable of the degradation of SF_6_ and PhSF_5_ (Figure 1C). This uncommon main group-based protocol proceeds through the intermediacy of Bi(III) and Bi(II) complexes, which could be isolated and fully characterized. We demonstrate how these oxidation products can be successfully reduced back to **1** by the action of a simple phosphine, thus regenerating the propagating species. Further, with the use of a more electron-rich bismuthinidene supported by an asymmetric imine-amine pincer ligand (**5**), we show catalytic degradation of SF_6_ and PhSF_5_.

Based on our previous observations on the stoichiometric activation of aromatic C–F bonds,^23^ we reacted **1** with SF_6_ in the presence of LiOTf as an *in situ* fluoride scavenger in MeCN at 22 °C (Scheme 1A). A gradual color change from dark teal to yellow was observed over the course of 3 days. Analysis of the reaction mixture by ^1^H NMR spectroscopy revealed the formation of two new bismuth species in a 2:1 ratio, attributed to **2** and **3** (see Figure S2). These compounds could be separated by selective crystallization and isolated in 67% and 55% yield, respectively. The compounds were also structurally characterized to reveal two dicationic bismuth species, which provide insight on the redox chemistry between Bi and SF_6_. On one hand, Bi(II) dimer (**2**) is the result of a 1-electron oxidation of **1** by SF_6_, which consumes the first four F atoms. On the other hand, Bi(III) sulfide bridged dimer **3** is the result of a formal 2-electron oxidation that consumes the S and final two F atoms. Organobismuth(II) dimers have been reported to undergo elemental chalcogen atom (O, S, Se, Te) insertion into the Bi–Bi bond.^27^ In order to test this possibility for the formation of **3**, **2** was reacted with elemental sulfur and formation of **3** could be observed by NMR spectroscopy (72% NMR yield, Scheme 1A). Compound **2** can also be prepared directly by single electron oxidation using ferrocenium triflate (62% isolated yield, see SI),^28^ further confirming the 1-electron process.

**Scheme 1:**
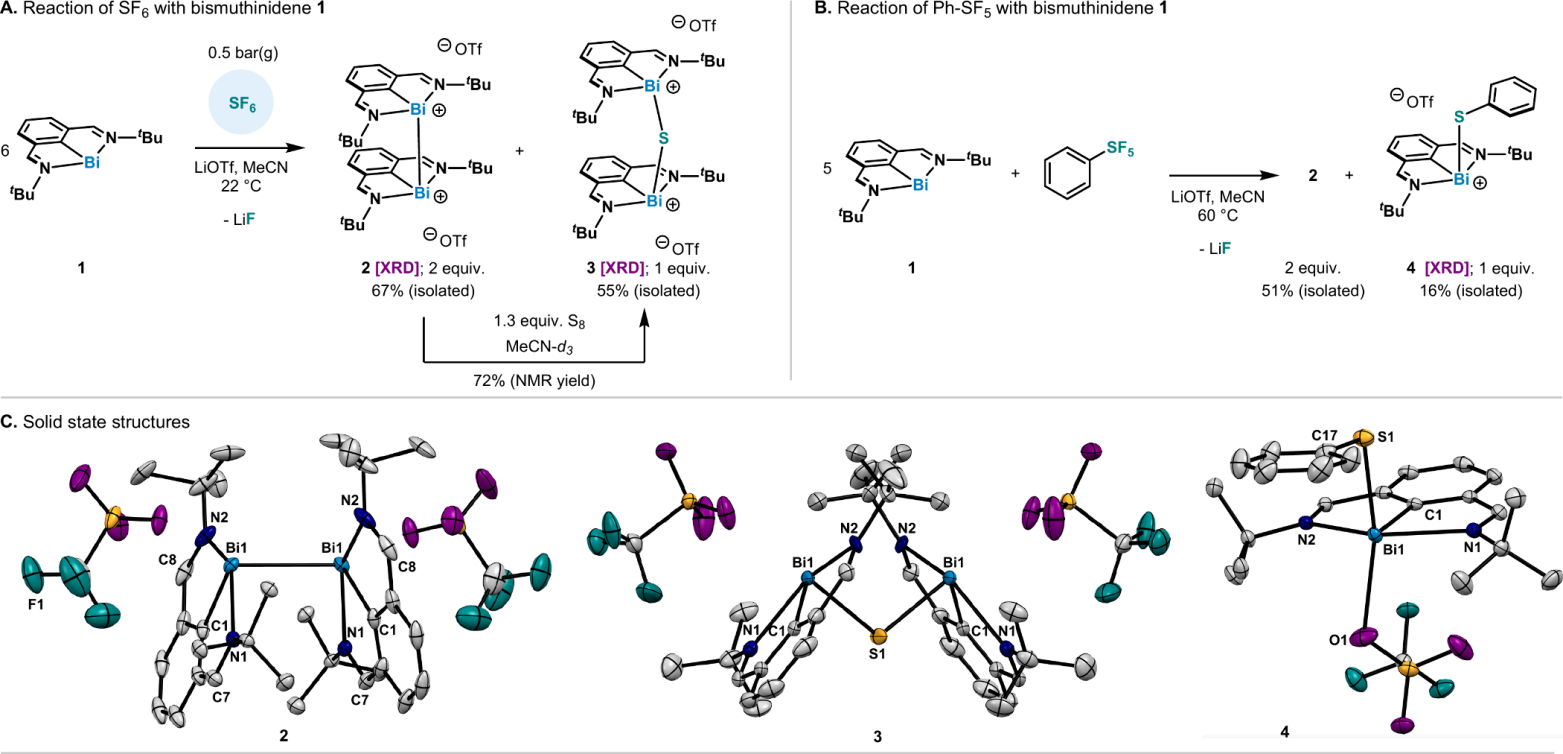
Activation of SF_6_ and PhSF_5_ with **1** (A) Compound **1** (6.0 equiv) was stirred with LiOTf (6.0 equiv) in acetonitrile under 0.5 bar(g) SF_6_ at 22 °C for 3 days; (B) Compound **1** (1.0 equiv) was heated to 60 °C with LiOTf (1.0 equiv) and PhSF_5_ (2.5 equiv) in acetonitrile for 3 days; (C) Solid state structures of **2**, **3** and **4**, visualized with 50% probability ellipsoids. For the sake of clarity, H atoms, disorder and solute molecules have been omitted. Relevant XRD distances and angles: **2**: C(1)–Bi(1) 2.1879(17) Å, N(1)–Bi(1) 2.4698(14) Å, N(2)–Bi(1) 2.5438(16) Å, Bi(1)–Bi(1) 3.09439(19) Å, C(1)–Bi(1)–N(1) 71.82(6)°, C(1)–Bi(1)–N(2) 70.87(7)°; **3**: C(1)–Bi(1) 2.200(3) Å, N(1)–Bi(1) 2.453(3) Å, N(2)–Bi(1) 2.572(4) Å, Bi(1)–S(1) 2.5497(8) Å, C(1)–Bi(1)–N(1) 72.04(11)°, C(1)–Bi(1)–N(2) 70.22(12)°, Bi(1)–S(1)–Bi(1) 107.54(5)°; **4**: C(1)–Bi(1) 2.194(3) Å, N(1)–Bi(1) 2.491(2) Å, N(2)–Bi(1) 2.488(2) Å, Bi(1)–S(1) 2.5702(8) Å, S(1)–C(17) 1.780(3) Å, C(1)–Bi(1)–N(1) 71.37(9)°, C(1)–Bi(1)–N(2) 71.28(9)°, Bi(1)–S(1)–C(17) 98.22(10)°.

Analogous to SF_6_, aryl sulfur pentafluorides (ArSF_5_) have been shown to degrade only under strong hydrolytic conditions,^29^ thus finding applications as robust lipophilic groups in pharmaceuticals and materials.^30^ When a mixture of PhSF_5_, LiOTf and **1** was heated at 60 °C, quantitative conversion of **1** was observed, leading to the formation of **2** and a new bismuth(III) thiophenolate species (**4**), which could be crystallized and isolated from the reaction mixture in 16% yield (Scheme 1B). These findings demonstrate a rare example of PhSF_5_ activation,^13,14^ seemingly through the same 1- and 2-electron processes by which Bi activates SF_6_. To further confirm the nature of **4**, the same compound was prepared and isolated via the reaction of benzenesulfenyl triflate with **1** (91% isolated yield, see SI).

The solid-state structures of **2**, **3** and **4** were obtained by single crystal XRD (Scheme 1C). Compound **2** is dimeric with the adjacent ligand planes staggered and slightly twisted. Compound **2** is a 1,2-dication,^31^ which are precedented structures for heavy main group elements in low oxidation states.^32^ The Bi–Bi bond in **2** (3.09439(19) Å) is on the long end compared to neutral Bi(II) complexes (*cf.* 2.796–3.209 Å, see SI).^33^ Compound **3** exhibits two pincer-ligand-bearing bismuth centers bridged by a sulfur atom. The ligand planes are staggered and nearly perpendicular. Compound **4** displays a distorted square pyramidal bismuth center chelated by the pincer ligand, and coordinated by thiophenolate and triflate ligands *trans* to each other. The Ph group is in an *anti*-configuration relative to that of the pincer ligand.

In the case of **3** and **4**, sulfur S(1) is bent, attributed to the lone-pair-bearing sulfide and thiophenolate ligands in the complexes: a structural beacon of the multiple electron reduction that occurred from the S(VI) starting materials.

To explore the possibility of reducing the oxidative addition products, we selected trimethyl phosphine (PMe_3_), due to its ability to scavenge both S and F and for its inertness toward the ligand imines.^12,16,18−20^

The reduction studies were performed in the presence of excess wet [NMe_4_][F] to mirror the fluoride activity in an envisioned catalytic manifold. It was found that in the presence of stoichiometric PMe_3_ and excess [NMe_4_][F], **2**, **3** and **4** could be reduced to **1** in >95%, 67% and 85% NMR yield, respectively (Scheme 2A). In all cases, phosphine oxide (OPMe_3_) (δ_P_ = 36.9 ppm) is observed as a byproduct instead of (OTf)_2_PMe_3_, due to *in situ* hydrolysis. In the case of **3**, SPMe_3_ is also observed as a byproduct (δ_P_ = 30.9 ppm) and exclusively accounts for the fate of the sulfur atom. The NMR yields were determined to be 61% for OPMe_3_ and >95% for SPMe_3_. These yields manifest that the reduction of the triflate portion could account for the low recovery of **1**. In the case of **4**, OPMe_3_ and ammonium thiophenolate ([NMe_4_][SPh]) are observed as byproducts (see Figures S6–S8), indicating that F^–^ likely displaces PhS^–^ from bismuth.

**Scheme 2:**
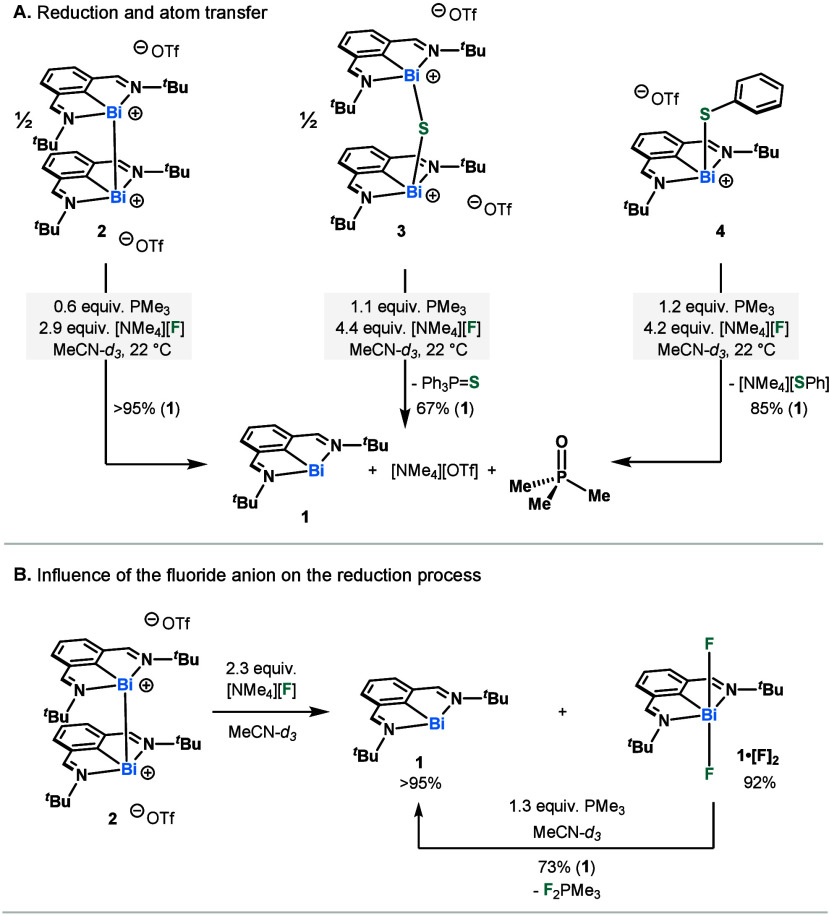
(A) Reduction of **2**, **3** and **4** Using PMe_3_ as Electron Donor and Atom Acceptor; (B) Disproportionation of **2**

Interestingly, we found that the presence of [NMe_4_][F] results in the rapid and quantitative disproportionation of **2** into **1** and **1·[F]**_**2**_. Moreover, PMe_3_ can also reduce **1·[F]**_**2**_ to **1** in 73% NMR yield, thus highlighting that the reduction protocol is agnostic to such disproportionation events (Scheme 2B).

Having validated the stoichiometric redox cycling, the degradation of SF_6_ was attempted using catalytic amounts of **1**. With PMe_3_ as a reducing agent, the formation of F_2_PMe_3_ and phosphine sulfide (SPMe_3_) could be observed with 2.0 mol % **1**. The reaction was slow, with only 32% yield of SPMe_3_ and 4.0 TON SF_6_ formed after 1 month at 25 °C. However, **1** was observed to be the resting state and the concentration remained constant throughout the entire NMR monitoring time (1 month), indicating the robustness of the catalyst to the reaction conditions (Figures S16–S17). Switching to a bismuthinidene supported by an asymmetric pincer ligand (**5**)—where one of the supporting imine arms is replaced with a stronger σ-donating amine—results in a more electron-rich Bi(I) complex (*E*_1/2_: **5** = −1.01 V, cf. **1** = −0.85 V).^24,25^ With 2.1 mol % **5** and using PMe_3_ as reducing agent, 70% SPMe_3_ and 7.9 TON could be achieved in 11 days under 1 bar(g) of SF_6_ (Scheme 3A). All SF_6_ was consumed from solution and PMe_3_ remained in excess by the end of the NMR monitoring time. When the reaction mixture was heated to 60 °C, a 97% NMR yield of SPMe_3_ was obtained after 3 days, corresponding to SF_6_ TON of 528. This TON is almost an order of magnitude higher than the one reported by Zámostná and Braun with a rhodium catalyst (86 TON).^16^

**Scheme 3:**
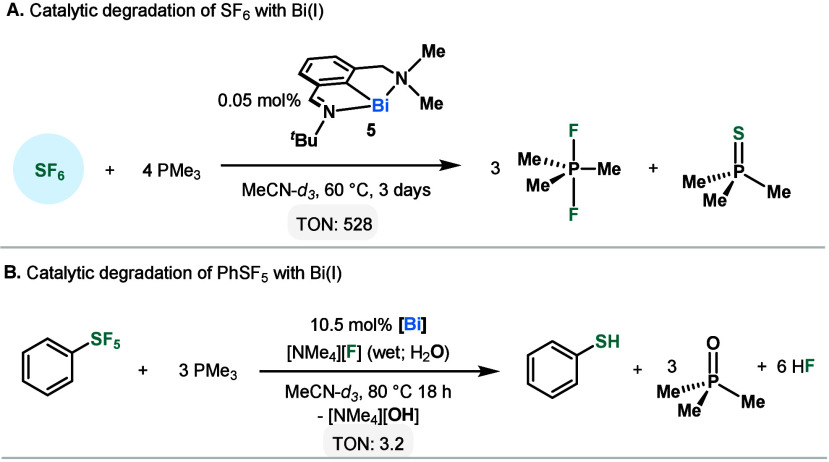
Catalytic Degradation of SF_6_ and PhSF_5_

Whereas the catalytic degradation of SF_6_ has a good driving force with the formation of F_2_PMe_3_ and SPMe_3_, removal of thiophenolate from Bi requires the use of an electrolyte. Analogous to the stoichiometric reduction of **4**, the use of wet [NMe_4_][F] was found to be necessary for PhSF_5_ to be catalytically degraded using 10.5 mol % **5** and PMe_3_ as reducing agent, achieving 3.2 TON at 80 °C (Scheme 3B). To the best of our knowledge, this represents the first example of catalytic degradation of PhSF_5_ and provides a blue print for further developments.

Our proposed catalytic cycle is initiated by single electron transfer (SET) from bismuthinidene **I** to SF_6_, to generate a Bi(II) fluoride species (**II**) and sulfur(IV) tetrafluoride (SF_4_) (Scheme 4). Species **II** rapidly disproportionates to **I** and difluoro Bi(III) species **IV**. Compound **IV** is then reduced by PMe_3_ to **I** via reductive defluorination. The control experiment (Table S4, entry 3) as well as precedence from Dielmann *et al*.^20^ demonstrate that PMe_3_ cannot activate SF_6_ on its own, solidifying the necessity of **5** in this initial step. Although SF_4_ and SF_2_ have not been directly observed in the reaction mixture, we speculate their short-lived presence based on the stoichiometric studies. These highly reactive fluorinating agents have been shown to directly scavenge phosphines,^34^ and this is likely competitive with subsequent reactivity with **I**. Nonetheless, our stoichiometric studies have shown the possibility of propagating through a bridging sulfide Bi(III) species (**III**). The latter can undergo reductive atom transfer by PMe_3_, affording SPMe_3_. In the case of PhSF_5_, the sulfur turnover is dependent on anion metathesis of fluoride for thiophenolate at bismuth. This would result in **IV**, which undergoes reductive dehalogenation by PMe_3_, followed by the hydrolysis of both PhS^–^ and F_2_PMe_3_ to give thiophenol and OPMe_3_ (Scheme S1).

**Scheme 4:**
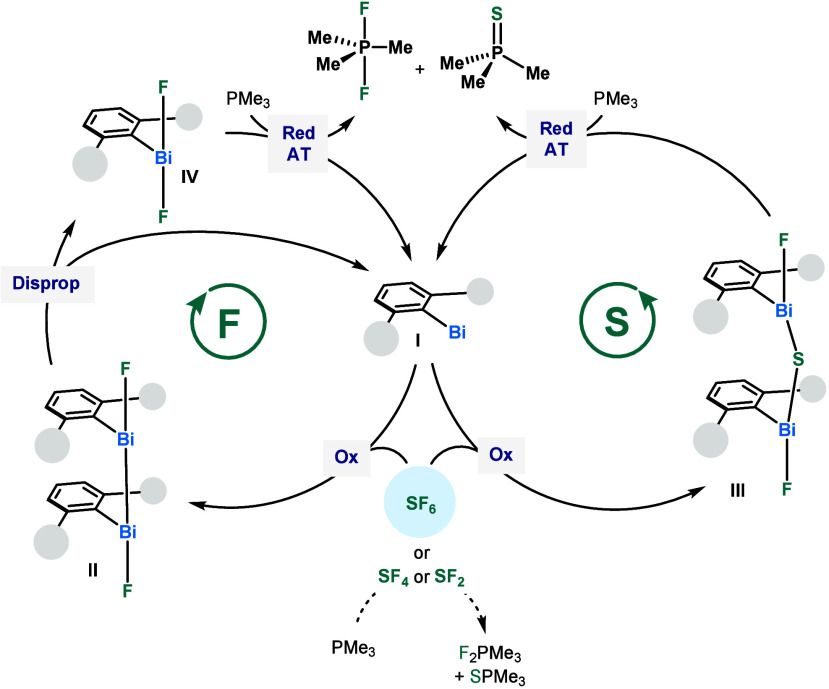
Postulated Mechanism for the Catalytic Degradation of SF_6_ Red: reduction; AT: Atom transfer; Ox: oxidation; Disprop: disproportionation.

In this work, we have reported the stoichiometric degradation of SF_6_ and PhSF_5_ using a Bi(I) complex supported by a *N*,*C*,*N* pincer ligand (**1**). We demonstrate that Bi(I) is capable of reducing SF_6_ via SET, leading to mixtures of Bi(II) and Bi(III) intermediates, which could be isolated and fully characterized. The oxidation products were reduced back to catalytically active **1** by using PMe_3_. Finally, with all organometallic steps validated in a stoichiometric fashion, the catalytic degradation of SF_6_ and PhSF_5_ was developed with low-valent Bi(I) complex **5**. The catalyst scores a remarkable 528 TON for SF_6_ and provides the first reported catalytic destruction of PhSF_5_ (3.2 TON).

## Supporting Information Available

Experimental and characterization details (PDF)

## Abbreviations

AT: atom transfer
bar(a): bar absolute pressure
bar(g): bar gauge pressure
Disprop: disproportionation
Equiv.: equivalents
NMR: nuclear magnetic resonance
NOESY: Nuclear Overhauser Effect Spectroscopy
Ox: oxidation
Red: reduction
SET: single electron transfer
^t^Bu: tertiary butyl
TON: turnover number
XPS: X-ray photoelectron spectroscopy
XRD: X-ray diffraction.
