# Supplementary material for: Activation and Catalytic Degradation of SF6 and PhSF5 at a Bismuth Center
Source: J Am Chem Soc. 2024 Sep 3;146(37):25409–15. doi: 10.1021/jacs.4c07044 (PMC11421020; doi:10.1021/jacs.4c07044)
Supplement: Supplementary file 1 — ja4c07044_si_001.pdf [file ja4c07044_si_001.pdf]

Supporting Information

Activation and Catalytic Degradation of SF_6 and $PhSF_5$ at a Bismuth Center

Vanessa A. Béland, Nils Nöthling, Markus Leutzsch and Josep Cornella*

Max Planck Institut für Kohlenforschung, Kaiser-Wilhelm-Platz 1, Mülheim an der Ruhr, 45470, Germany

cornella@kofo.mpg.de

Contents

1. General Experimental Notes ... 3
2. Stoichiometric Oxidations at Bi ... 5
2.1 Preparation of **2** and **3**: ... 5
2.2 Alternative Synthesis of **2**. ... 7
2.3 Characterization Data of bis-[N,N-di-*tert.*-butyl-isophthalaldimine-2-yl-bismuthenium] bistriflate (**2**)... 7
2.4 Alternative Synthesis of **3**. ... 8
2.5 Characterization Data for bis-[N,N-di-*tert.*-butyl-isophthalaldimine-2-yl-bismuthenium]-μ_2-monosulfide bistriflate (**3**) ... 9
2.6 Preparation of **2** and **4**. ... 10
2.7 Alternative Synthesis of **4**. ... 13
2.8 Characterization Data for [N,N-di-*tert.*-butyl-isophthalaldimine-2-yl-bismuthenium]-thiophenolate triflate (**4**) ... 13
3. Stoichiometric Reductions at Bi ... 14
3.1 Stoichiometric Reduction of **2**... 14
3.2 Stoichiometric Reduction of **3**... 15
3.3 Stoichiometric Reduction of **4**... 16
3.4 Stoichiometric Reduction of **1•[F$_2$]**... 22
4. Disproportionation... 23
4.1 Disproportionation of **2** using [NMe_4][F] ... 23
5. Cyclic Voltammetry ... 24
6. Catalytic Reaction Details ... 26
6.1 SF_6 Reactions ... 26
6.1.1 Notes on catalyst poisoning with inadequate reducing agents ... 27

6.1.2 One Month Reaction Monitoring with PMe_3 as Reducing Agent ... 32
6.1.3 60 °C Reaction Monitoring ... 39
6.1.4 Solvent Optimization ... 43
6.1.5 TON Optimization ... 43
6.1.6 SF_6 TON Calculation ... 44
6.2 $PhSF_5$ Reactions ... 48
6.2.1 Postulated Catalytic Cycle for $PhSF_5$ degradation ... 48
6.2.2 $PhSF_5$ TON Calculation ... 49
6.3 Control Experiments ... 50
7. X-Ray and TEM ... 51
7.1 Single crystal structure analysis of **2** MeCN solvate ... 51
7.2 Single crystal structure analysis of **3** MeCN solvate ... 59
7.3 Single crystal structure analysis of **4** pentane solvate ... 65
7.4 Cambridge Structural Database (CSD) Searches ... 72
7.5 TEM EDX ... 79
7.5.1 TEM EDX analysis of compound **2** ... 79
7.5.2 TEM EDX analysis of Compound **3** ... 81
8. NMR Spectra ... 83
9. Mass Spectra ... 92
10. References ... 94

1. General Experimental Notes

Instrumentation: Manipulations were conducted using standard Schlenk line techniques or in a glove box under an atmosphere of dry Ar gas unless otherwise noted. ^{1}H, ^{13}C, ^{19}F and ^{31}P NMR spectra were recorded with Bruker Avance III HD nanobay 300 MHz, Bruker Avance III HD 400 MHz, Bruker Avance III 500 MHz, or Bruker Avance NEO 600 MHz NMR spectrometers, and processed using MestReNova. ^{1}H NMR chemical shifts are given in ppm with respect to the residual solvent peak (C_6D_6, δ 7.16 ppm; THF-d_8, δ 1.73 ppm; CD_3CN, δ 1.94 ppm). $^{13}C\{1H\}$ NMR chemical shifts are given in ppm with respect to solvent peak CD_3CN, δ 118.26 ppm). $^{19}F\{^{1}H\}$ NMR chemical shifts are given in ppm relative to $CFCl_3$. $^{31}P\{^{1}H\}$ NMR chemical shifts are given in ppm relative to phosphoric acid. Multiplicities are described as s = singlet, br s = broad singlet, d = doublet, br d = broad doublet, t = triplet, q = quartet, dd = doublet of doublets, m = multiplet. Coupling constants are reported as J-values in Hz. High-resolution mass spectra were obtained using Bruker APEX III FT-MS with ESI ionization source or Finnigan MAT 95 with EI ionization source. Elemental analysis (C, H, N, S) was performed by the Microanalytical Laboratory Kolbe. Electrochemical experiments were conducted using Gamry Interface 1010E potentiostat.

Reagents: Acetonitrile (MeCN and CD_3CN), deuterated benzene (C_6D_6), diethyl ether (Et_2O), toluene (MePh) and tetrahydrofuran (THF and THF-*d_8*) were dried over calcium hydride, distilled and stored under argon over sieves (3 Å for MeCN and CD_3CN and 4 Å for everything else). Bismuth complexes **1•[F_2**], **1**, and **5•[Cl_2]** were prepared following literature procedures.[1-3] Catalyst **5** was synthesized by reductive dechlorination of **5Cl_2** via K-selectride, as reported in the literature.[4] Lithium trifluoromethanesulfonate (LiOTf) was purchased from Aldrich and dried under vacuum at 300 °C overnight before use. Trimethylphosphine (PMe_3, STREM) and triethylsilane ($HSiEt_3$, Aldrich) were stored in Teflon-sealed Schlenk flasks and used as received. Tetramethyl ammonium fluoride ([NMe_4][F], Aldrich) was used as received. Phenylsulfur penatfluoride ($PhSF_5$) was purchased from TCI and degassed before use. Thallium triflate (TlOTf) was used as received from STREM. CAUTION: TlOTf is highly toxic and should be handled accordingly. Ferrocenium triflate ([Fc][OTf]) was prepared according to the literature procedure.[5] Benzene sulfenyl chloride (PhSCl) was prepared following a literature procedure,[6] then further purified by filtering the reaction mixture, dichloromethane was removed *in vacuo*, the product distilled, then Et_2O (3× 5mL) was added and removed *in vacuo* to azeotrope any residual dichloromethane to give neat PhSCl as a transparent red liquid.

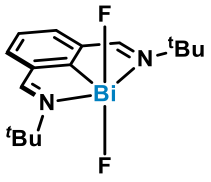

1•[F]$_2$

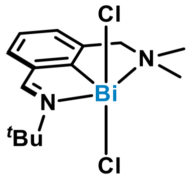

5•[Cl]$_2$

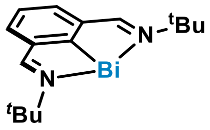

1

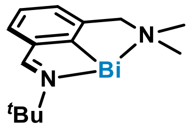

5

A cylinder of SF_6 was purchased from Air Liquide (99.999%), equipped with a regulator and connected to the Schlenk line using rubber tubing equipped with a drying column containing calcium carbonate and anhydrous cobalt chloride indicator. The gas was introduced to reaction mixtures via a three-way key, which could be used to cycle the atmosphere from both the flask line and the SF_6 line in order to ensure an inert atmosphere (and to avoid degassing). This key can also be used to introduce SF_6 to the reaction mixture.

Figure S1: Schlenk line equipped with three-way key for access to vacuum and two gas lines.

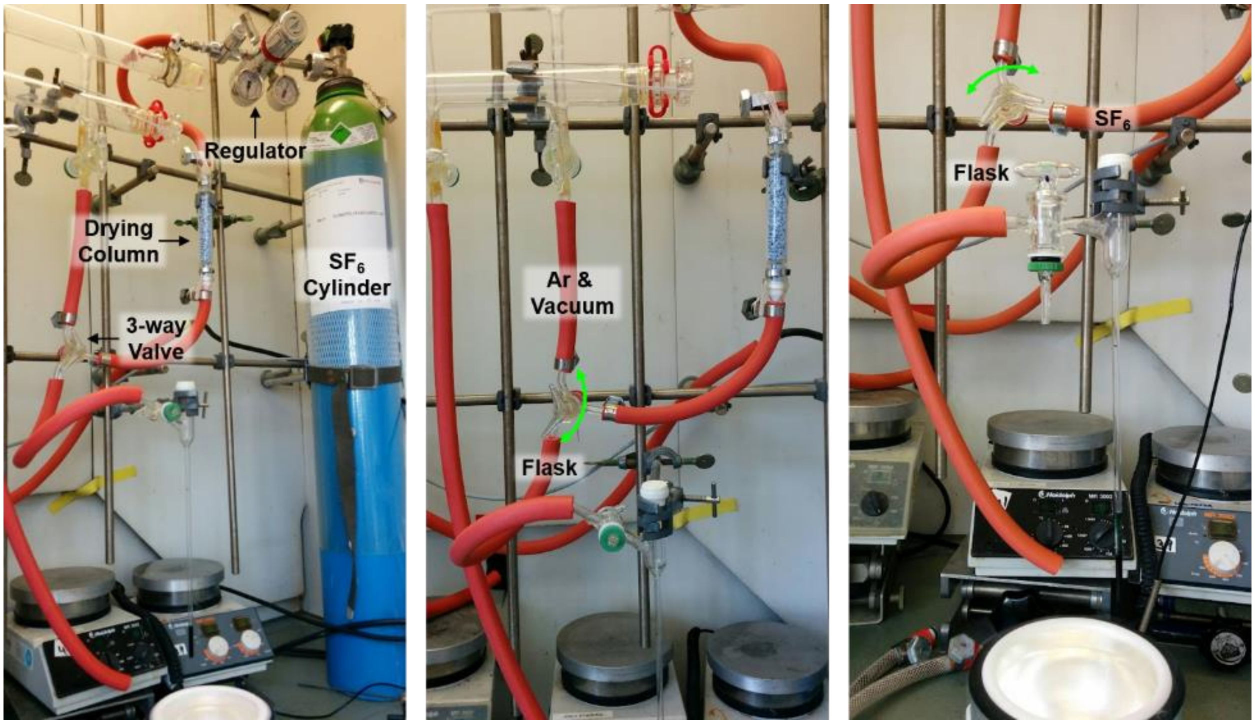

2. Stoichiometric Oxidations at Bi

Initial room temperature reactions of **1** with SF_6 resulted in consumption of **1** and the formation of insoluble materials that could not be characterized structurally or spectroscopically. Inspection of the heteronuclear NMR spectra of the reaction mixture revealed the formation of BF_3•MeCN adduct (δ_F = –146.1 (q) ppm, δ_B = 5.0 (q) ppm, $^1J_{FB}$ = 65 Hz), indicating fluorination of the borosilicate glass from the reaction vessel.

2.1 Preparation of **2** and **3**:

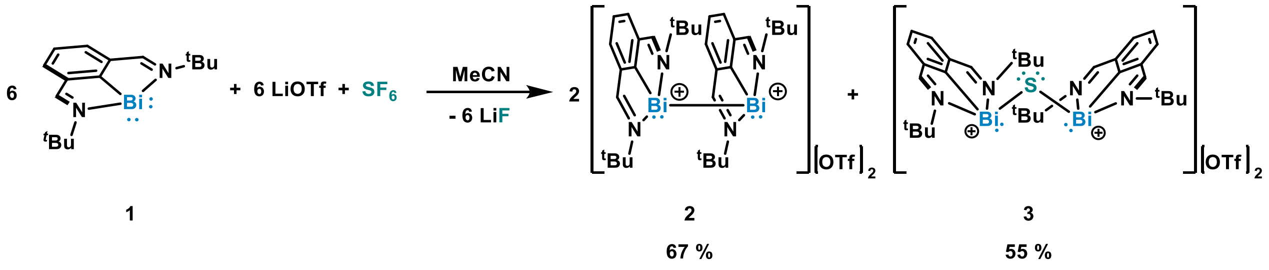

Lithium trifluoromethanesulfonate (36 mg, 0.23 mmol, 6.0 equiv.) and **1** (104 mg, 0.230 mmol, 6.0 equiv.) were dissolved in MeCN (6 mL) and the solution was loaded into a pressure Schlenk flask. The dark teal solution was degassed using three cycles of freeze pump thaw to remove the argon atmosphere, then the flask was pressurized with 0.5 bar(g) of SF_6. The solution was stirred for three days at 22 °C and showed a gradual colour change to dark yellow-brown and a small amount of black precipitate. A ^{1}H NMR spectrum of an aliquot of the reaction mixture revealed a 2:1 ratio of **2** and **3**. The reaction mixture was filtered over Celite, then layered with Et_2O (20 mL) and cooled to –35 °C for 3 days to give yellow crystals mixed with black precipitate. The supernatant was decanted and processed as described below.* The crystals were redissolved in MeCN (4.0 mL), the solution was filtered over Celite, layered with Et_2O (6 mL) and cooled to –35 °C for 3 days to give bright yellow crystals of compound **3**, which were decanted and dried *in vacuo* to give 26 mg, 55% yield (ie. expected yield for this dimer is half the moles (n) of **1** used multiplied by the expected stoichiometry of **3** relative to **1** (⅓) = ½n**1**•⅓ = 0.038 mmol, 47 mg).

*The solvent was removed from the supernatant *in vacuo*. The residue was redissolved in a minimum amount of MeCN (ca. 3 mL), filtered over Celite, then layered with Et_2O (ca. 7 mL) and cooled to –35 °C for 3 days to give orange crystals of compound **2**, which were dried *in vacuo* to give 62 mg, 67% yield (ie. expected yield for this dimer is half the moles (n) of **1** used multiplied by the expected stoichiometry of **2** relative to **1** (⅔) = ½n**1**•⅔ = 0.077 mmol, 92 mg). Note that **2** generated using this method is contaminated with **3** as a 3% impurity.

Note: this procedure was scaled to 1.849 g, 4.08 mmol of **1** and resulted in 0.987 g, 60% of isolated **2** (ie. expected yield for this dimer is half the moles (n) of **1** used multiplied by the expected stoichiometry of **2** relative to **1** (⅔) = ½n**1**•⅔ = 1.36 mmol, 1.635 g) and 0.491 g, 58% yield of isolated **3** (ie. expected yield for this dimer is half the moles (n) of **1** used multiplied by the expected stoichiometry of **3** relative to **1** (⅓) = ½n**1**•⅓ = 0.68 mmol, 839.7 mg). A minimum amount of MeCN, layered with an equal volume of Et_2O was used for each recrystallization at this scale.

Figure S2: ^1H NMR spectrum (300 MHz) in CD_3CN of the stoichiometric reduction of SF_6 with **1** in the presence of LiOTf. Inset zoom of aromatic region showing a 2:1 ratio of **2** and **3**.

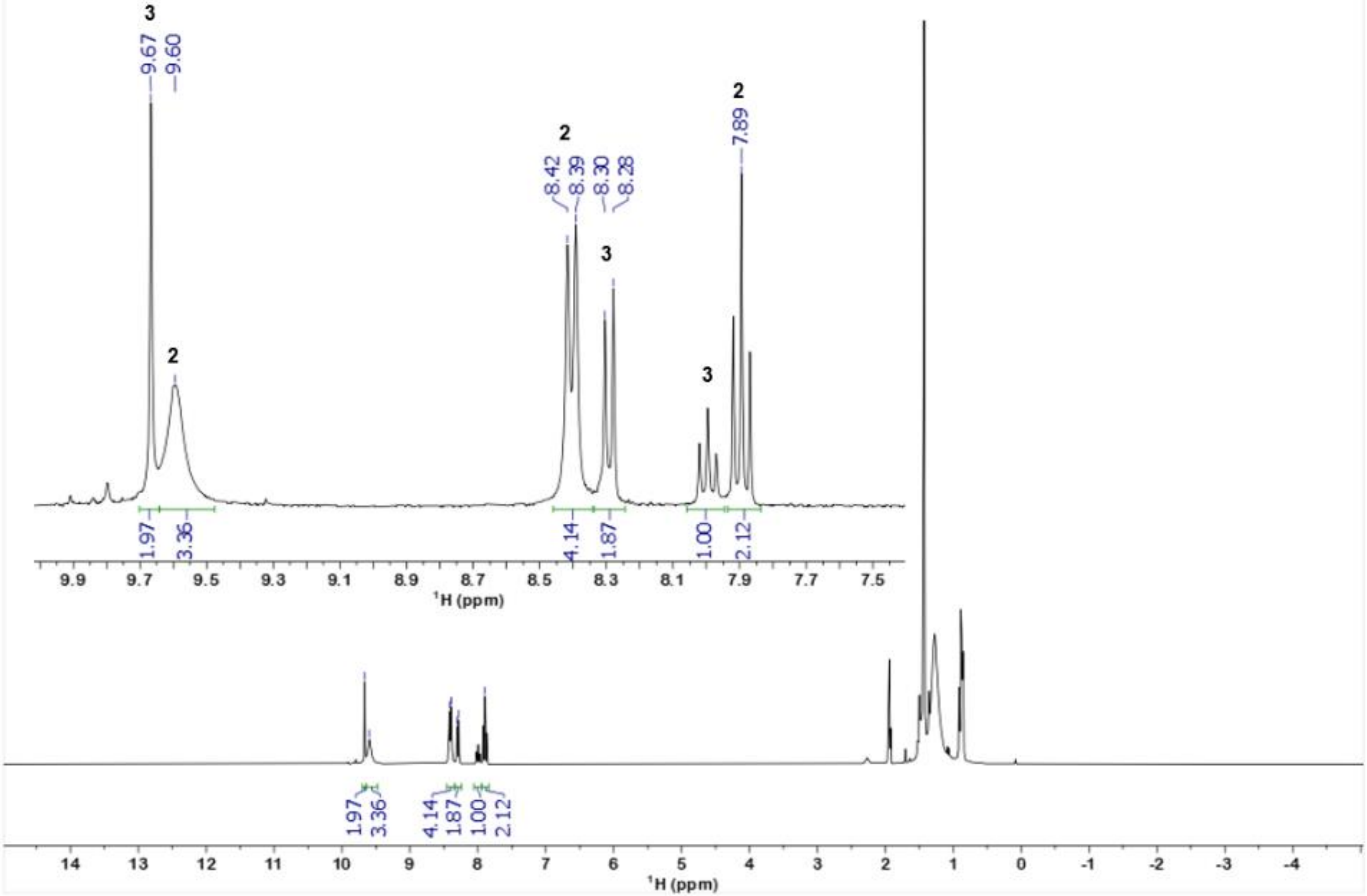

2.2 Alternative Synthesis of **2**.

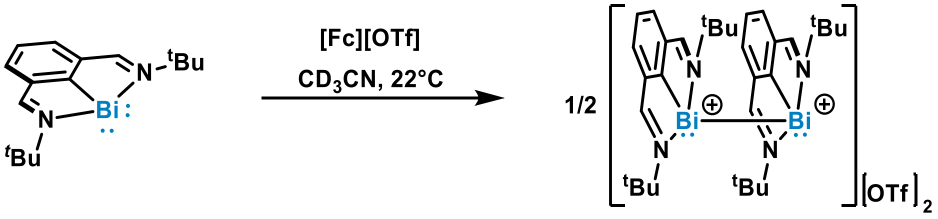

1 **2**

62%

Compound **1** (68 mg, 0.15 mmol, 1.0 equiv.) and ferrocenium triflate (FcOTf; 51 mg, 0.15 mmol, 1.0 equiv.) were combined as solids in a vial in the glovebox, then MeCN (6 mL) was added at 22 °C. The solution changed from dark blue to dark yellow within seconds. The vial was capped with a screw top septum cap, pierced with a needle and transferred into a large-neck Schlenk flask so that the volatiles could be removed *in vacuo*. The resulting solids were washed with Et_2O (5 × 4 mL) until the washings were colourless. The resulting yellow solids were recrystallized by layering a solution of MeCN (2 mL) with Et_2O (10 mL) and cooling to –35 °C for 3 days. The supernatant was decanted and the red crystals dried to give 56 mg of **2** in 62% yield.

2.3 Characterization Data of bis-[N,N-di-*tert.*-butyl-isophthalaldimine-2-yl-bismuthenium] bistriflate (**2**)

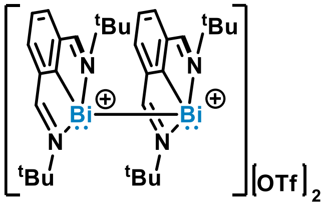

Single crystals suitable for single crystal X-ray diffraction studies were grown from an MeCN solution of **2** layered with Et_2O at –35 °C. This compound is yellow, but can appear red in crystalline form.

^{1}H NMR (600 MHz): δ = 9.59 (br s, 4H), 8.40 (br d, $^{3}J_{HH}$ = 8 Hz, 4H), 7.89 (t, $^{3}J_{HH}$ = 8 Hz, 4H), 1.28 (br s, 36H) ppm.

^{13}C{^{1}H} NMR (150.9 MHz): δ = 185.5, 171.1, 150.8, 138.5, 132.0, 122.2 (q, $^{1}J_{CF}$ = 320.9 Hz), 62.5 (br), 31.6 (br) ppm.

^{19}F NMR (564.8 MHz): δ = –79.2 (s) ppm.

Elemental Analysis found (calculated): C 33.77% (33.95%), H 3.83% (3.85%), N 4.63% (4.66%), S 5.29% (5.33%). Note: no characteristic *m/z* signals could not be detected for this compound using electrospray, photoionization or matrix assisted laser desorption ionization techniques. Elemental analysis was conducted to corroborate the chemical composition found by crystallography.

M.p. The compound does not melt, but begins to decompose at 287 °C.

2.4 Alternative Synthesis of **3**.

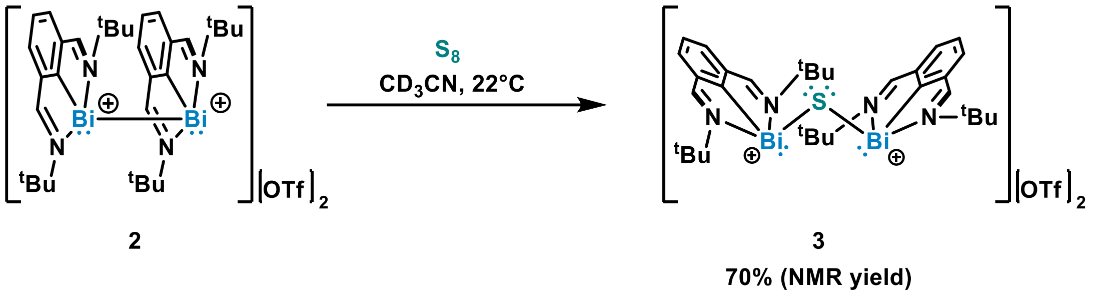

In a vial in the glovebox, compound **2** (11 mg, 0.0092 mmol, 1.0 equiv.) and trimethoxybenzene (3 mg, 0.02 mmol, 2 equiv.) were combined in CD_3CN. Then, this solution was added to solid elemental sulfur (3 mg, 0.01 mmol (S_8), 1 equiv.) in an NMR tube. A ^{1}H NMR spectrum was measured immediately, and the reaction was monitored every 5 min for the first 4 h, then every 15 min for a total of 15 h. The NMR yield of **3** was determined to be 70% after 10 h. The contents of the NMR tube were emptied into a Schlenk flask, the volatiles were removed and ESI-MS of the residue was collected. A signal at 468.2 m/z ($[C_{32}H_{46}N_4SBi_2]^+$) could be identified to corroborate the presence of **3**.

Figure S3: Conversion plot for the insertion of elemental sulfur into the Bi–Bi bond in **3** over the course of 14 h. Speculatively, the unknown species (data points marked with a blue cross – x) could be an S_8 insertion product,[7] or a higher oxidation state Bi species, but structural evidence was not obtained. The internal standard (data points marked with a red cross – x) is set to 206% as 2.06 equivalents were used.

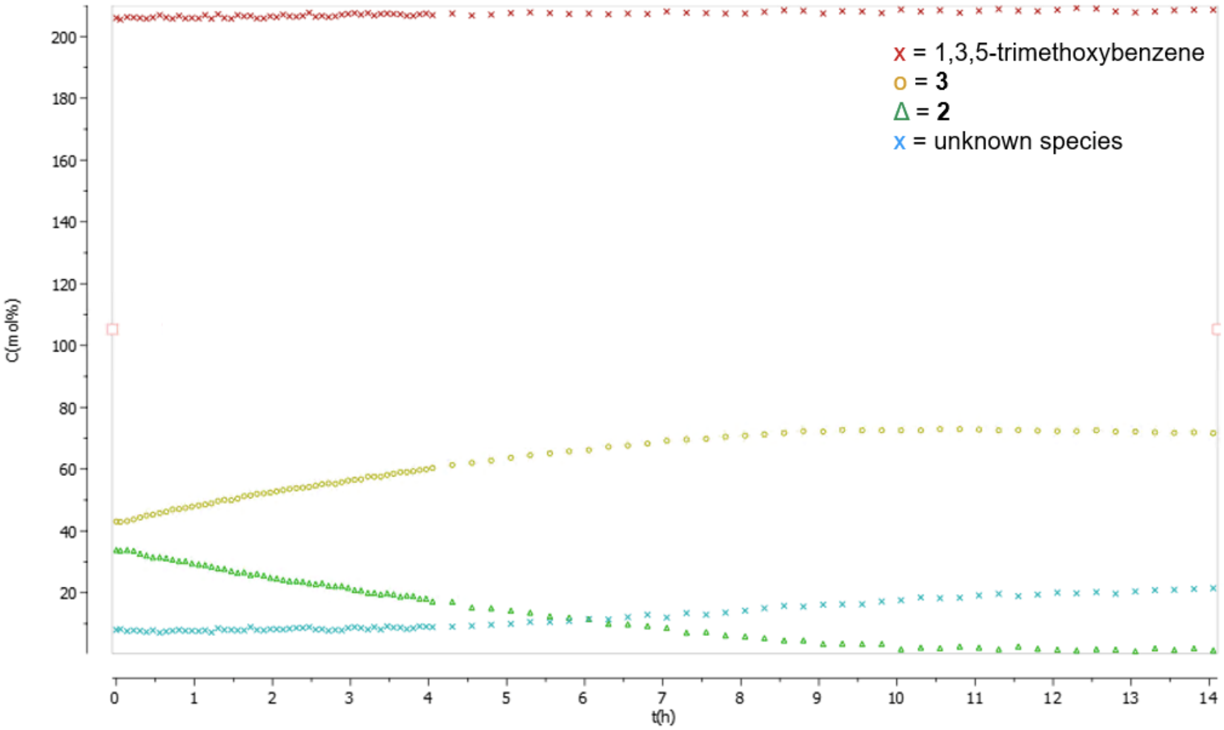

Figure S4: Stacked ^{1}H NMR spectra (500 MHz, CD_3CN) recorded over the course of 15 h to monitor the insertion of elemental sulfur into the Bi–Bi bond in **3**. C = 1,3,5-trimethoxybenzene (red, internal standard), **3** (yellow), C2 = **2** (green), ? = unknown species (blue).

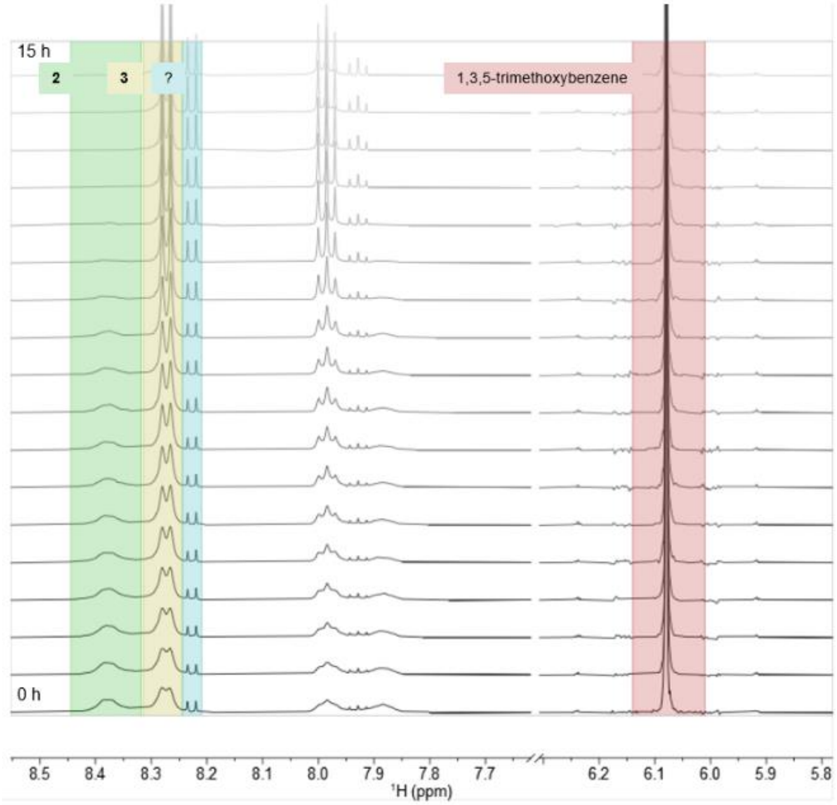

2.5 Characterization Data for bis-[N,N-di-*tert.*-butyl-isophthalaldimine-2-yl-bismuthenium]-μ_2-monosulfide bistriflate (**3**)

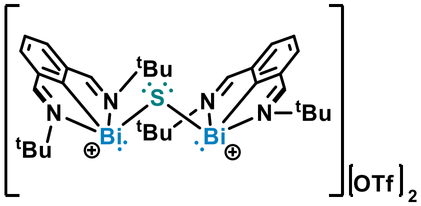

Single crystals suitable for single crystal X-ray diffraction studies were grown by vapor diffusion of Et_2O into an MeCN solution of **3** at –35 °C.

^{1}H NMR (600 MHz, CD_3CN): δ = 9.66 (s, 4H), 8.29 (d, $^{3}J_{HH}$ = 6 Hz, 4H), 8.00 (t, $^{3}J_{HH}$ = 6Hz, 2H), 1.43 (s, 36H) ppm.

^{13}C{^{1}H} NMR (150.9 MHz, CD_3CN): δ = 202.9, 169.4, 150.1, 138.1, 131.7, 122.0 (q, $^{1}J_{CF}$ = 316.9 Hz), 62.6, 31.4 ppm.

^{19}F NMR (564.8 MHz, CD_3CN): δ = –79.3 (s) ppm.

Positive ion ESI-MS found (calculated): $[C_{32}H_{46}N_4SBi_2]^{2+}$ ($[M]^{2+}$) m/z 468.1522 (468.1520).

Negative ion ESI-MS found: $[CO_3F_3S]^{-}$ ($[M]^{-}$) m/z 149.0.

M.p. The compound does not melt, but begins to decompose at 270 °C.

2.6 Preparation of **2** and **4**.

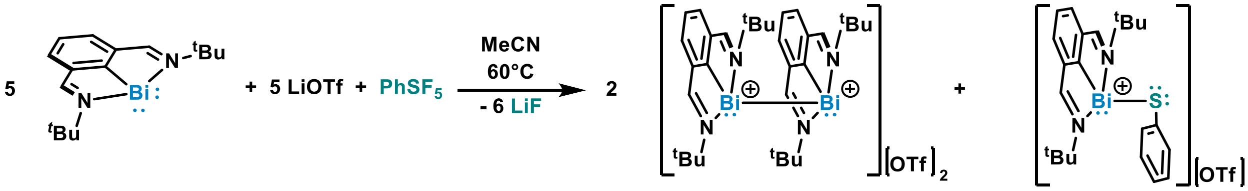

1 2 4

51 % 16 %

Lithium trifluoromethanesulfonate (90 mg, 0.57 mmol, 1.0 equiv.), $PhSF_5$ (282 mg, 1.38 mmol, 2.5 equiv.) and **1** (252 mg, 0.560 mmol, 1.0 equiv.) were dissolved in MeCN (30 mL) and the solution was loaded into a pressure Schlenk flask bearing a Teflon plug valve. The dark teal solution was stirred for 72 h at 60 °C and showed a gradual colour change to yellow-brown and a small amount of black precipitate. The volume of the reaction mixture was reduced to ca. 5 mL, filtered over Celite, layered with Et_2O (3 mL) and cooled to –35 °C for 72 h to give red crystals. The supernatant was decanted and processed as described below.* The crystals were redissolved in MeCN (8 mL) and layered with Et_2O (8 mL), then cooled to –35 °C to regrow crystals of **2**. The crystals were decanted, washed with Et_2O (3 × 3 mL), then dried *in vacuo* to give 138 mg of **2** in 51% yield (ie. expected yield for this dimer is half the moles (n) of **1** used multiplied by the expected stoichiometry of **2** relative to **1** (⅘) = ½n**1**•⅘ = 0.224 mmol, 269.4 mg). Note that this method of generating **2** yields analytically pure material (compared to the route using SF_6).

*The solvent was removed from the supernatant *in vacuo*. PhMe (4 mL) was added to the residue followed by THF (1 mL), then the solution was filtered over Celite, layered with pentane (12 mL) and cooled to –35 °C for 72 h to give crystals of **4**. The crystals were decanted, washed with pentane (3 × 1 mL), then dried *in vacuo* to give 13 mg of **4**, 16% yield (ie. expected yield the moles (n) of **1** used multiplied by the expected stoichiometry of **4** relative to **1** (⅕) = n**1**•⅕ = 0.112 mmol, 79.6 mg).

Ratio of 2 and 4 in the reaction mixture: Lithium trifluoromethanesulfonate (8 mg, 0.05 mmol, 1 equiv.), $PhSF_5$ (18 mg, 0.088 mmol, 2.0 equiv.) and **1** (20 mg, 0.044 mmol, 1.0 equiv.) were dissolved in CD_3CN (0.7 mL), the solution was loaded into a J-Young tube, heated to 60°C for 72 hours, then a proton NMR spectrum was measured. The reaction mixture displays signals for **2**, which are shifted from the pure compound. Meanwhile, the reaction mixture does not show any evidence for the pincer ligand backbone signals in **4**, however the thiophenolate aryl signals are present and align exactly with those in the pure compound (Figure S5). To inspect if the mixture of compounds has an effect of the chemical shifts of **2** and **4**, solid **2** (8 mg, 0.007 mmol, 1.8 equiv.) and **4** (3 mg, 0.004 mmol, 1.0 equiv.) were combined and dissolved in MeCN-d_3. The ^{1}H NMR spectrum revealed broadened signals for **2**. As in the above reaction mixture, the thiophenolate signals for **4** remained sharp and aligned with those in the pure compound, but the pincer ligand backbone signals disappeared (Figure S6). The main conclusion is that the signals of **2** and **4** are affected by whether the compounds are pure, or mixed. This explains why the reaction mixture spectrum does not align with those of pure **2** and **4**. With the nature of the mixed species NMR spectrum confirmed, the ratio of compounds **2** and **4** in the reaction mixture was determined to be 2:1 based on integrations of the meta protons in **2** (8.28 ppm, d, $^3J_{HH}$ = 12 Hz, 4H) relative to the thiophenolate meta protons in **4** (6.92 ppm, m, 1H; Figure S5).

Figure S5: ^1H NMR spectrum (300 MHz) in CD_3CN of the stoichiometric reduction of $PhSF_5$ with **1** in the presence of LiOTf. Inset zoom of aromatic region showing a 2:1 ratio of **2** and **4**.

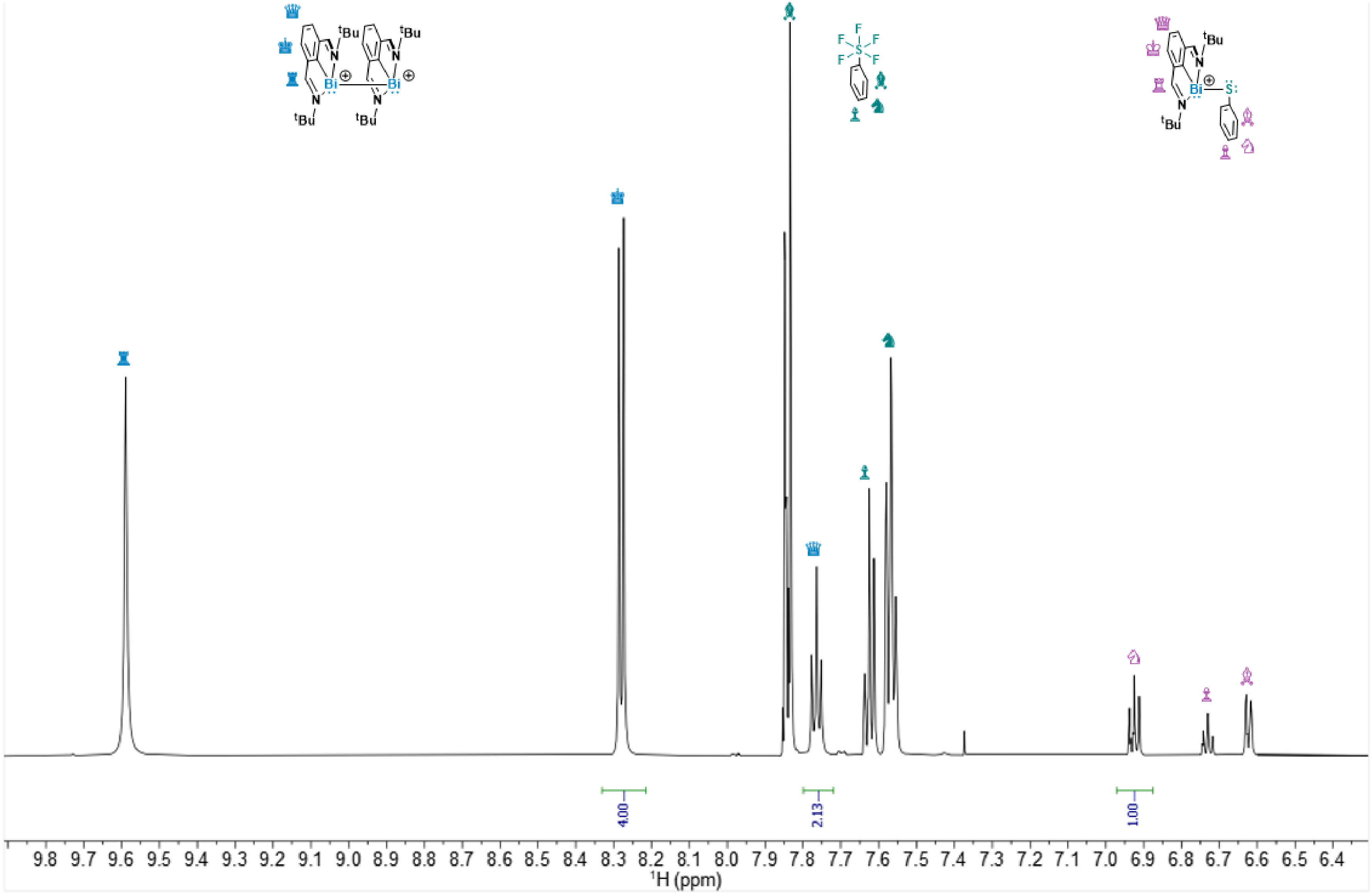

Figure S6: Stacked ^{1}H NMR spectra (600 MHz) in CD_3CN of the stoichiometric reduction of $PhSF_5$ with **1** in the presence of LiOTf, a 2:1 mixture of **2** and **4**, pure **4**, pure **2** and $PhSF_5$ (bottom to top).

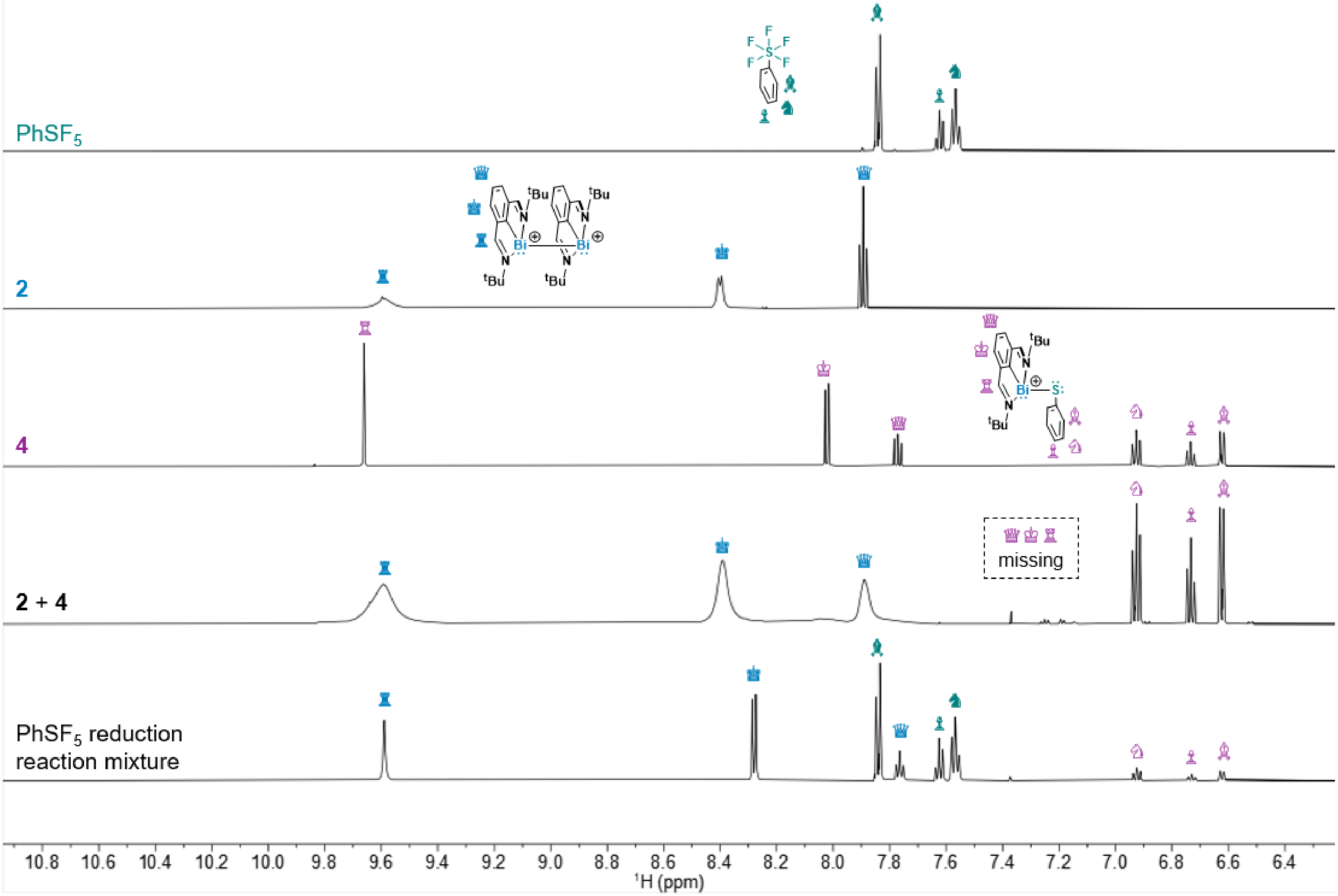

2.7 Alternative Synthesis of **4**.

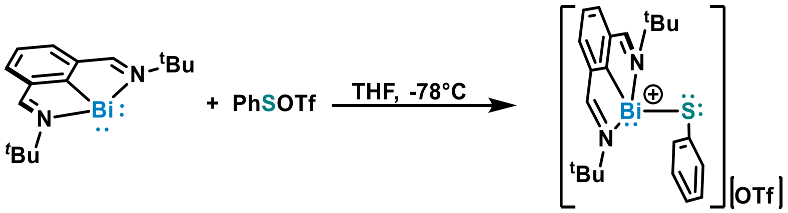

1 **4**

91 %

Benzene sulfenyl triflate (PhSOTf) was freshly prepared following a modified literature procedure.[8] Briefly, a stock solution of PhSCl in THF (0.174 M, 1.0 mL, 0.174 mmol, 1.04 equiv.) was transferred to a Schlenk flask containing a solution of thallium triflate (72 mg, 0.20 mmol, 1.2 equiv.) in THF (10 mL) at –78 °C. The solution was then removed from the cold bath and allowed to gradually warm for 20 min to give a cloudy yellow suspension. This solution was then added via cannula transfer to a -78 °C solution of **1** (76 mg, 0.17 mmol, 1.0 equiv.) in THF (5 mL) and warmed to 25 °C overnight to give a yellow solution. The reaction mixture was filtered over Celite and the volatiles removed *in vacuo*. The resulting yellow residue was triturated with pentane (3 × 5 mL), then dried *in vacuo* to give 125 mg of analytically pure **4** in 91% yield as a yellow solid.

2.8 Characterization Data for [N,N-di-*tert.*-butyl-isophthalaldimine-2-yl-bismuthenium]-thiophenolate triflate (**4**)

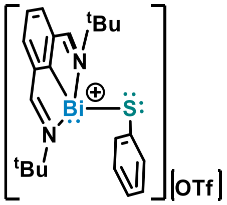

Single crystals suitable for single crystal X-ray diffraction studies were grown by vapor diffusion of pentane into a PhMe solution of **4** at –35°C.
^{1}H NMR (600 MHz, CD_3CN): δ = 9.66 (s, 2H), 8.02 (d, *J* = 7.5 Hz, 2H), 7.77 (dd, *J* = 7.5 Hz, 1H), 6.93 (m, 2H), 6.74 (m, 1H), 6.62 (m, 2H), 1.55 (s, 18H) ppm.
^{13}C{^{1}H} NMR (150.9 MHz, CD_3CN): δ = 202.1, 168.5, 149.5, 137.2, 136.6, 133.1, 131.2, 129.0, 128.3, 122.0 (q, $^1J_{CF}$ = 316.9 Hz), 62.5, 31.1 ppm.
^{19}F NMR (564.8 MHz, CD_3CN): δ = –79.4 (s) ppm.
Positive ion ESI-MS found (calculated): $[C_{22}H_{28}N_2SBi]^+$ ($[M]^+$) m/z 561.1775 (561.1772).
Negative ion ESI-MS found: $[CO_3F_3S]^-$ ($[M]^-$) m/z 149.0.
M.p. The compound does not melt, but begins to decompose at 207 °C.

3. Stoichiometric Reductions at Bi

3.1 Stoichiometric Reduction of **2**

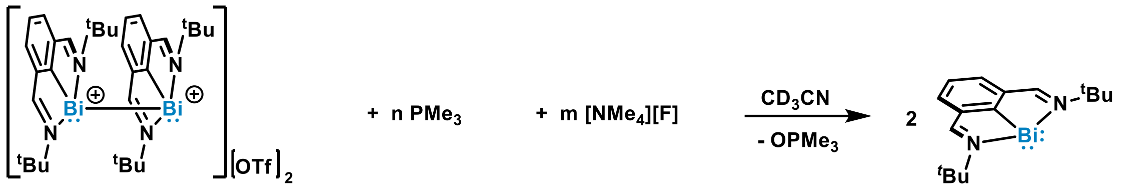

2 n = 1, or 10 m = 0, or 5.8 **1**

>95 %

Stoichiometric: A CD_3CN solution containing trimethyl phosphine (0.11 mL 0.31 M stock solution, 0.034 mmol, 0.97 equiv.) and 4-fluoroanisol (0.33 M, 0.036 mmol, 1.0 equiv.) was added to a yellow solution of **2** (42 mg, 0.035 mmol, 1.0 equiv.). The solution quickly darkened to yellow-brown, then slowly changed to dark green overnight. By ^{1}H NMR spectroscopy of the reaction mixture, the ligand backbone signals were observed to be slightly shifted from **2**, but no spectroscopic evidence for the formation of **1** could be observed. On top of this, PMe_3 (δ_P = -60 ppm) was the only species observed by $^{31}P\{^1H\}$ NMR spectroscopy.

Excess PMe3: A CD_3CN solution of trimethyl phosphine (15 mg, 0.19 mmol, 11 equiv.) was added to a yellow solution of **2** (21 mg, 0.018 mmol, 1.0 equiv.) and trimethoxybenzene (31 mg, 0.18 mmol, 10 equiv.). The solution quickly became brown. The ^{1}H NMR spectrum of the reaction mixture revealed broad ligand backbone signals, where the chemicals shifts of the aldimine (s), *meta* (d) and *para* (t) protons appear at chemical shifts that are intermediate to those for **1** and **2** (see Figure S7), indicating a possible equilibrium between the two species.

Figure S7: Stacked ^{1}H NMR spectra (300 MHz, MeCN-d_3) showing the aromatic region for **1**, **2** and the reaction mixture of **2** with an excess of PMe_3.

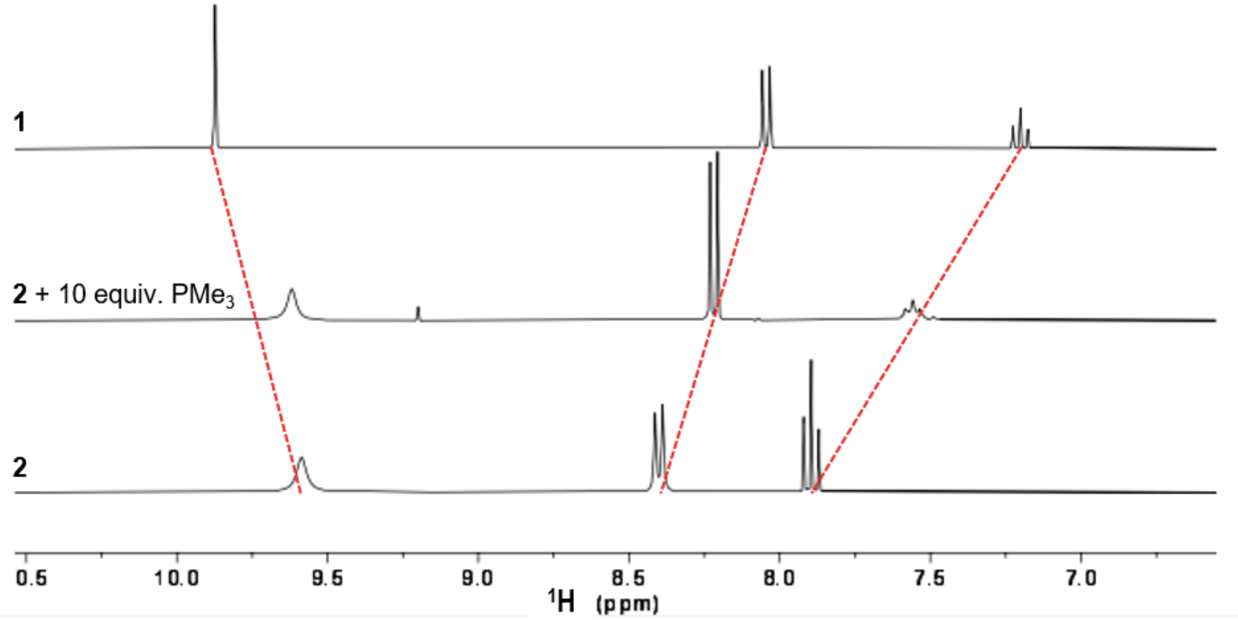

Stoichiometric with additive: A CD_3CN solution containing PMe_3 (0.50 mL, 0.037 M stock solution, 0.018 mmol, 1.2 equiv.) and trimethoxybenzene (0.033 M, 0.016 mmol, 1.0 equiv.) was transferred from a volumetric flask to an NMR tube containing a yellow solution of **2** (20 mg, 0.016 mmol, 1.0 equiv.) and $[NMe_4][F]$ (9 mg, 0.09 mmol, 6 equiv.). The orange brown solution quickly became dark brown, then dark teal and was allowed to stand at room temperature for 4 h. The NMR yield of **1** was determined to be >95% by integrations relative to the internal standard. $^{31}P\{^1H\}$ NMR spectroscopy reveals the only phosphorus containing species in the reaction mixture to be $OPMe_3$ (δ_P = 36.9 ppm). It is likely that the expected $(OTf)_2PMe_3$ byproduct hydrolyzes in the presence of residual water sourced from the ammonium salt (undried).

3.2 Stoichiometric Reduction of **3**

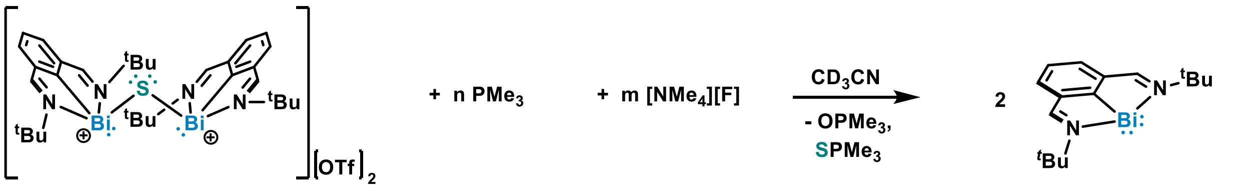

3 n = 2, or 20 m = 0, or 8.8 1

67 %

Stoichiometric: A CD_3CN solution containing trimethyl phosphine (0.25 mL 0.31 M stock solution, 0.078 mmol, 2.0 equiv.) and 4-fluoroanisol (0.33 M, 0.083 mmol, 2.1 equiv.) was added to a yellow solution of **3** (49 mg, 0.040 mmol, 1.0 equiv.). The solution quickly darkened to yellow-brown, then slowly changed to dark green overnight. By ^1H NMR spectroscopy of the reaction mixture, the ligand backbone signals were observed to be slightly shifted from **3**, but no spectroscopic evidence for the formation of **1** could be observed. However, the $^{31}P\{^1H\}$ NMR spectrum showed signals for both the PMe_3 starting material (δ_P = -60 ppm) as well as $SPMe_3$ (δ_P = 30.9 ppm), indicating oxidative sulfide transfer from bismuth to phosphorus.

Excess PMe_3: A CD_3CN solution of trimethyl phosphine (29 mg, 0.38 mmol, 22 equiv.) was added to a yellow solution of **3** (20 mg, 0.017 mmol, 1.0 equiv.) and trimethoxybenzene (56 mg, 0.33 mmol, 20 equiv.). The solution quickly became brown. The ^1H NMR spectrum of the reaction mixture revealed the formation of a mixture of ligand-bearing products, the major of which showing a broadening of the aldimine proton singlet (δ_H = 9.60 ppm). On top of this, the *para* proton triplet (δ_H = 7.75 ppm) was broadened and shifted to an intermediate chemical shift between those for **1** (δ_H = 7.20 ppm) and **3** (δ_H = 8.00 ppm) (see Figure S8), indicating a possible equilibrium between the two species.

Figure S8: Stacked ^1H NMR spectra (300 MHz, MeCN-d_3) showing the aromatic region for **1**, **3** and the reaction mixture of **3** with an excess of PMe_3.

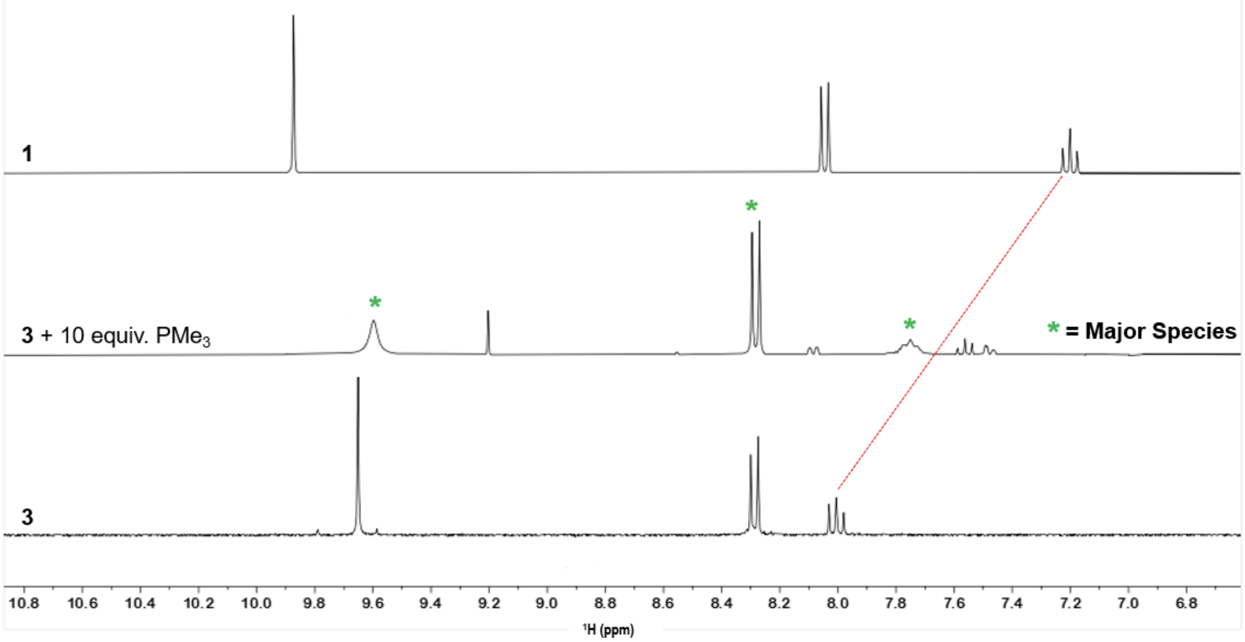

Stoichiometric with additive: A CD_3CN solution containing PMe_3 (1.0 mL, 0.037 M stock solution, 0.037 mmol, 2.2 equiv.) and trimethoxybenzene (0.033 M, 0.033 mmol, 1.9 equiv.) was transferred from a volumetric flask to a vial containing a yellow suspension of **3** (21 mg, 0.017 mmol, 1.0 equiv.) and $[NMe_4][F]$ (14 mg, 0.15 mmol, 8.8 equiv.). The bright yellow suspension quickly became dark teal with some yellow precipitate and was allowed to stand at room temperature until the yellow precipitate disappeared (ca. 4 h). An aliquot of the reaction mixture was taken to determine the NMR yield of **1** to be 67%. $^{31}P\{^1H\}$ NMR spectroscopy revealed the two phosphorus containing byproducts in the reaction mixture to be $OPMe_3$ and $SPMe_3$ (δ_P = 36.9 and 30.9 ppm, respectively). By ^1H NMR integrations relative to the internal standard, the NMR yields were determined to be 61% for $OPMe_3$ and >95% for $SPMe_3$. These NMR yields show that the reduction of the triflate portion could be accountable for the low recovery of **1** in this reaction.

3.3 Stoichiometric Reduction of 4

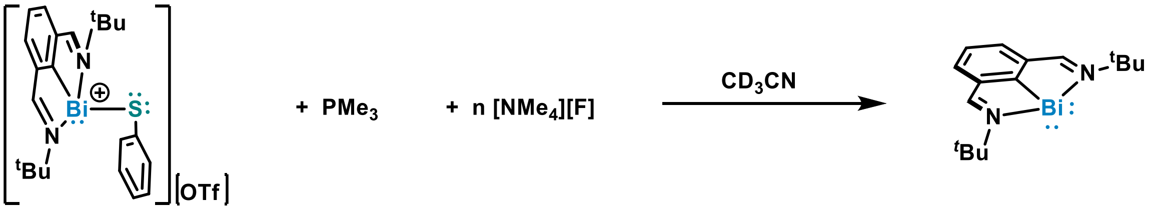

4 **n = 0, or 4.2** **1**

85 %

Stoichiometric without additive: A CD_3CN stock solution containing **4** (0.65 mL, 0.019 M stock solution, 0.013 mmol, 1.0 equiv.) and trimethoxbenzene (0.020 M, 0.013 mmol, 1.0 equiv.) was added to an NMR tube. The solution changed from yellow to orange. Then an acetonitrile solution

of trimethyl phosphine (0.1 mL, 0.15 M stock solution, 0.015 mmol, 1.2 equiv.) was added, the contents of the tube were shaken to give a transparent emerald green solution. After standing at room temperature overnight, the solution was dark teal. The ^{1}H NMR spectrum of the reaction mixture showed broadening of the aldimine singlet (δ_H = 9.69 ppm) and the thiophenolate protons as well as a slight shifting of the *para* proton triplet to higher field (δ_H = 7.66 ppm), although there is no spectroscopic evidence for the formation of **1** (see Figure S9). However, PMe_3 (δ_P = -60 ppm) and $OPMe_3$ (δ_P = 38 ppm) could be identified in the $^{31}P\{^{1}H\}$ NMR spectrum, indicating some form of redox process.

Figure S9: Stacked ^{1}H NMR spectra (300 MHz, MeCN-d_3) showing the aromatic region for **1**, **4** and the reaction mixture of **4** with stoichiometric PMe_3.

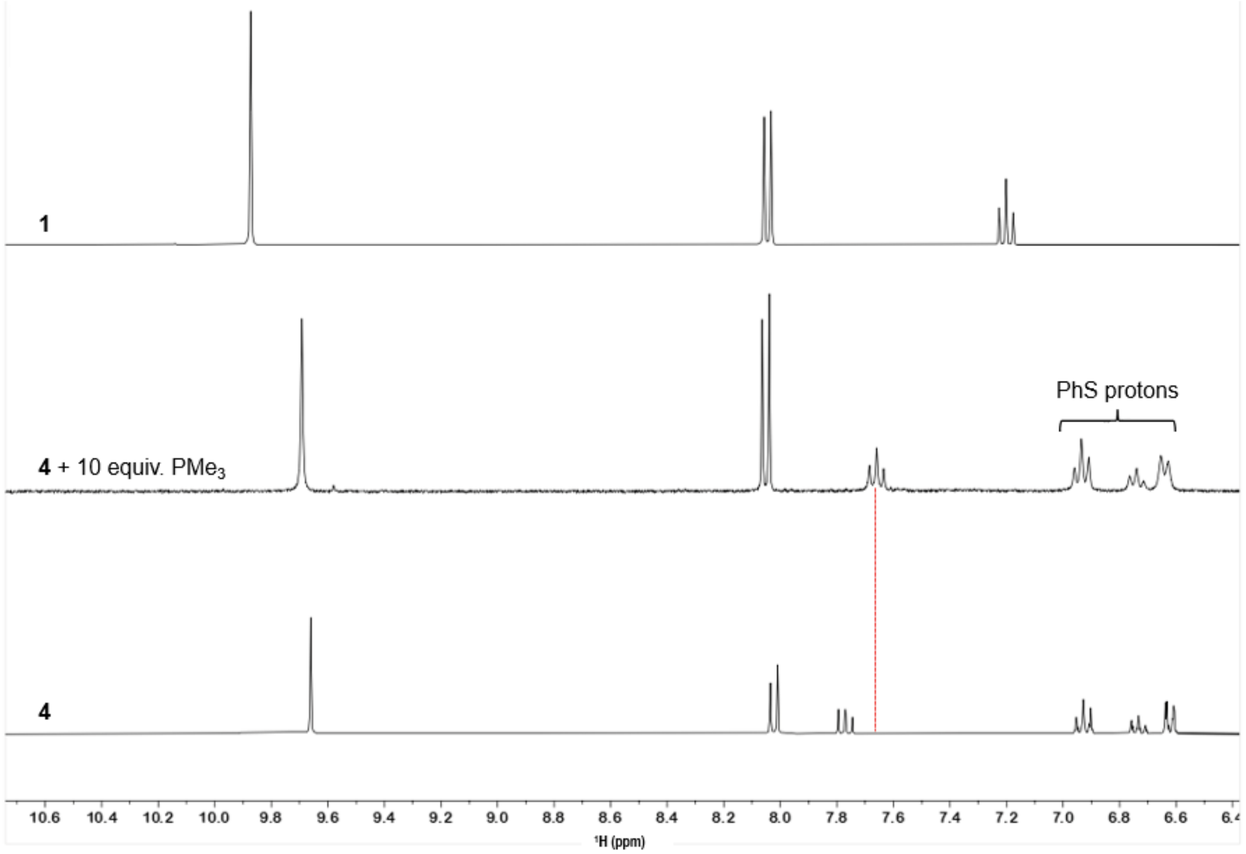

Stoichiometric with additive: A CD_3CN stock solution containing **4** (0.65 mL, 0.019 M stock solution, 0.013 mmol, 1.0 equiv.) and trimethoxbenzene (0.020 M, 0.013 mmol, 1.0 equiv.) was transferred from a volumetric flask to an NMR tube containing $[NMe_4][F]$ (5 mg, 0.05 mmol, 4 equiv.). The solution changed from yellow to orange. Then a MeCN solution of PMe_3 (0.10 mL, 0.15 M stock solution, 0.015 mmol, 1.2 equiv.) was added to give a gradient of colours from orange to green to colourless. The tube was shaken to give a green-brown solution and allowed to stand at room temperature for 18 h to give a dark teal colour. The NMR yield of **1** was determined to be 85%. At this point, broad signals could be observed (6.96–7.14 ppm), accounting for the displaced thiophenolate ($[SPh]^-$; Figures S11 and S12). The solution was allowed to stand at room temperature for an additional 27 h, at which point the $[SPh]^-$ signals sharpened (7.18, 6.99, 6.84 ppm). Negative NOE cross correlations could be observed with the $[NMe_4]^+$ protons (3.08 ppm;

Figure S13). The change of the signal shape in combination with negative NOESY correlations hint at the formation of ionic aggregates in solution with a reduced molecular mobility. After 45 h an equilibrium of these species in solution was reached resulting in sharper ^{1}H NMR signals. The $[SPh]^-$ protons do not show any NOE correlation with the signals of **1**.

Figure S10: NMR tubes showing the different colours through out the reduction of **4** with PMe_3. Left: compound **4** dissolved in CD_3CN (bottom) and **4** mixed with $[NMe_4][F]$ (top); middle: colour gradient from orange to dark green immediately after the addition of PMe_3; right: contents of the tube were shaken after adding PMe_3 (top), the tube was allowed to stand at room temperature overnight (bottom).

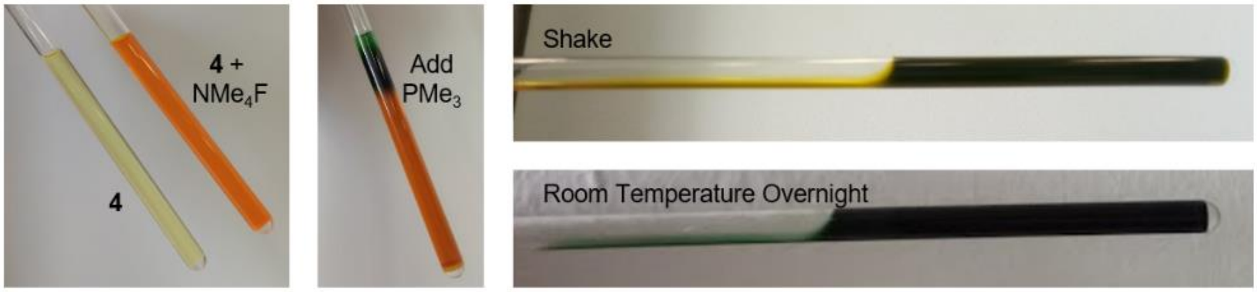

Figure S11: Stacked ^{1}H NMR spectra (300 MHz, CD_3CN) showing the aromatic region for **1**, **4** and each step towards reducing **4** to **1**.

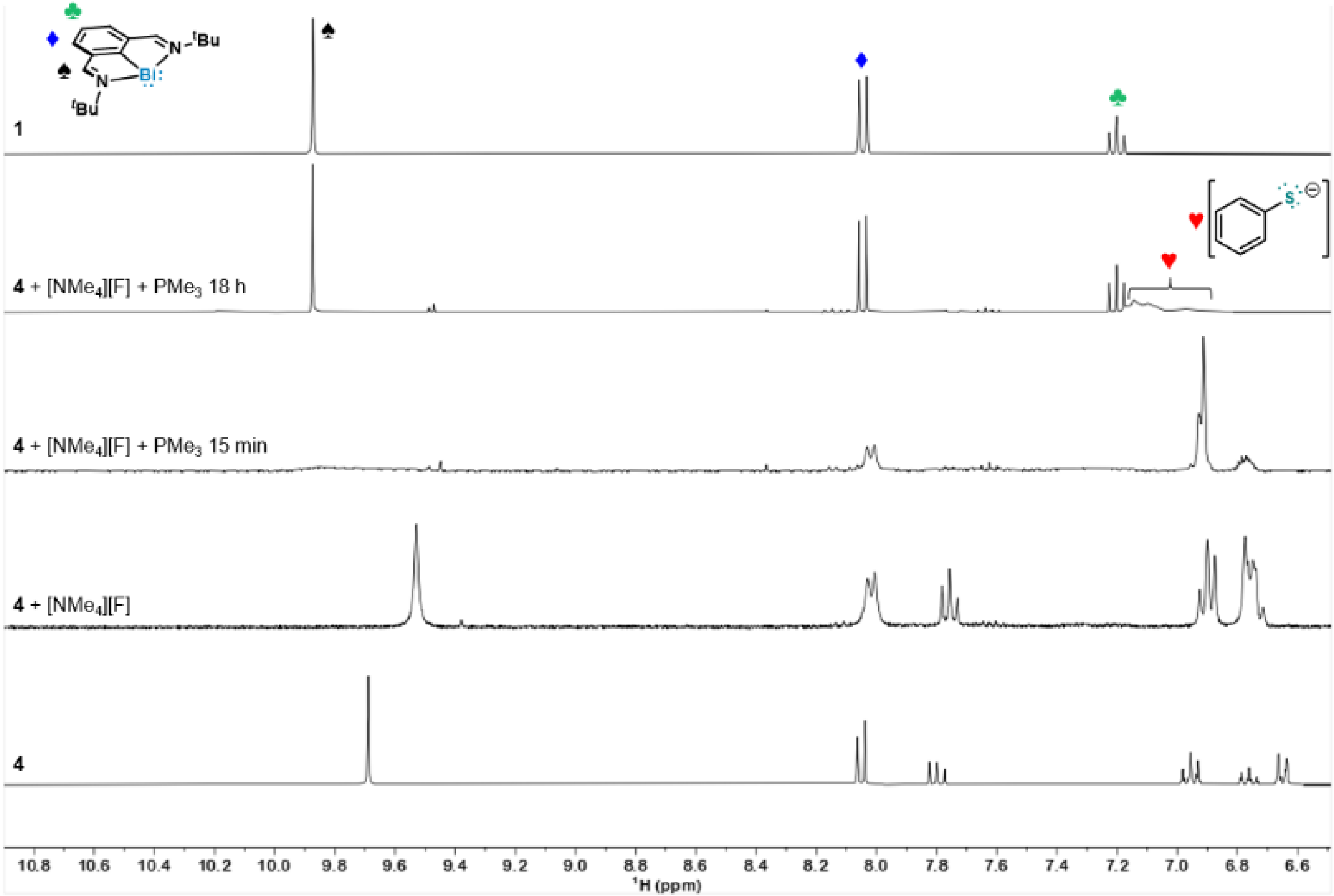

Figure S12: Stacked ^{1}H NMR spectra (600 MHz, CD_3CN) showing the $[SPh]^-$ and $[NMe_4]^+$ signals in the reaction mixture of the reduction of **4** to **1** with PMe_3 and $[NMe_4][F]$ after 2 and 45 h. Both sets of signals are initially broad and sharpen over time.

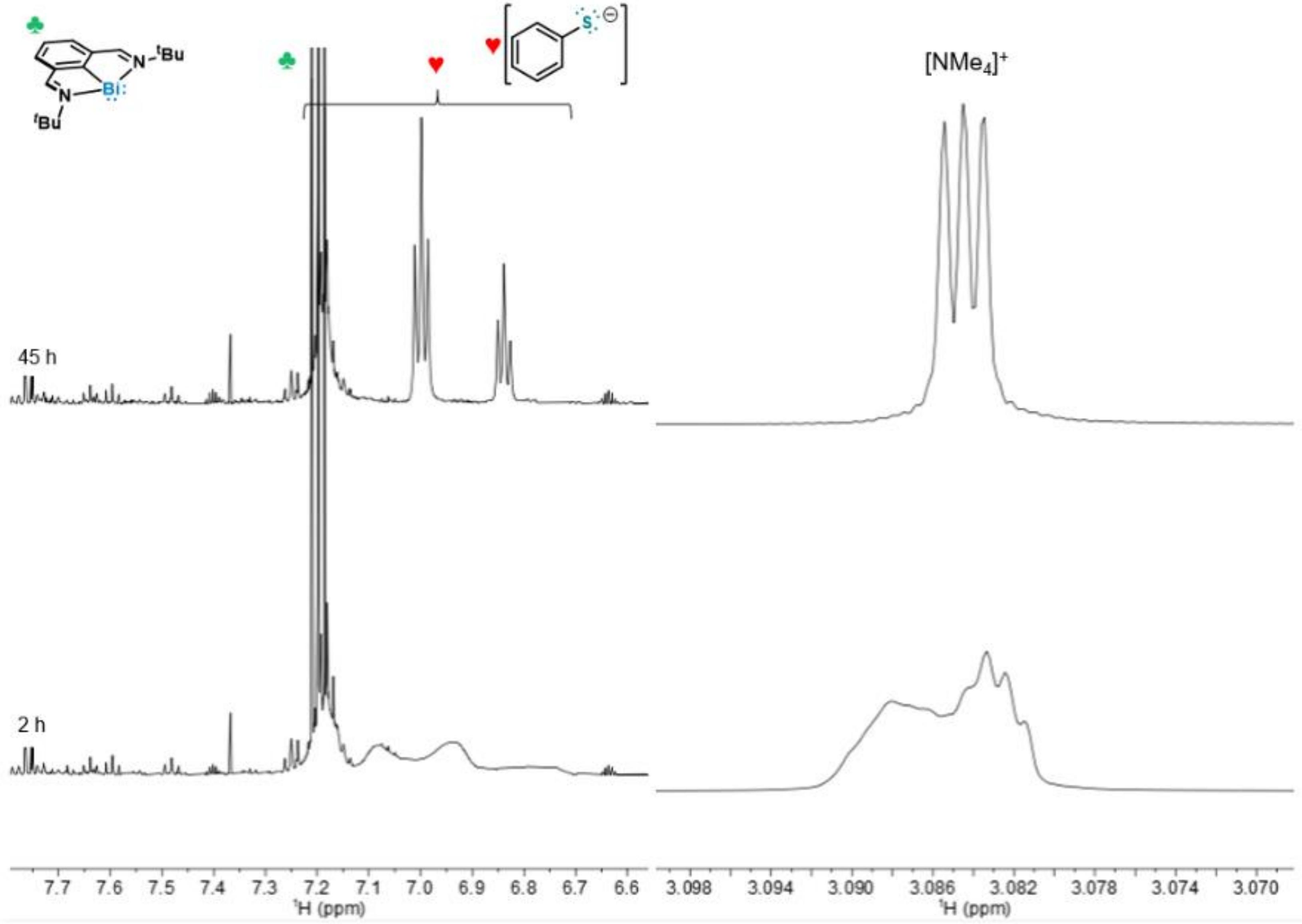

Figure S13: ^{1}H NOESY NMR spectrum (600 MHz, CD_3CN) for the reaction mixture of the reduction of **4** to **1** with PMe_3 and $[NMe_4][F]$ after 45 h. Positive correlations in black and negative correlations in red.

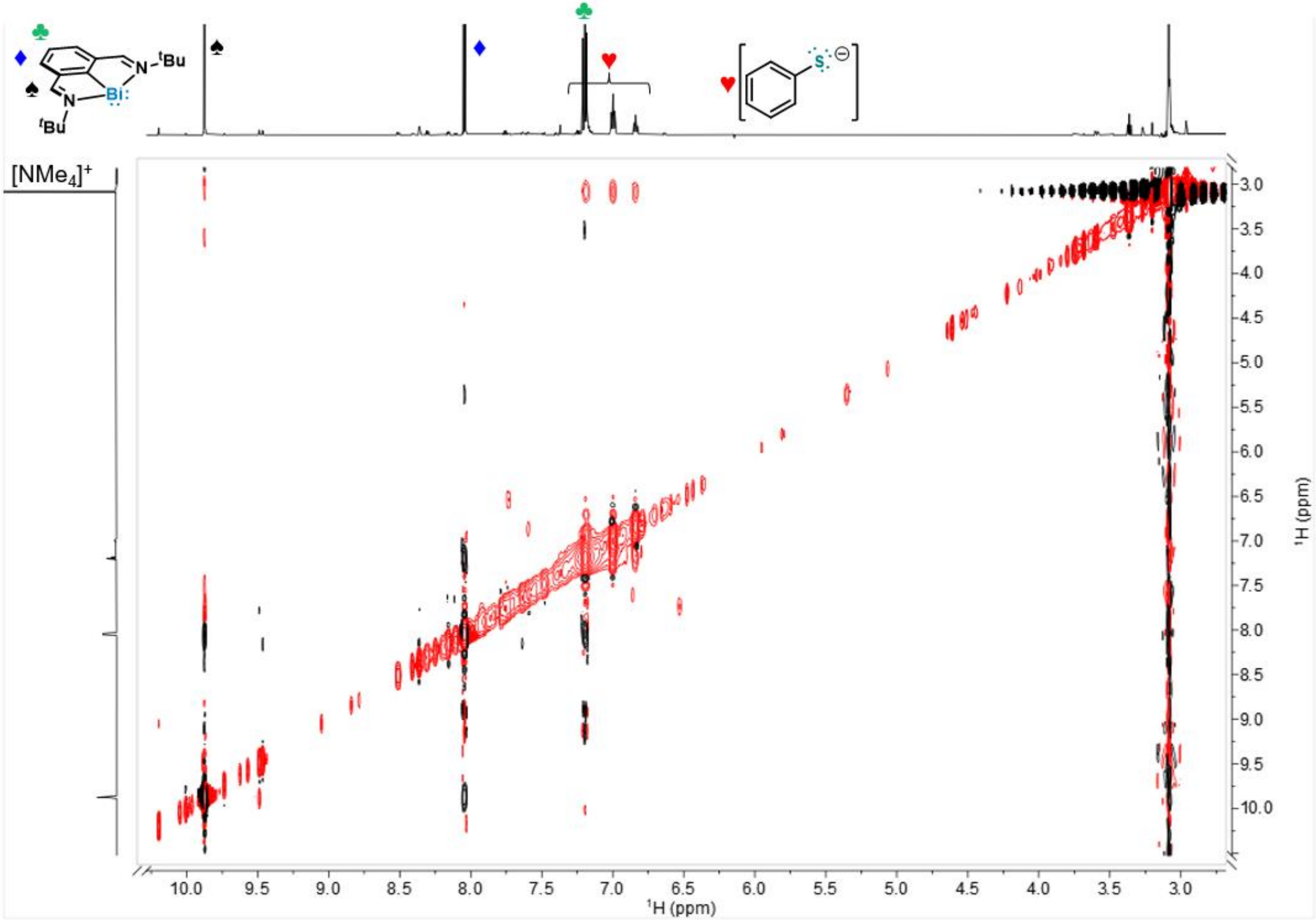

3.4 Stoichiometric Reduction of **1•[F₂]**

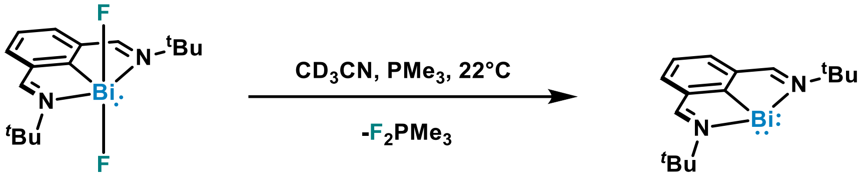

A MeCN stock solution (0.20 mL) containing PMe_3 (0.030 mmol, 0.15 M, 1.3 equiv.) and 4-fluoroanisole (0.028 mmol, 0.14 M, 1.2 equiv.) was transferred from a volumetric flask to an NMR tube containing a CD_3CN solution (0.5 mL) of **1•[F₂]** (11 mg, 0.023 mmol, 1.0 equiv.) at 22 °C. The solution was initially colourless and gradually changed to pale teal and finally to dark teal over the course of 3 h, at which point an NMR spectrum was acquired to reveal the presence of F_2PMe_3 by $^{31}P\{^1H\}$ NMR spectroscopy and a broadening of the **1•[F₂]** signals by ^1H NMR spectroscopy. The solution was allowed to stand at room temperature for an additional 18 h, at which point the NMR yields for **1** and F_2PMe_3 were determined to be 73% and 76%, respectively.

4. Disproportionation

4.1 Disproportionation of **2** using $[NMe_4][F]$

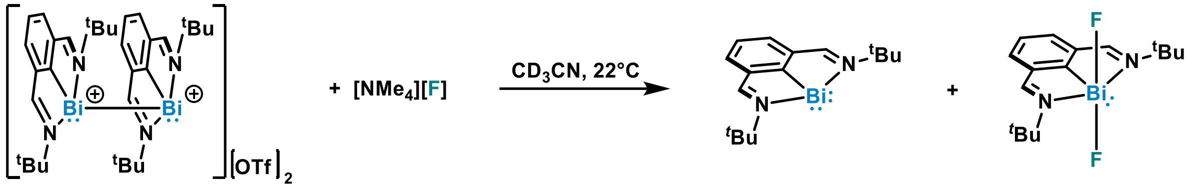

2 **1** >95% (NMR yield) **1•[F]2** 92% (NMR yield)

A stock solution of **2** in CD_3CN (0.0043 mmol, 0.0070 M, 0.60 mL, 1.0 equiv) was transferred from a volumetric flask to an NMR tube containing solid **$[NMe_4][F]$** (1 mg, 0.01 mmol, 2 equiv.) at 22°C. A colour change from yellow to olive green to dark green was observed in the first 5 min. The initial ^1H NMR spectrum of the reaction mixture revealed one set of broad signals and the tube was allowed to stand at room temperature for 12 h, at which point the colour was dark teal. A new ^1H NMR spectrum was recorded and signals in line with **1** and **1•$[F_2]$** were observed. A solution of 1,3,5-trimethoxybenzene in MeCN (0.0042 mmol, 0.021 M, 0.2 mL) was then added and the NMR yield was found to be >95% for **1** and 92% for **1•$[F_2]$**.

5. Cyclic Voltammetry

General considerations: Electrochemical measurements were performed in an argon-filled glovebox with a Gamry Interface 1010E Potentiostat using a 1.6 mm diameter glassy carbon working electrode, a platinum wire counter electrode, and a silver wire pseudoreference electrode. A 20 mL scintillation vial used as the cell was fitted with a Teflon cap with openings for the three electrodes. The electrode leads were connected through banana plugs to an electrode cable feedthrough port in the glovebox. All scans were references to the ferrocenium/ferrocene redox couple ($Fc^{+/0}$) by using ferrocene as an internal reference. Ohmic drop was minimized by minimizing the distance between working and reference electrodes, and by employing a preset current-interrupt iR correction on the Gamry software. Glassy carbon electrodes were polished with an alumina suspension (0.05 μm) in deionized water on a pre-wetted polishing pad using figure-eight motions, rinsed with deionized water, rinsed with ethanol, and then sonicated in ethanol for 20 seconds to remove additional polishing powder. Silver pseudoreference electrodes were lightly polished with fine grit sandpaper, washed with ethanol, dabbed with lint-free paper towels, and sonicated in ethanol for 30 seconds to remove any particulate. Each voltammogram reported herein was recorded with freshly polished electrodes. Before collecting data in the presence of the analyte of interest, conditioning cycles were performed by cycling the potential between 1.5 V and –2.0 V four times at 100 mV/s in the supporting electrolyte solution. In all cases, the third and fourth cycles were superimposable, indicating sufficient conditioning of the electrodes.

Complex **2** features a peak potential of –0.91 V on the cathodic wave and a quasi-reversible feature at –0.86 V, corresponding to the 1-electron $Bi^{II/I}$ redox couple (Figure S14). The latter assignment is supported by agreement with the reported cyclic voltammogram of **1**.[9]

Figure S14: Cyclic voltammograms at different scan rates of **2** in a 0.1 M solution of $[^nBu_4N][PF_6]$ in anhydrous degassed MeCN. Working electrode: 1.6 mm diameter glassy carbon; counter electrode: platinum wire; pseudo-reference electrode: silver wire.

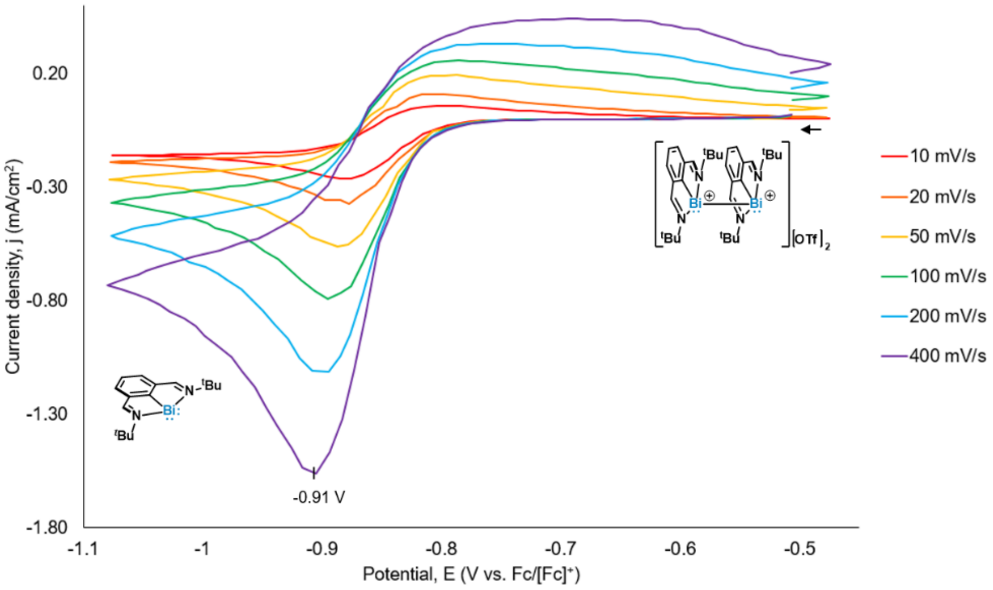

Figure S15: Cyclic voltammograms at different scan rates of **5** in a 0.1 M solution of [$^{n}Bu_4N$][PF_6] in anhydrous degassed MeCN. Working electrode: 1.6 mm diameter glassy carbon; counter electrode: platinum wire; pseudo-reference electrode: silver wire. A reversible oxidation peak corresponding to the $Bi^{I/II}$ redox step was observed at -1.01 V for bismuthinidene **5** vs. Fc/[Fc]$^+$.

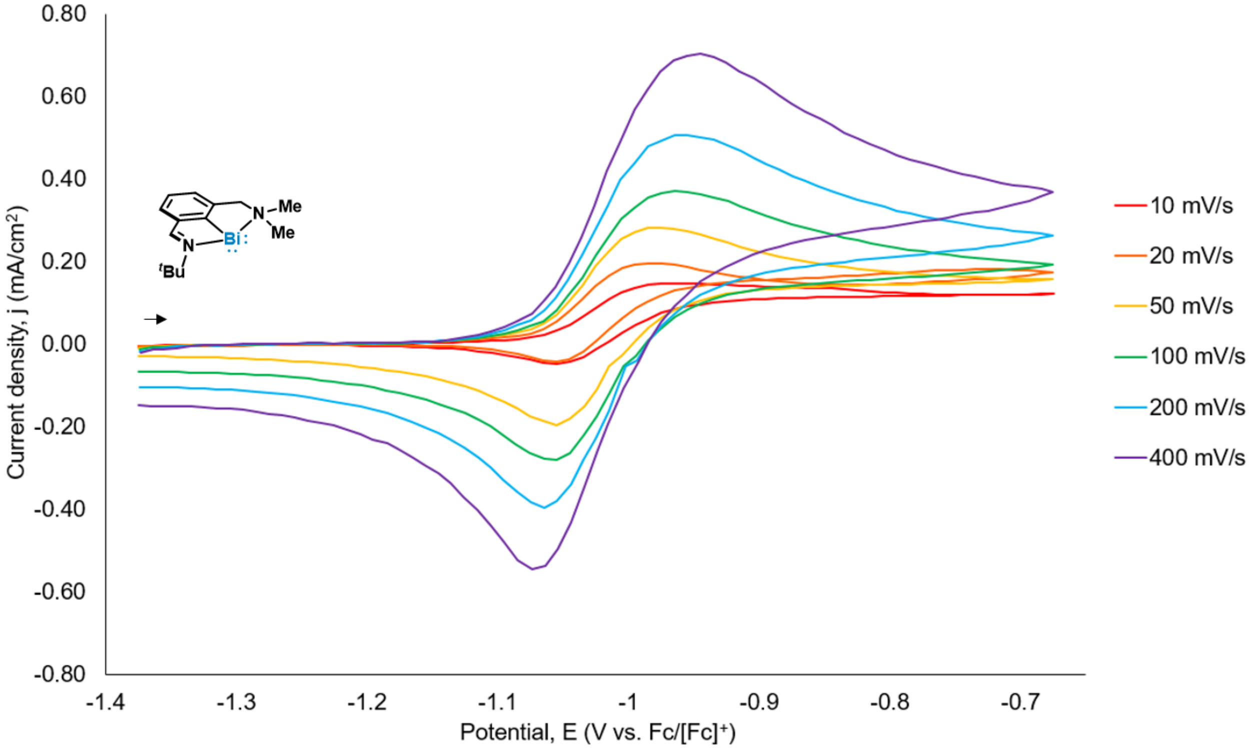

6. Catalytic Reaction Details

6.1 SF_6 Reactions

Procedure for flame sealing: Reaction mixtures for catalysis optimization were loaded into an NMR tube fused to a Schlenk adapter, sealed with a septum, exported from the glovebox and cycled onto a Schlenk line equipped with a three-way vacuum-argon-SF_6 line. The contents of the tube were frozen by immersion into a liquid nitrogen bath, the headspace of the tube was evacuated, then SF_6 was introduced. The NMR tube was marked with a permanent marker 7 inches from the bottom. SF_6 pressure was set to a minimum (0.1 bar gauge pressure) to allow for positive pressure while sealing, yet avoid bursting the molten glass. The septum was removed and the tube was sealed shut on the 7-inch mark using a butane hand-held torch. The sealed tubes are assumed to be under 1 bar(a) SF_6.

Figure S16: Demonstration of flame sealing under an active flow of SF_6.

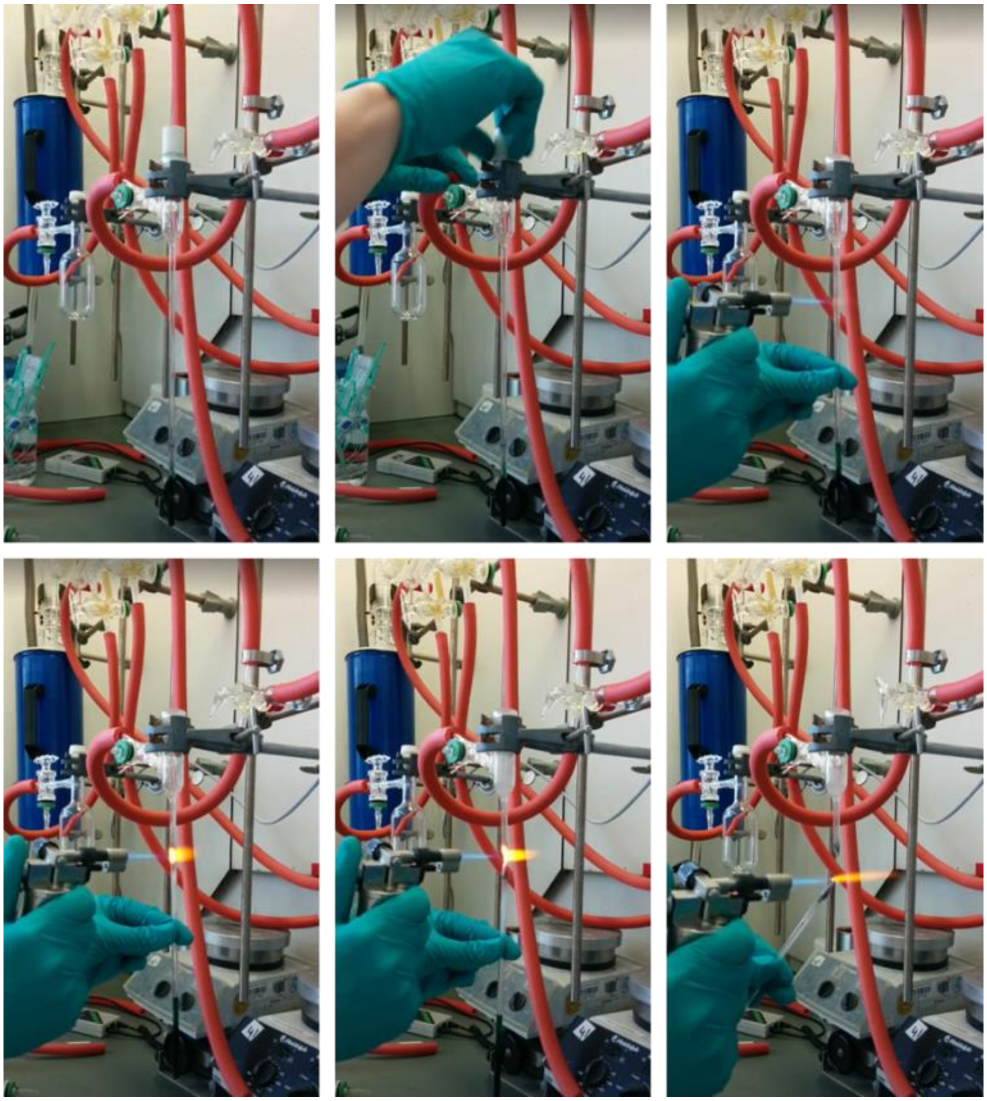

6.1.1 Notes on catalyst poisoning with inadequate reducing agents

While screening for appropriate conditions for the title catalytic manifold, the NCN-pincer bismuthinidene (**1**) behaved as Goldilocks when it came to reducing agent selection. Elemental reducing agents such as Mn, Zn, Mg, red phosphorus, Hg, and Al as well as CaH_2 were not effective (or 'too cold'), possibly due to solubility issues. Meanwhile, hydritic reducing agents such as lithium aluminum hydride, diisobutylaluminum hydride and potassium tri-*sec*-butylborohydride as well as phenyl lithium proved to be too harsh (or 'too hot') for the ligand framework when used in excess. The results from mild hydride sources are discussed in detail in this section. In short, it was found that turnover could be achieved for fluorine, but not sulfur, which resulted in catalyst poisoning. Trimethyl phosphine was found to be 'just right' as it was able to scavenge both F and S components, which was essential for preventing catalyst poisoning.

Mild hydride sources such as pinacol borane and triethyl silane ($HSiEt_3$) were effective at reducing the fluoride oxidative addition products as evidenced by the formation of H_2, fluoropinocolborane (δ_F = –150.8 ppm) and $FSiEt_3$ (δ_F = –175.2 ppm) by heteronuclear NMR spectroscopy. However, no evidence for sulfur-containing borane or silane byproducts could be identified. For $HSiEt_3$, although an increase in catalyst loading improved the rate of $FSiEt_3$ formation, the signals for **1** were observed to steadily disappear by ^{1}H NMR spectroscopy over time. The insoluble yellow precipitate formed in this reaction was analyzed by XPS to reveal the presence of C, N, O, F, Si, S and Bi (see SI). In particular, two sets of Bi $4f_{5/2}$ and $4f_{7/2}$ absorption edges are observed. The first set of signals (164.92 and 159.62 eV, respectively) align with Bi_2O_3.[10] The second set of signals (163.70 and 158.40 eV) appear between those for Bi_2O_3 and Bi_2Se_3 (*cf.* 162.9 and 157.6 eV).[11] Taken with the S $2p_{3/2}$ absorption edge (163.29 eV), which is akin to a bridging sulfide (*cf.* 163.3–164.8 eV),[12] indicate a bismuth sulfide species and corroborate catalyst poisoning by sulfur under these reaction conditions.

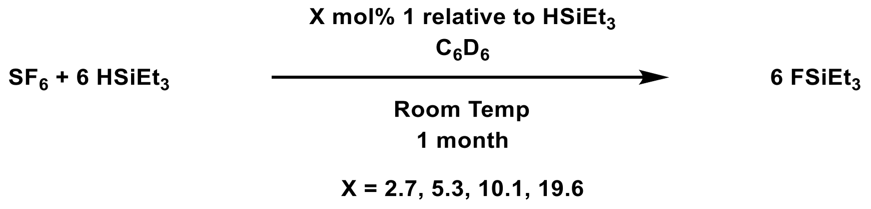

Effect of Catalyst Loading: Triethylsilane (70 µL, 0.44 mmol, 1.0 equiv.), 4-fluoroanisole (50 µL, 0.44 mmol, 1.01 equiv.) and C_6D_6 (1 mL) were added to four vials, each containing different amounts of **1**: (5 mg, 0.01 mmol, 2.7 mol%), (11 mg, 0.023 mmol, 5.3 mol%), (20 mg, 0.044 mmol, 10.1 mol%) and (39 mg, 0.086 mmol, 19.6 mol%). The dark teal solutions were transferred to NMR tubes fused to Schlenk adapters and sealed under an SF_6 atmosphere as described above. The tubes were monitored by NMR spectroscopy over the course of one month. A yellow-brown film was observed to deposit on the walls of the glass over time.

Figure S17: Plots of catalyst degradation over time in the degradation of SF_6 catalyzed by **1** using $HSiEt_3$ as reducing agent at room temperature under 1 bar(a). Left: Stacked ^{1}H NMR spectra (499.9 MHz, C_6D_6), zoom of the meta proton signal in **1** after 1, 8 and 20 days with 2.7 mol%. Right: Plot of catalyst degradation over time for 2.7, 5.3, 10.1 and 19.6 mol% of **1** (right). The lines are only there to connect points and do not originate from a fitting.

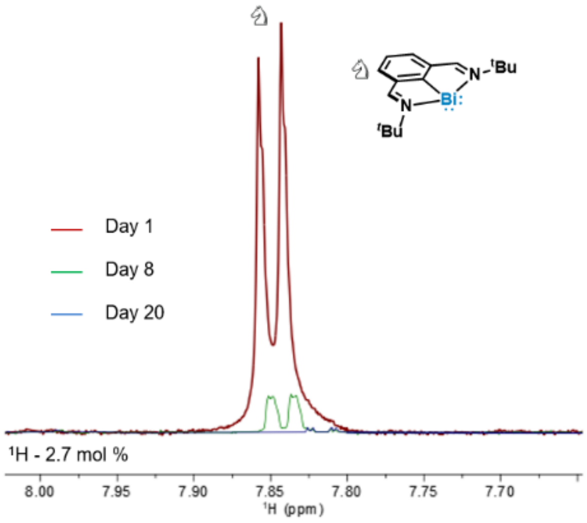

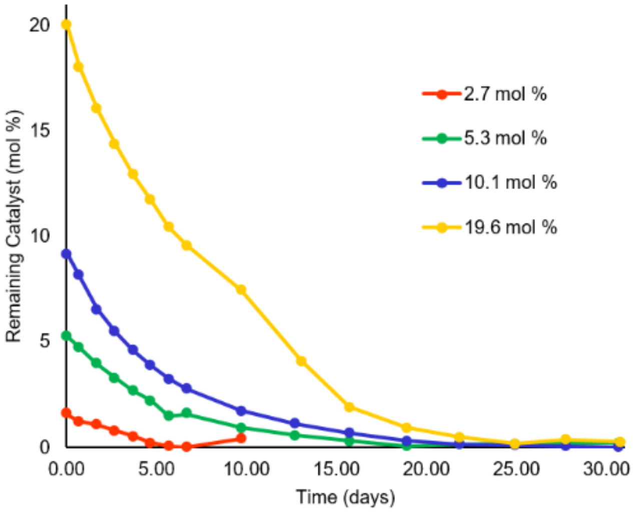

Figure S18: Conversion plot for the degradation of SF_6 catalyzed by different loadings of **1** using $HSiEt_3$ as reducing agent at room temperature under 1 bar(a). The lines are only there to connect points and do not originate from a fitting.

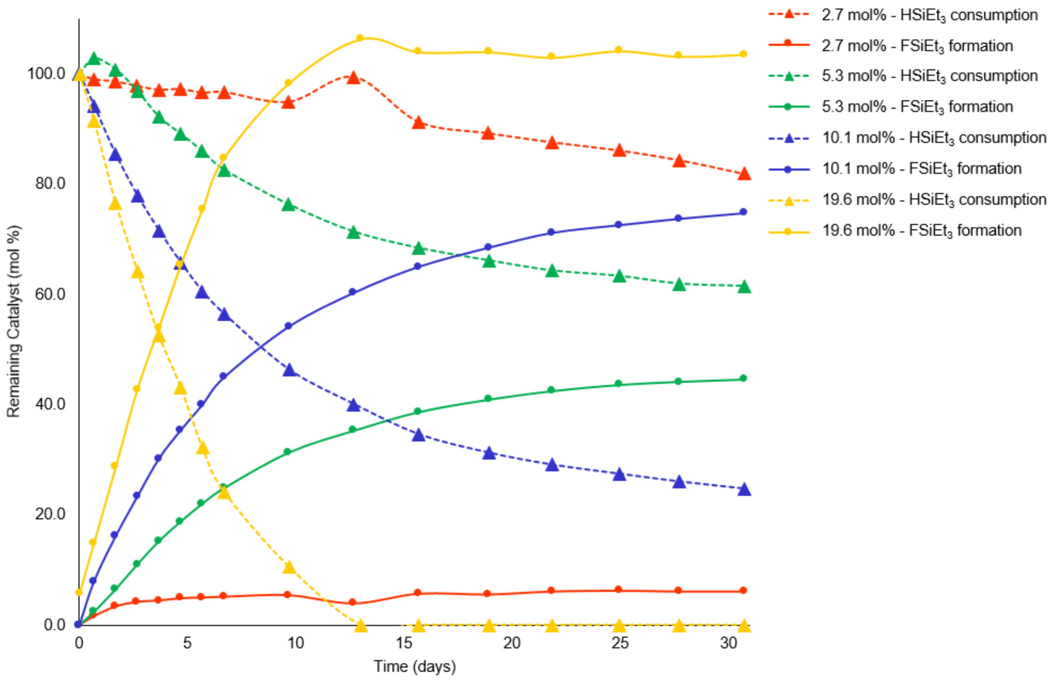

Scaled Reaction: A benzene solution (20 mL) of triethylsilane (1.64 mL, 10.3 mmol, 1.0 equiv.) was added to **1** (232 mg, 0.510 mmol, 5.0 mol%) in a 150 mL pressure Schlenk flask to give a dark teal solution. The flask was degassed, and pressurized with 1 bar(g) SF_6 and allowed to stand at room temperature for one month. A yellow-brown film was observed to deposit on the walls of the glass over time. After one month, the supernatant was removed and the solids dried *in vacuo*. The solid was analyzed by PXRD, SEM, EDX and XPS.

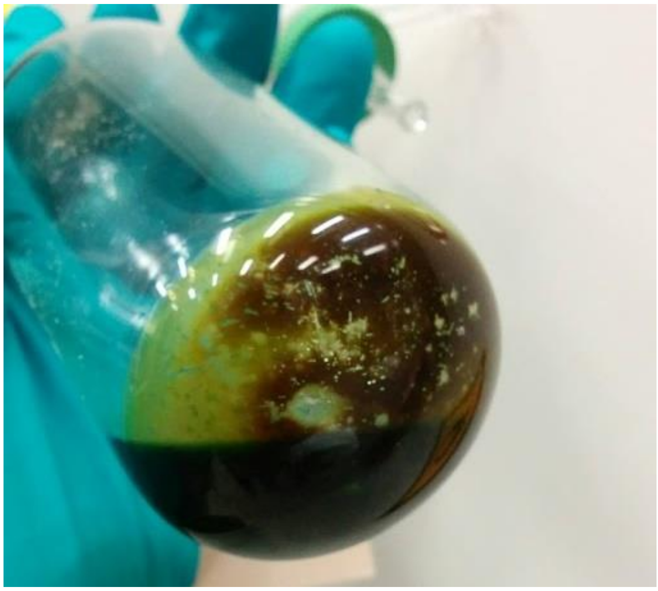

Figure S19: PXRD diffraction pattern for the yellow-brown precipitate isolated from the degradation of SF_6 using 5.0 mol% of **1** with $HSiEt_3$ as reducing agent. The sample is amorphous. Collected from 2–60° 2θ using a STOE Powder Diffraction System equipped with a 0.7092 Å Mo radiation source and a Mythen1K moving fixed Ω detector.

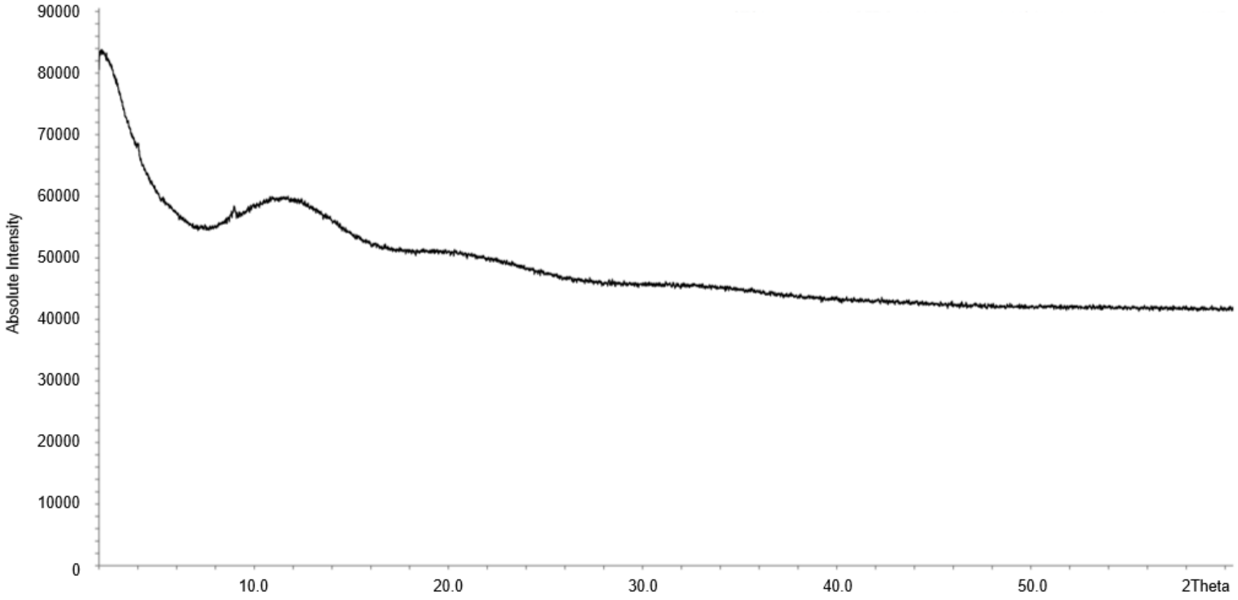

Figure S20: X-Ray photoelectron spectrum of the yellow-brown precipitate isolated from the degradation of SF_6 using 5.0 mol% of **1** with $HSiEt_3$ as reducing agent.

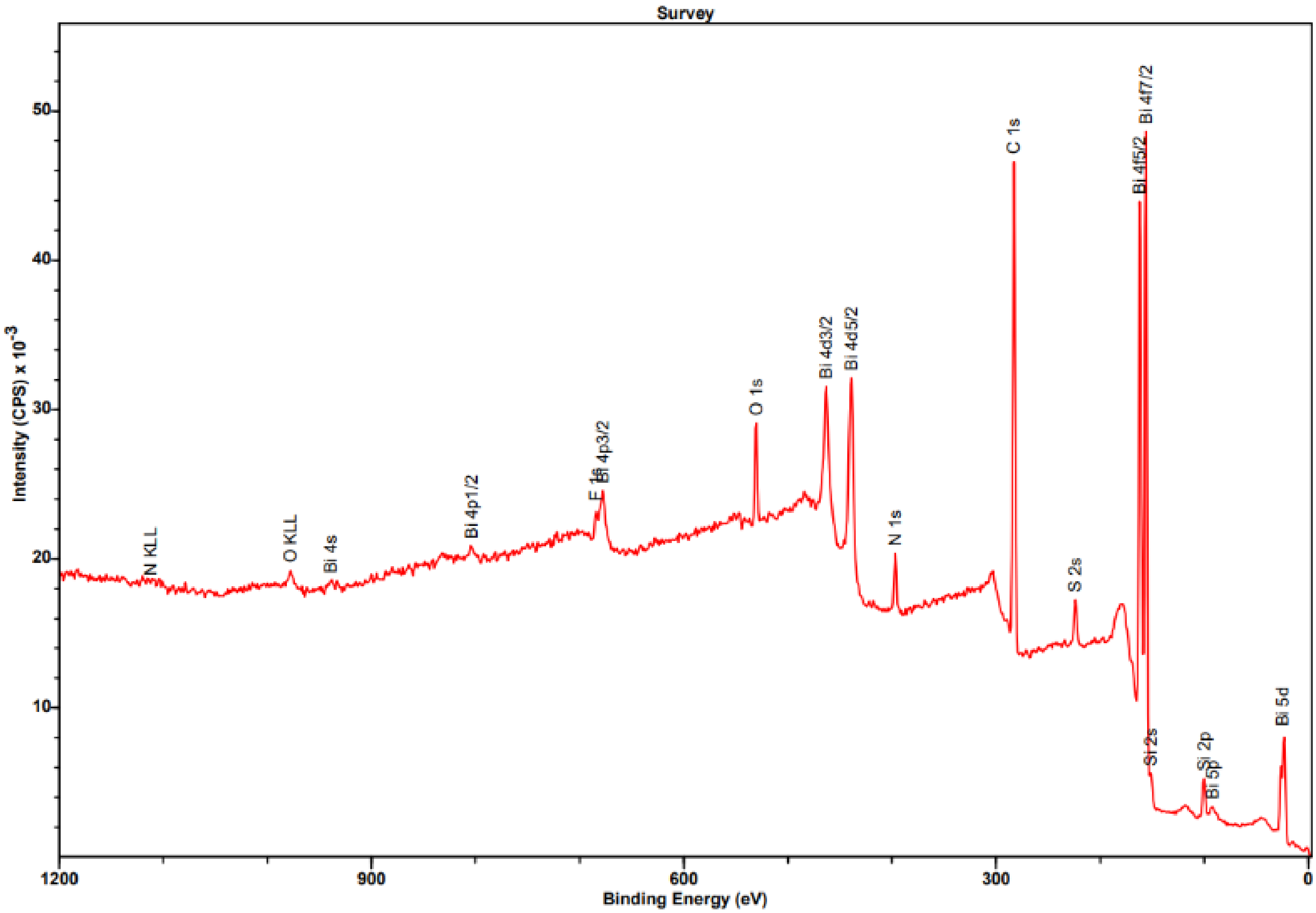

Figure S21: X-Ray photoelectron spectrum showing the Bi 4f and S 2p absorption edges of the yellow-brown precipitate isolated from the degradation of SF_6 using 5.0 mol% of **1** with $HSiEt_3$ as reducing agent.

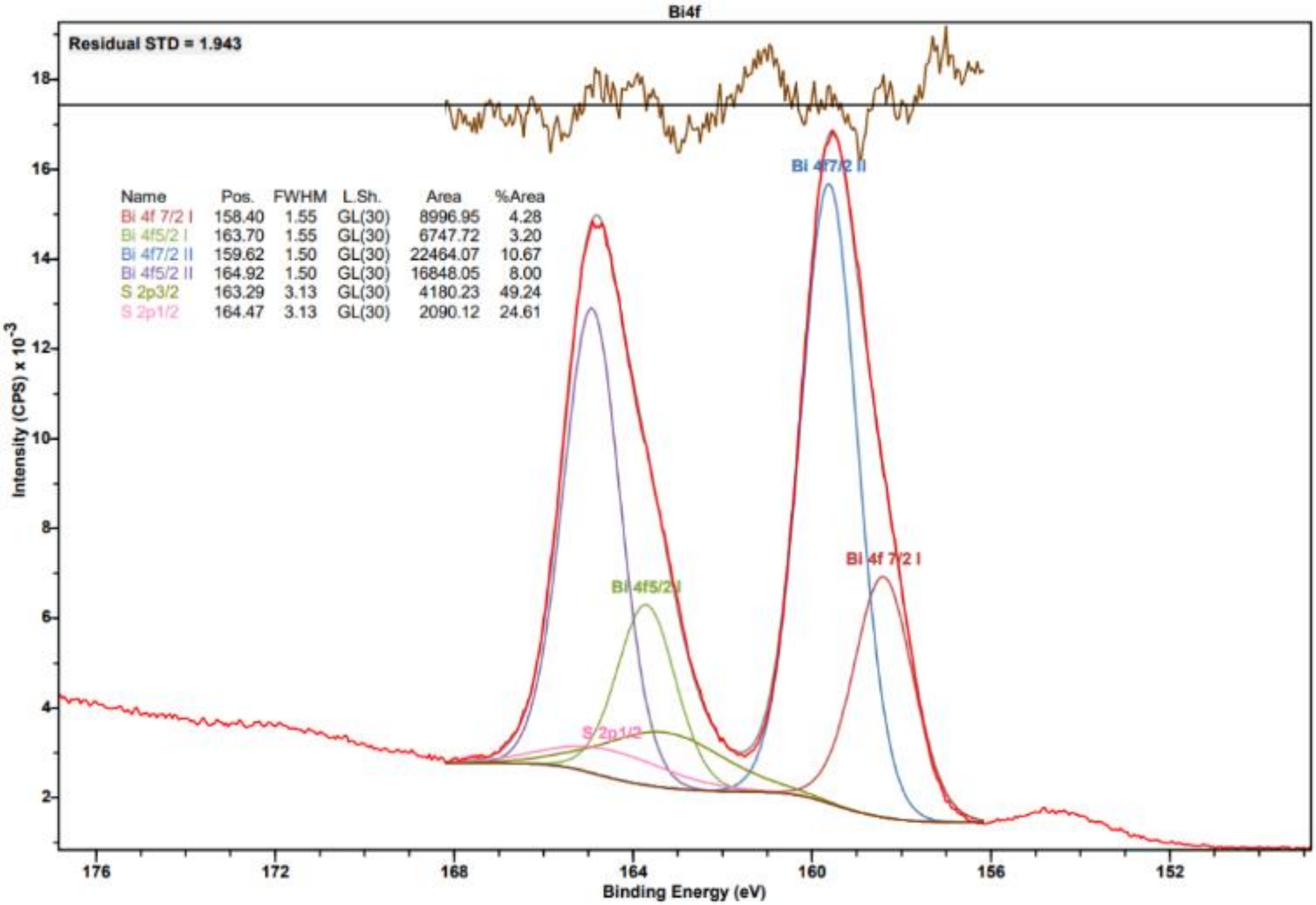

6.1.2 One Month Reaction Monitoring with PMe_3 as Reducing Agent

*1 bar(a) SF_6 with **1**:* A C_6D_6 stock solution (0.70 mL) containing PMe_3 (0.65 mmol, 0.93 M, 1.0 equiv.) and 4-fluoroanisole (0.66 mmol, 0.94 M, 1.0 equiv.) was added to solid **1** (6 mg, 0.01 mmol, 2.0 mol%) in a vial. The dark teal solution was transferred to an NMR tube fused to a Schlenk adapter and sealed under an SF_6 atmosphere as described above. The final NMR yield of $SPMe_3$ is 32%, which translates to a TON of 4.0 for SF_6.

*1 bar(a) SF_6 with **5**:* A C_6D_6 stock solution (0.70 mL) containing PMe_3 (0.65 mmol, 0.93 M, 1.0 equiv.) and 4-fluoroanisole (0.66 mmol, 0.94 M, 1.0 equiv.) was added to solid **5** (6 mg, 0.01 mmol, 2.1 mol%) in a vial. The dark blue solution was transferred to an NMR tube fused to a Schlenk adapter and sealed under an SF_6 atmosphere as described above. The final NMR yield of $SPMe_3$ is 41%, which translates to a TON of 5.0 for SF_6.

*1 bar(g) with **5**:* PMe_3 (47 mg, 0.62 mmol, 1.0 equiv.) and 4-fluoroanisole (84 mg, 0.67 mmol, 1.1 equiv.) were added to solid **5** (6 mg, 0.01 mmol, 2.3 mol%) in a vial, then dissolved in THF-d_8 (0.6 mL) and transferred to a J-Young tube. The dark blue solution was frozen, the argon atmosphere was removed, then the tube was pressurized with 1 bar(g) of SF_6. The final combined NMR yield of F_2PMe_3 and $SPMe_3$ is 70%, which translates to a TON of 8.0 for SF_6.

The above three reactions were monitored by ^1H, $^{19}F\{^1H\}$ and $^{31}P\{^1H\}$ NMR spectroscopy over the course of one month at room temperature. NMR yields of PMe_3 and $SPMe_3$ were determined by ^1H NMR (499.9 MHz) integrations relative to 4-fluoroanisole internal standard. NMR yields for SF_6 were determined by $^{19}F\{^1H\}$ NMR (470.3 MHz) integrations relative to 4-fluoroanisole internal standard. As SF_6 occupies the headspace of the tube as well as the solution, and the pulse sequence relaxation time was not optimized, the NMR yield of SF_6 is merely a qualitative metric. In the following plots this qualitative metric is used to demonstrate when the concentration of SF_6 in the tube is too low to be detected by NMR spectroscopy and to make an approximate correlation with the cessation of substrate conversion. Due to the 1:1 stoichiometry, the NMR yield of $SPMe_3$ can be used to approximate the turnover number for the decomposition of SF_6.

Figure S22: Conversion plot for degradation of SF_6 catalyzed by 2.0 mol% **1** using PMe_3 as reducing agent at room temperature over the course of one month in C_6D_6 in a sealed tube under 1 bar(a). The maximum turnover is 4.0, with SF_6 still observed by $^{19}F\{^1H\}$ NMR spectroscopy at the end of the month. The lines are only there to connect points and do not originate from a fitting.

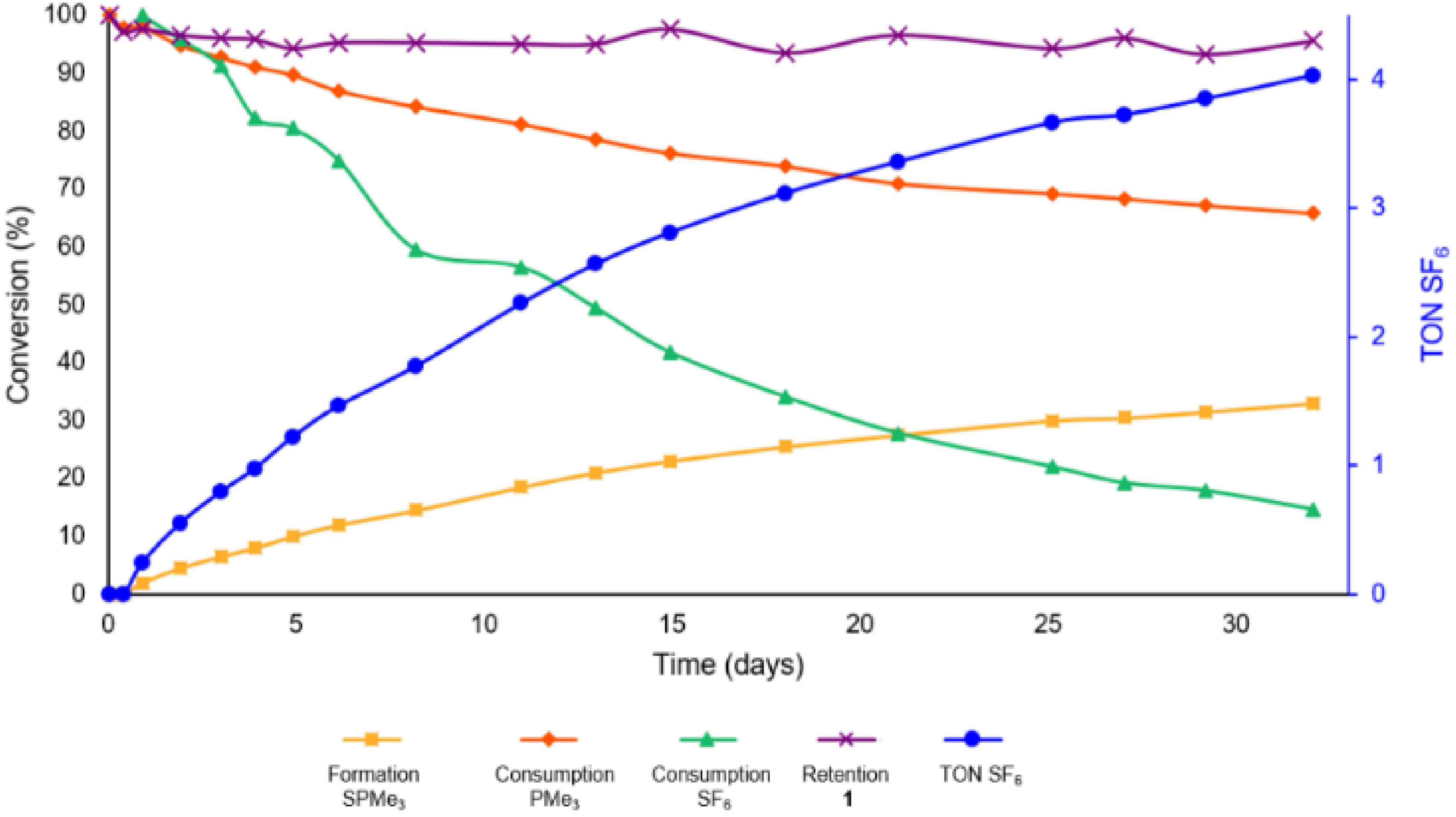

Figure S23: ^{1}H NMR spectrum (499.9 MHz, C_6D_6) zoom of aromatic region, on day 15 of monitoring the degradation of SF_6 catalyzed by 2.0 mol% **1** using PMe_3 as reducing agent at room temperature under 1 bar(a). Throughout the reaction, the only ligand bearing species observed by NMR is **1**, showing that to be the resting state.

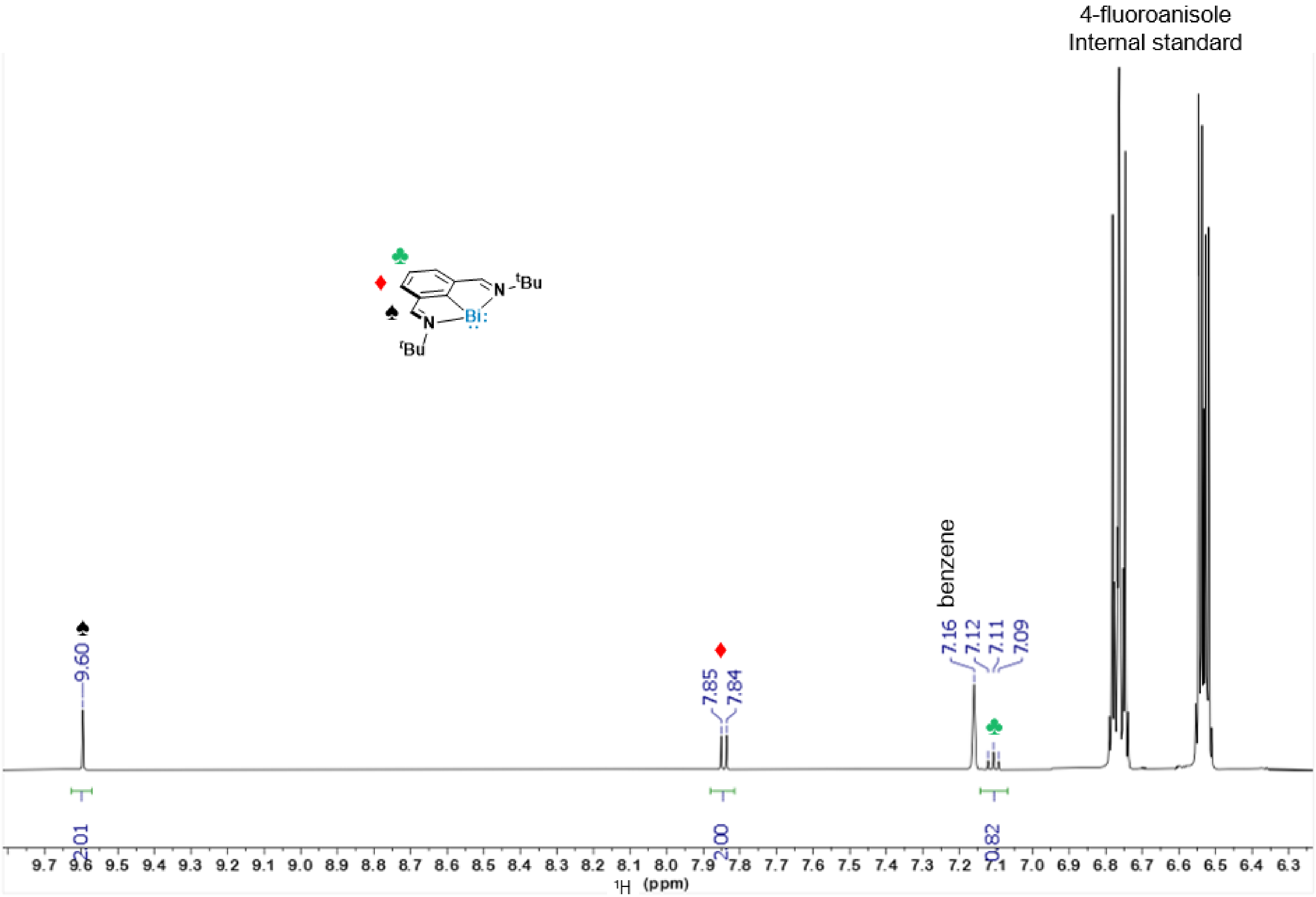

Figure S24: Conversion plot for degradation of SF_6 catalyzed by 2.1 mol% **5** using PMe_3 as reducing agent at room temperature over the course of one month in C_6D_6 in a sealed tube under 1 bar(a). The maximum turnover is 5.0. By day 11 SF_6 could no longer be detected by $^{19}F\{^1H\}$ NMR spectroscopy (marked with grey dashed line). The lines are only there to connect points and do not originate from a fitting.

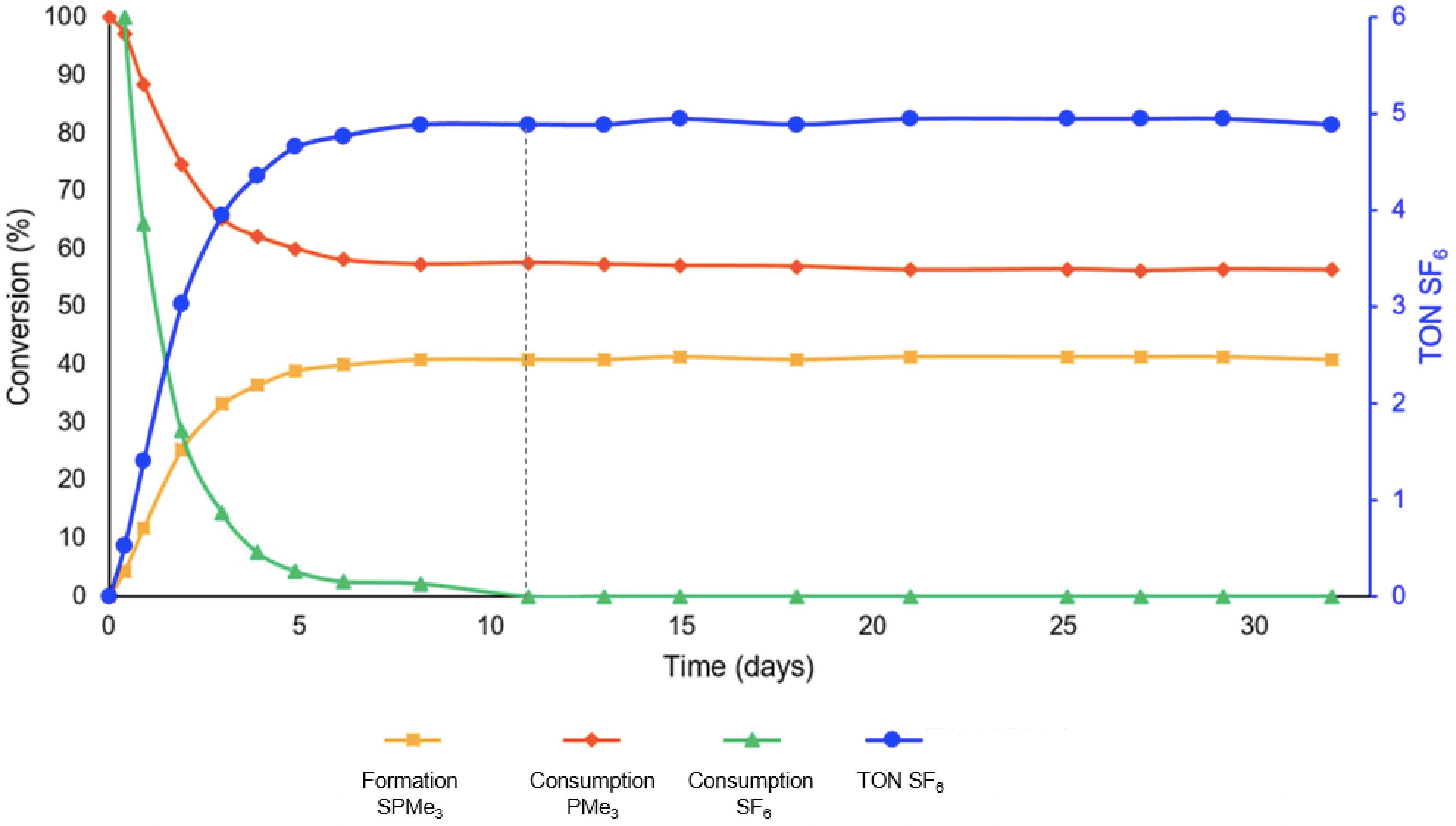

Figure S25: Stacked ^{1}H NMR spectra (499.9 MHz, C_6D_6) zoom of aromatic region, for the first 11 days of monitoring the degradation of SF_6 catalyzed by 2.1 mol% **5** using PMe_3 as reducing agent at room temperature under 1 bar(a). Throughout the reaction, the signals for **5** become broad and reappear as sharp signals once SF_6 is consumed. The catalyst aromatic signals do not completely match with those of pure **5**. This slight shifting could be due to interactions with the reaction components, such as PMe_3, or the internal standard, which could change the overall solvent properties. The amount of **5** recovered after 11 days was determined to be 43% by integrations relative to the internal standard.

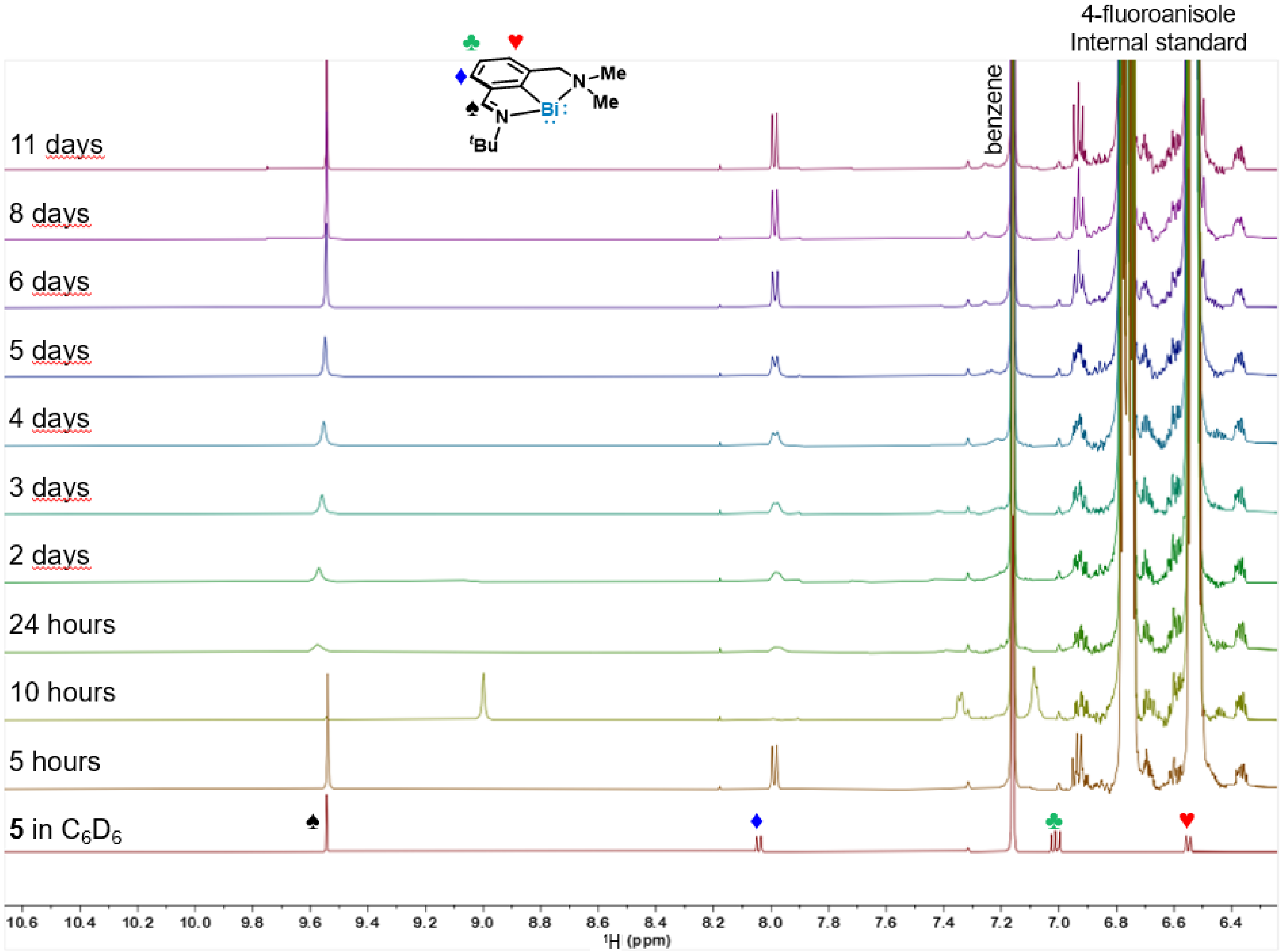

Figure S26: Conversion plot for degradation of SF_6 catalyzed by 2.3 mol% **5** using PMe_3 as reducing agent at room temperature over the course of one month in THF-*d*$_8$ in a J-Young tube at 1 bar(g). The maximum turnover is 8.0. By day 11, SF_6 could no longer be detected by $^{19}F\{^1H\}$ NMR spectroscopy (marked with grey dashed line). The lines are only there to connect points and do not originate from a fitting.

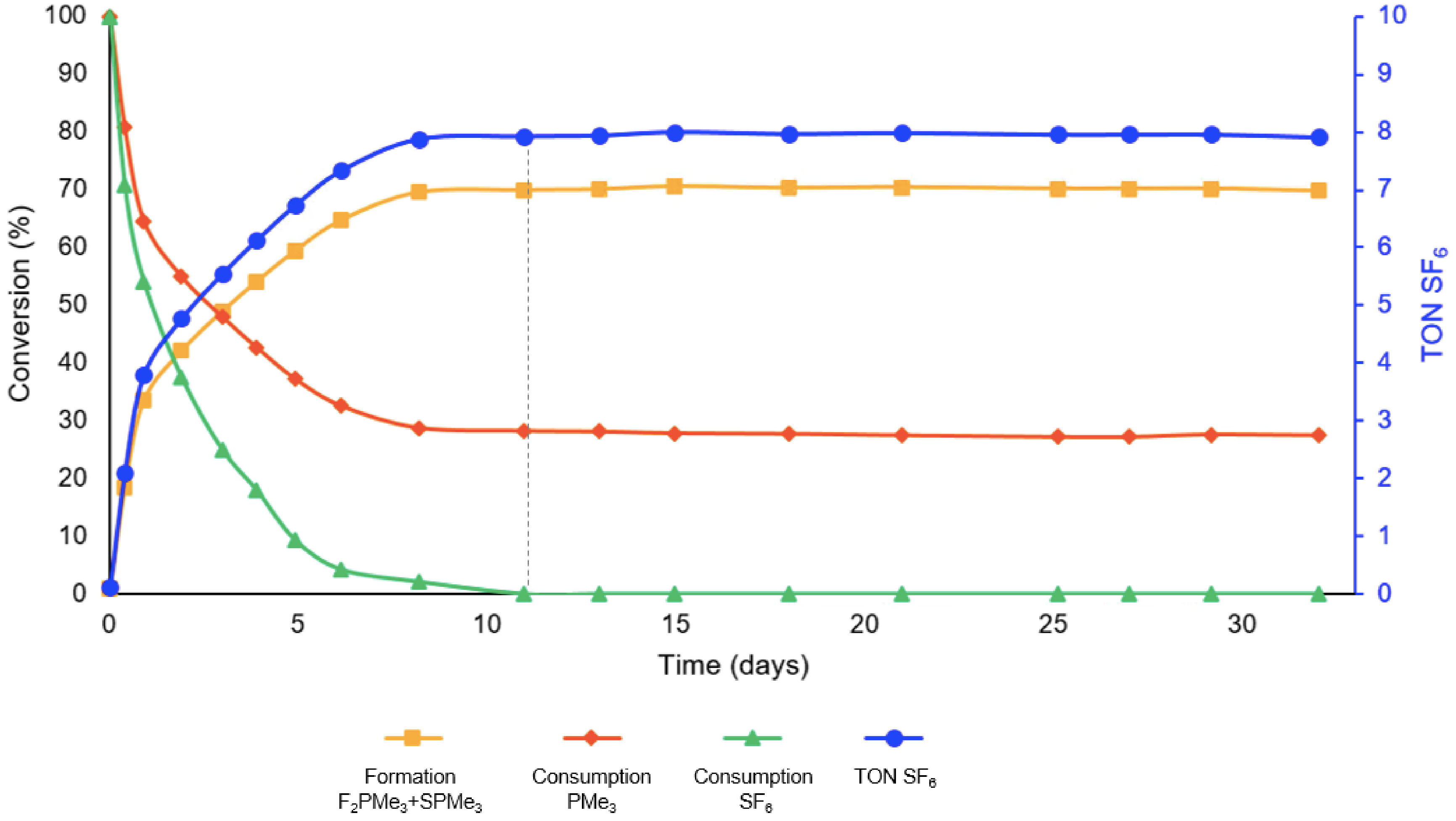

Figure S27: Stacked ^{1}H NMR spectra (499.9 MHz, THF-d_8) zoom of aromatic region, for the first 11 days of monitoring the degradation of SF_6 catalyzed by 2.3 mol% **5** using PMe_3 as reducing agent at room temperature and 1 bar(g). Throughout the reaction, the signals for **5** become broad and shift, then reappear as sharp signals once SF_6 is consumed. The amount of **5** recovered after 11 days was determined to be 37%.

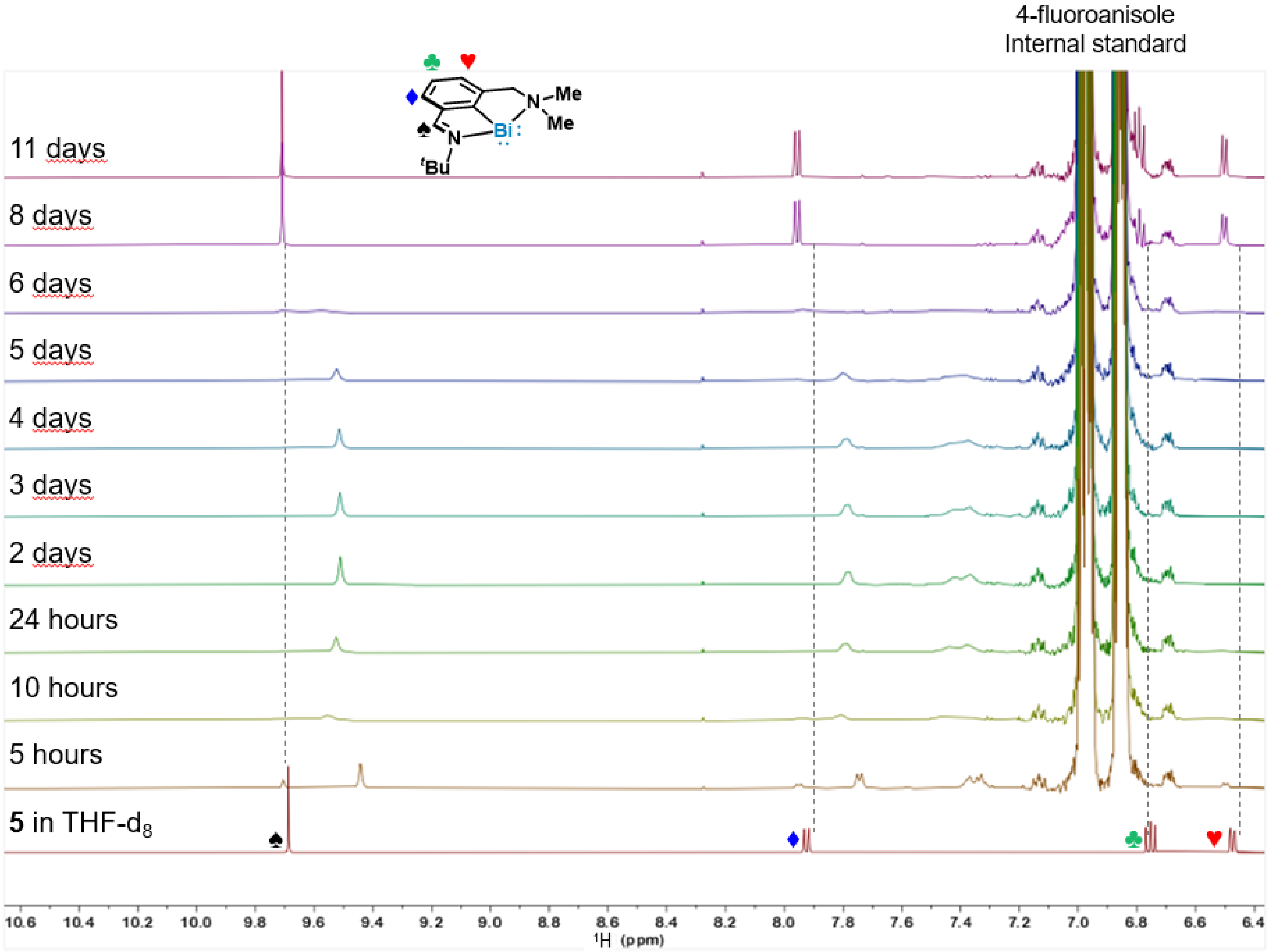

6.1.3 60 °C Reaction Monitoring

*1 bar(g) with **5**:* PMe_3 (53 mg, 0.70 mmol, 1.0 equiv.) and 4-fluoroanisole (82 mg, 0.65 mmol, 0.93 equiv.) were added to solid **5** (5 mg, 0.01 mmol, 1.6 mol%) in a vial, then dissolved in THF-d_8 (0.6 mL) and transferred to a J-Young tube. The dark blue solution was frozen, the argon atmosphere was removed, then the tube was pressurized with 1 bar(g) of SF_6 and inserted into a 500 MHz NMR spectrometer preheated to 60 °C. The tube was monitored by heteronuclear NMR spectroscopy at 60 °C every 10 min for 40 h. The final combined NMR yield of F_2PMe_3 and $SPMe_3$ is 98%, which translates to a TON of 15.5 for SF_6.

Figure S28: Conversion plot for degradation of SF_6 catalyzed by 1.6 mol% **5** using PMe_3 as reducing agent at 60 °C over the course of 40 h in THF-d_8 in a J-Young tube at 1 bar(g). The TON is 15.8. Using integrals from ^{1}H NMR spectra.

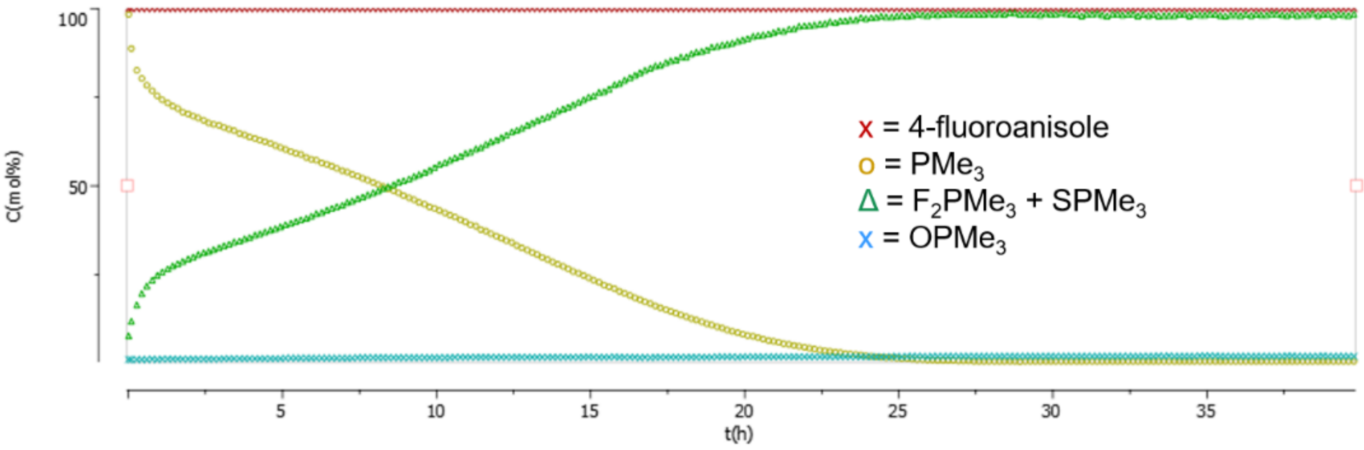

Figure S29: Stacked ^{1}H NMR spectra for degradation of SF_6 catalyzed by 1.6 mol% **5** using PMe_3 as reducing agent at 60 °C over the course of 40 h in THF-d_8 in a J-Young tube at 1 bar(g).

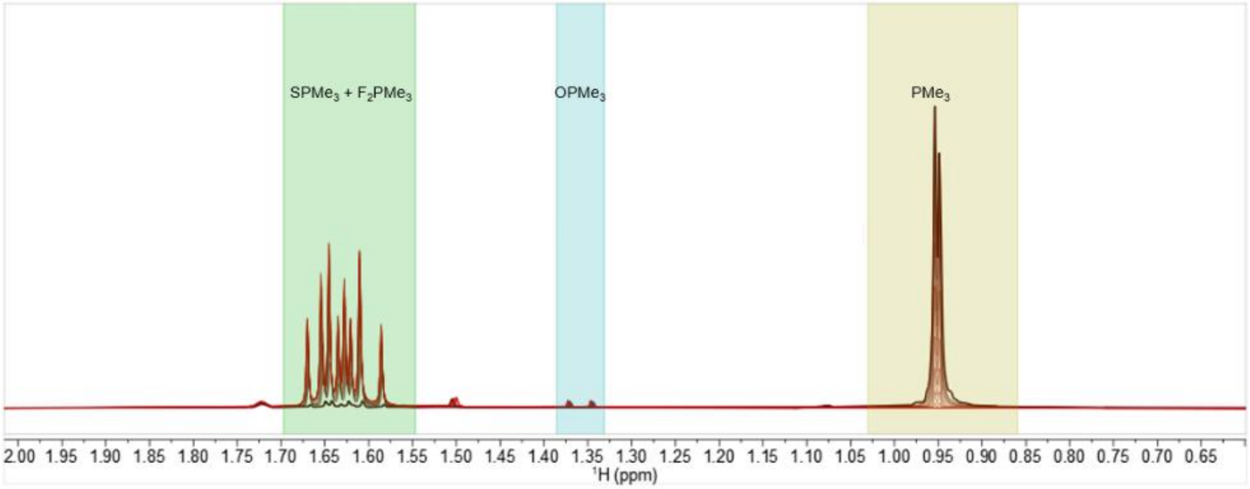

Figure S30: Conversion plot for degradation of SF_6 catalyzed by 1.6 mol% **5** using PMe_3 as reducing agent at 60 °C over the course of 40 h in THF-d_8 in a J-Young tube at 1 bar(g). Using integrals from $^{19}F\{^1H\}$ NMR spectra.

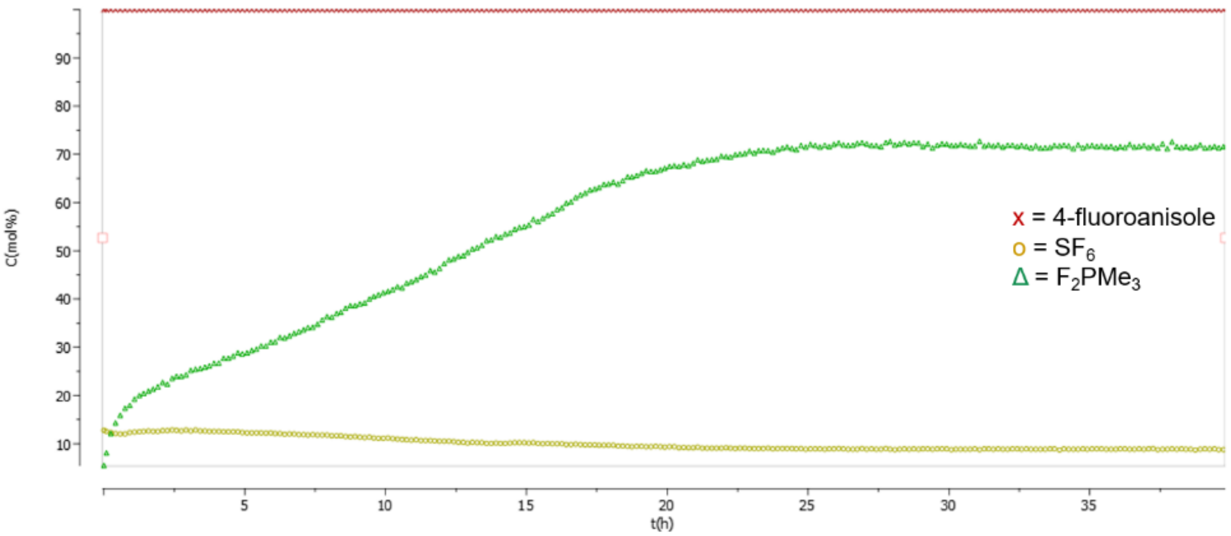

Figure S31: Stacked $^{19}F\{^{1}H\}$ NMR spectra for degradation of SF_6 catalyzed by 1.6 mol% **5** using PMe_3 as reducing agent at 60 °C over the course of 40 h in THF-*d*$_8$ in a J-Young tube at 1 bar(g).

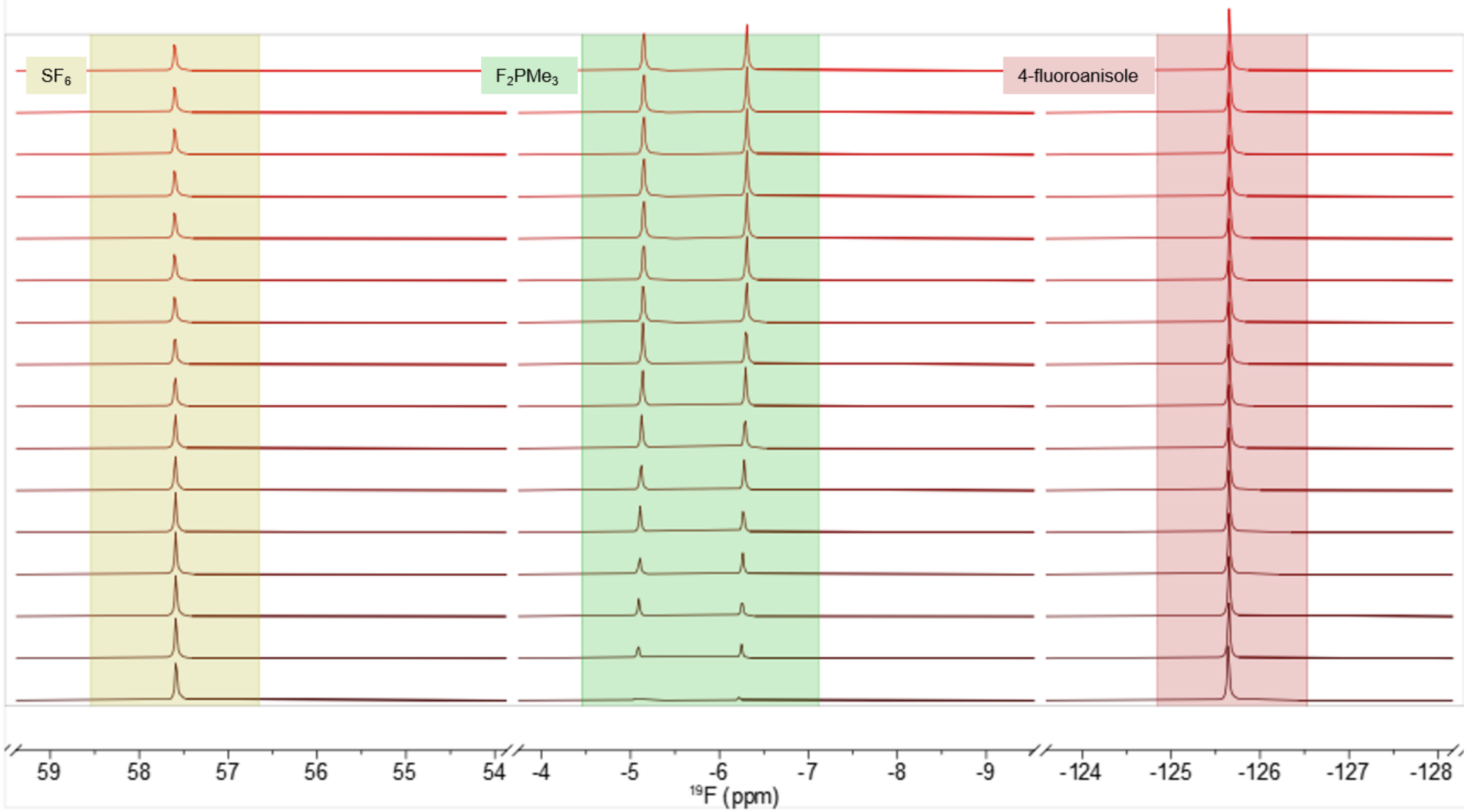

Figure S32: Stacked ^{1}H NMR spectra (499.9 MHz, THF-d_8) zoom of aromatic region, from different times of monitoring the degradation of SF_6 catalyzed by 1.6 mol% **5** using PMe_3 as reducing agent at 60 °C and 1 bar(g). Throughout the reaction, the signals for **5** are shifted and broadened compared to the pure compound.

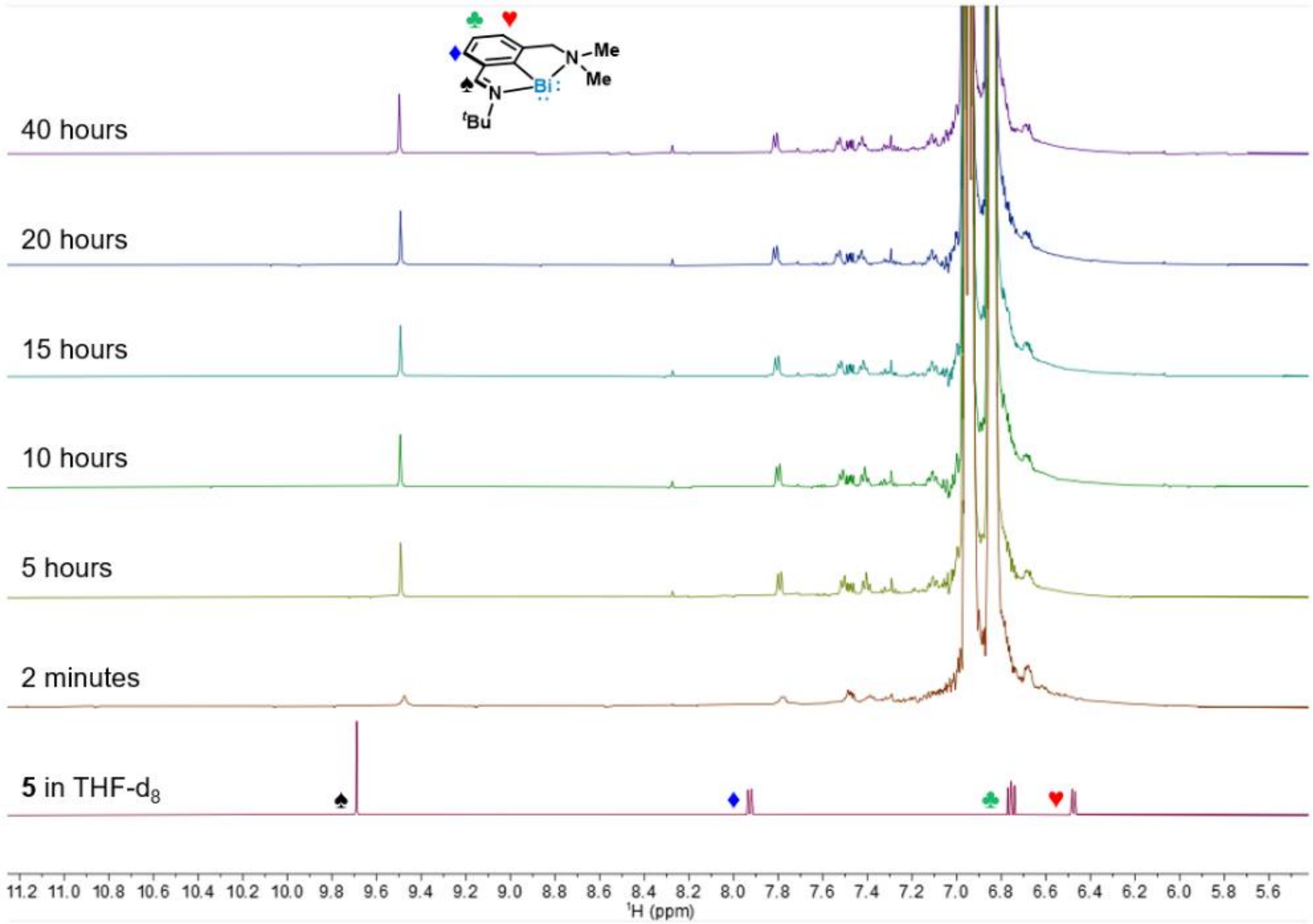

6.1.4 Solvent Optimization

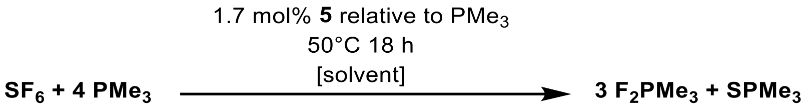

Without Additive: Compound **1** (5 mg, 0.01 mmol, 2.2 mol%) was weighed into four vials, then PMe_3 (67 µL, 41 mg, 0.54 mmol, 1.0 equiv.), 4-fluoroanisole (74 µL, 80 mg, 0.63 mmol, 1.2 equiv.) and solvent (1.2 mL) were added. The dark blue solutions were transferred to NMR tubes fused to Schlenk adapters and sealed under an SF_6 atmosphere as described above.

With Additive: Compound **1** (5 mg, 0.01 mmol, 2.2 mol%) was weighed into four vials, then $[NMe_4][F]$ (23 mg, 0.24 mmol, 0.46 equiv.) PMe_3 (67 µL, 41 mg, 0.54 mmol, 1.0 equiv.), 4-fluoroanisole (74 µL, 80 mg, 0.63 mmol, 1.2 equiv.) and solvent (1.2 mL) were added. The dark blue solutions were transferred to NMR tubes fused to Schlenk adapters and sealed under an SF_6 atmosphere as described above. Note that as no F_2PMe_3 was observed in the reaction mixtures, the NMR yield was determined for $SPMe_3$ only.

Table S1: Solvent optimization for the catalytic degradation of SF_6.

entry	solvent	additive	yield (F_2PMe_3+$SPMe_3$)	yield ($SPMe_3$)	TON (SF_6)
1	C_6D_6	-	-	62%	7.7
2	pyridine-d_5	-	3%	-	0.3
3	THF-d_8	-	30%	-	3.4
4	MeCN-d_3	-	52%	-	5.8
5	THF-d_8	$[NMe_4][F]$	-	4%	0.5
6	MeCN-d_3	$[NMe_4][F]$	-	8%	0.9

6.1.5 TON Optimization

Heated to 60 °C: PMe_3 (472 mg, 6.20 mmol, 1.0 equiv.) and 4-fluoroanisole (778 mg, 6.17 mmol, 1.0 equiv.) were added to solid **5** (5 mg, 0.01 mmol, 0.18 mol%) in a vial, then dissolved in THF-d_8 (4.0 mL) and transferred to a 150 mL pressure Schlenk flask. The dark blue solution was frozen, the argon atmosphere was removed, then the flask was pressurized with 1 bar(g) of SF_6. The flask was allowed to stand at 60 °C. After the first 18 h, the colour of the solution was a lighter blue than the initial colour and the colour gradually changed to yellow over the course of 5 days. An aliquot of the reaction mixture was taken after 11 days at 60 °C showing an NMR yield of 94.7% for the combined signals of F_2PMe_3 and $SPMe_3$, which translates to a TON of 133.4 for SF_6.

Heated to 60 °C: PMe_3 (0.812 g, 10.7 mmol, 1.0 equiv.) and 4-fluoroanisole (1.326 g, 10.52 mmol, 0.99 equiv.) were added to solid **5** (2 mg, 0.005 mmol, 0.05 mol%) in a vial, then dissolved in CD_3CN (6 mL) and transferred to a 150 mL pressure Schlenk flask. The dark blue solution was frozen, the argon atmosphere was removed, then the flask was pressurized with 1 bar(g) of SF_6. The flask was allowed to stand at 60 °C. After the first 18 h, the colour of the solution was a lighter blue than the initial colour and the solution gradually became colourless over the course of 3 days,

at which point an aliquot of the reaction mixture was taken to reveal an NMR yield of 97% for the combined signals of F_2PMe_3 and $SPMe_3$, which translates to a TON of 528.1 for SF_6.

6.1.6 SF_6 TON Calculation

In C_6D_6, the signal for $SPMe_3$ is resolved from all other signals and the NMR yield can be used to calculate the TON of SF_6 based on the 1:1 stoichiometry (Equations S1 and S3). The signals for $SPMe_3$ and F_2PMe_3 overlap in CD_3CN, THF-d_8 and pyridine-d_5. In this case, the NMR yield is determined for the combination of $SPMe_3$ and F_2PMe_3 relative to integrations of the internal standard, then the moles of $SPMe_3$ are determined by dividing the combined moles by 4 (based on reaction stoichiometry, Equation S2), then dividing this value by the moles of catalyst used (Equation S3).

Equation S1: Determination of moles $SPMe_3$ generated in a C_6D_6 reaction mixture. 4-fluoroanisole (CH_3, 3.25 ppm, s).

ES1.1 $$\text{expected } n_{SPMe_3} = \frac{n_{PMe_3}}{4}$$

ES1.2 Set 4-fluoroanisol (CH_3) integral = 3H

ES1.3 $$\text{NMR yield}_{SPMe_3} = \frac{SPMe_3 \text{ integral}}{2.25\text{H (ie. } {}^{1}/_{4}\text{ 9H)}} \times 100\%$$

ES1.4 $$n_{SPMe_3} = \frac{\text{NMR yield}_{SPMe_3}}{100\%}\ x \text{ expected } n_{SPMe_3}$$

Equation S2: Determination of moles $SPMe_3$ generated in a THF-d_8, CD_3CN, or pyridine-d_5 reaction mixture. 4-fluoroanisole (CH_3, 3.61, 3.76, 3.60 ppm, respectively, s).

ES2.1 $$\text{expected combined } n_{F_2PMe_3 + SPMe_3} = n_{PMe_3}$$

ES2.2 Set 4-fluoroanisol (CH_3) integral = 3H

ES2.3 $$\text{combined NMR yield}_{F_2PMe_3 + SPMe_3} = \frac{F_2PMe_3 + SPMe_3 \text{ combined integral}}{9H} \times 100\%$$

ES2.4 $$n_{F_2PMe_3 + SPMe_3} = \frac{\text{combined NMR yield}_{F_2PMe_3 + SPMe_3}}{100\%} \times \text{expected } n_{F_2PMe_3 + SPMe_3}$$

ES2.5 $$n_{SPMe_3} = \frac{n_{F_2PMe_3 + SPMe_3}}{4}$$

Equation S3: Determination of SF_6 TON.

$$TON_{SF_6} = \frac{n_{SPMe_3}}{n_{catalyst}}$$

Table S2: ^{1}H NMR chemical shifts and coupling constants for $SPMe_3$ and F_2PMe_3 in different solvents.

Solvent	*δ_H $SPMe_3$ (ppm)*	*$^2J_{HP}$ (Hz)*	*δ_H F_2PMe_3 (ppm)*	*$^2J_{HP}$ (Hz)*	*$^3J_{HF}$ (Hz)*
C_6D_6	1.14	15.0	1.45	20.0	10.0
CD_3CN	1.68	12.0	1.67	15.0	15.0
THF-d_8	1.64	15.0	1.62	18.0	12.0
pyridine-d_5	1.70	12.0	1.73	18.0	12.0

Figure S33: ^{1}H NMR spectra (499.9 MHz) of the reaction mixture for the degradation of SF_6 catalyzed by 2 mol% **5** using PMe_3 as reducing agent. Zoom of the methyl proton signals for $SPMe_3$ (♥) and F_2PMe_3 (♣) in C_6D_6 (left) and THF-d_8 (right). The ^{t}Bu (♠, 1.42 ppm, s) signal from **5** overlaps with the F_2PMe_3 signal in C_6D_6, whereas in THF-d_8 it is resolved from the relevant signals (^{t}Bu δ_H = 1.50 ppm).

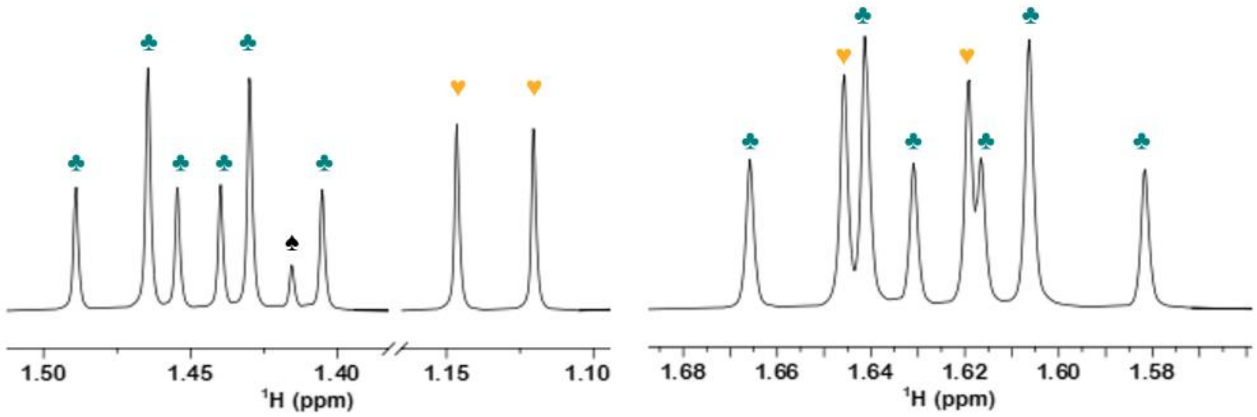

Figure S34: ^{1}H{^{19}F} NMR spectrum (499.9 MHz, THF-d_8) zoom of the $SPMe_3$ (♥) and F_2PMe_3 (♣) signals showing a 1:3 ratio.

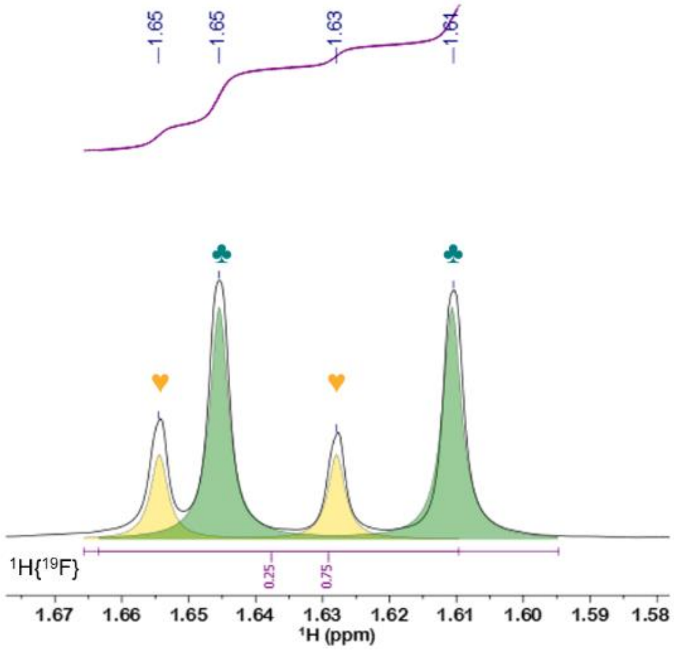

Figure S35: Stacked ^{1}H (top), $^{1}H\{^{31}P\}$ (middle) and $^{1}H\{^{19}F\}$ (bottom) NMR spectra (499.9 MHz, THF-d_8), zoom of the $SPMe_3$ (♥) and F_2PMe_3 (♣) signals.

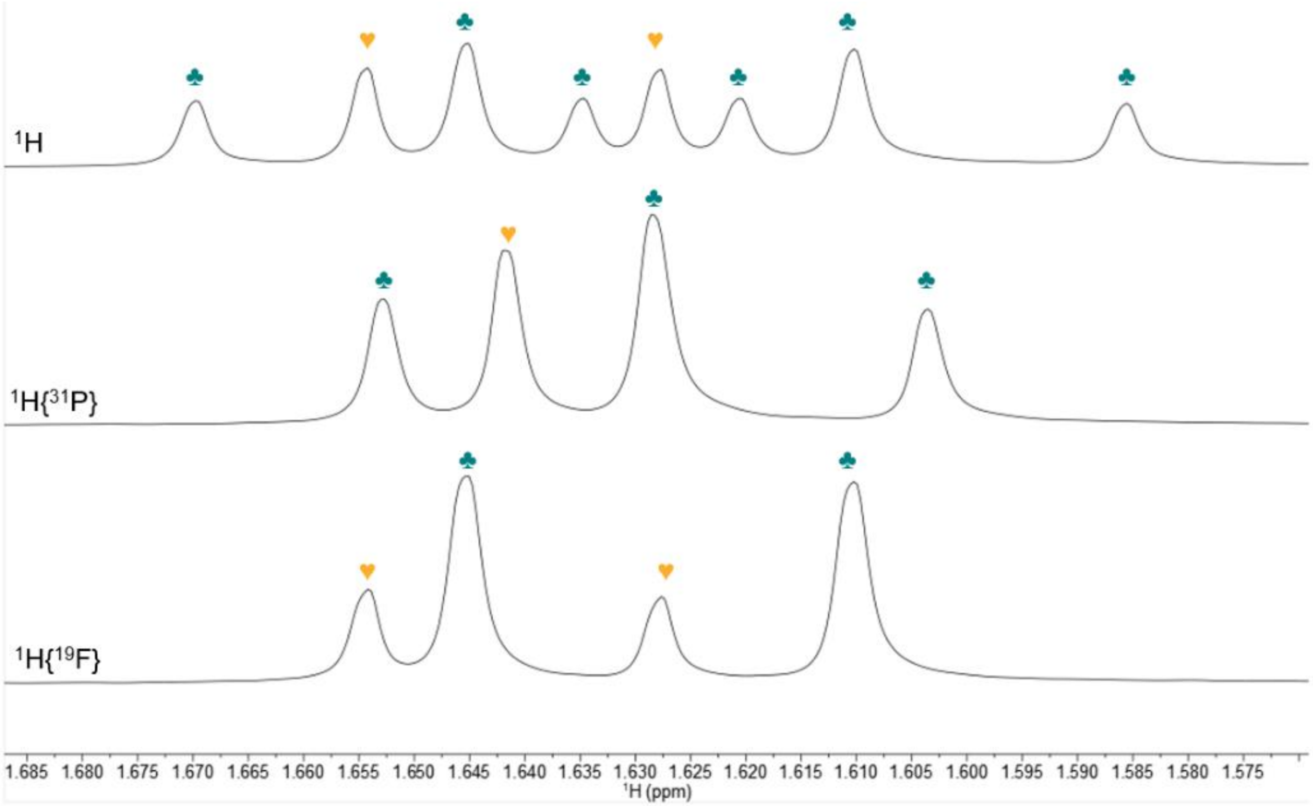

6.2 $PhSF_5$ Reactions

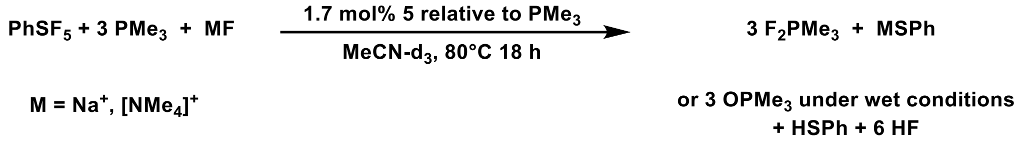

A CD_3CN stock solution (0.55 mL) containing **5** (0.0080 mmol, 0.014 M, 10.5 mol%), PMe_3 (0.27 mmol, 0.48 M, 2.7 equiv.), $PhSF_5$ (0.084 mmol, 0.15 M, 1.0 equiv.) and 1,3,5-trimethoxybenzene (0.25 mmol, 0.46 M, 3.0 equiv.) was transferred from a volumetric flask to an NMR tube containing solid electrolyte (0.09 mmol). The tubes were heated to 80 °C for 18 h.

Table S3: Electrolyte optimization for the catalytic degradation of $PhSF_5$.

entry	Electrolyte	yield (F_2PMe_3)	yield ($OPMe_3$)	recovered ($PhSF_5$)	yield (PhS^-)	yield (PhSH)	TON ($PhSF_5$)
1	-	14%	2%	81%	20%	-	2.2
2	$[NMe_4][F]$	-	29%	72%	-	30%	3.2
3	NaF	14%	1%	78%	21%	-	2.3

6.2.1 Postulated Catalytic Cycle for $PhSF_5$ degradation

Scheme S1: Postulated mechanism for the catalytic degradation of SF_6. Red: reduction; AT: Atom transfer; Ox: oxidation; Disprop: disproportionation.

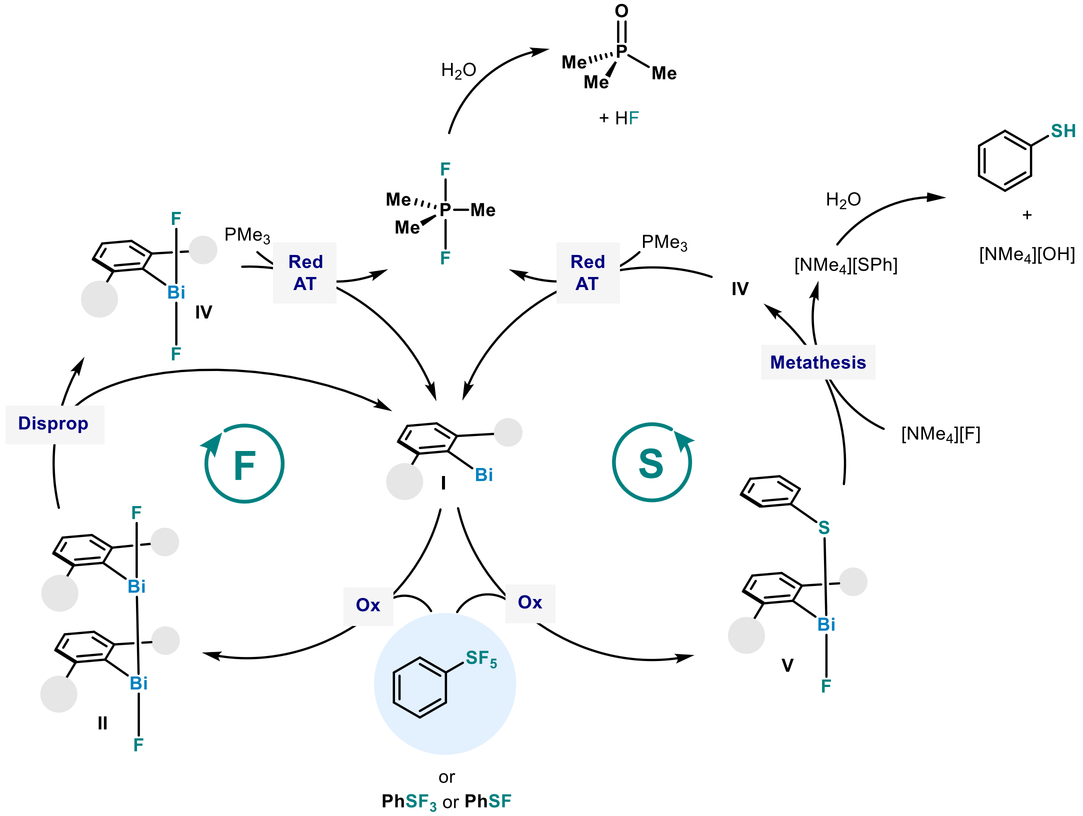

6.2.2 $PhSF_5$ TON Calculation

The TON of $PhSF_5$ is determined by the amount of sulfur-containing byproduct. In cases with no water present, fluorophosphorane and thiophenolate anion are released, in cases where water is present in the reaction mixture, fluorophosphorane is hydrolyzed to phosphine oxide and thiophenol is the sulfur containing byproduct.

Equation S4: Determination of recovered $PhSF_5$ and NMR yields for PhS^- and PhSH (PhX). 4-fluoroanisole (CH, 7.04 ppm, m, n = 2H), 1,3,5-trimethoxybenzene (CH, 6.10 ppm, s, n = 3H), $PhSF_5$ (CH, 7.83 ppm, m, m = 2H), PhS^- (CH, 7.06 ppm, br m, m = 4H), PhSH (CH, 7.14 ppm, m, m = 1H). PhX = SF_5, PhS^-, SH.

ES4.1 Set internal standard integral = Equivalents of internal standard relative to $PhSF_5$

ES4.2 $$\text{NMR yield}_{\text{PhX}} = \text{PhX(CH) integral} \times \frac{\text{nH internal standard signal}}{\text{mH PhX signal}} \times 100\%$$

Equation S5: Determination of NMR yield for F_2PMe_3 formed in a CD_3CN reaction mixture. 4-fluoroanisole (CH_3, 3.76 ppm, 3H, s), F_2PMe_3 (CH_3, 1.67 ppm, 9H, dt).

ES5.1 Set 4-fluoroanisol (CH_3) integral = 3H

ES5.2 $$\text{NMR yield}_{F_2PMe_3} = \frac{\text{integral } F_2PMe_3}{9\text{H}} \times 100\%$$

Equation S6: Determination of NMR yield for $OPMe_3$ formed in a CD_3CN reaction mixture. 4-fluoroanisole (CH_3, 3.76 ppm, 3H, s), $OPMe_3$ (CH_3, 1.40 ppm, 9H, d).

ES6.1 Set 4-fluoroanisol (CH_3) integral = 3H

ES6.2 $$\text{NMR yield}_{OPMe_3} = \frac{\text{integral } OPMe_3}{9\text{H}} \times 100\%$$

Equation S7: Determination of $PhSF_5$ TON. PhX = PhSH, or PhS^-.

ES7.1 $$n_{\text{PhX}} = \frac{\text{NMR yield PhX}}{100\%} \times n_{\text{PhSF}_5}$$

ES7.2 $$\text{TON}_{\text{PhSF}_5} = \frac{n_{\text{PhX}}}{n_{\text{catalyst}}}$$

6.3 Control Experiments

All control experiments were conducted at 80 °C for 18 h. Control reactions using SF_6 were flame sealed in an NMR tube as described above.

Table S4: Control experiment results. n.d. = not detected.

entry	[catalyst]	solvent	reducing agent (equiv.)	susbtrate (equiv.)	electrolyte (equiv.)	yield ($FSiEt_3$)	yield (F_2PMe_3)	recovered ($PhSF_5$)
1	-	C_6D_6	$HSiEt_3$ (1.0)	SF_6 (excess)	-	n.d.	-	-
2	-	C_6D_6	$HSiEt_3$ (1.0)	4-fluoroanisole (1.0)	-	n.d.	-	-
3	-	C_6D_6	PMe_3 (1.0)	SF_6 (excess)	-	-	n.d.	-
4	**1** (1.7 mol%)	C_6D_6	PMe_3 (1.0)	4-fluoroanisole (1.0)	-	-	n.d.	-
5	**5** (1.7 mol%)	C_6D_6	PMe_3 (1.0)	4-fluoroanisole (1.0)	-	-	n.d.	-
6	-	C_6D_6	PMe_3 (1.0)	4-fluoroanisole (1.0)	-	-	n.d.	-
7	-	CD_3CN	PMe_3 (1.0)	$PhSF_5$ (1.0)	-	-	n.d.	97%

7. X-Ray and TEM

The Bi atoms in **2**, **3** and **4** reside in the plane formed by N(1), C(1) and N(2) of the ligand. The imine C–N bond lengths in **2**, **3** and **4** range between 1.268-1.282 Å, indicating preservation of the double bond, and that the pincer ligand remains monoanionic.

7.1 Single crystal structure analysis of 2 MeCN solvate

$C_{36}H_{49}Bi_2F_6N_5O_6S_2$, M_r = 1243.88 g mol^{-1}, orange block, crystal size 0.169 x 0.111 x 0.10 mm^3, tetragonal, space group *P*4/*ncc* [130], a = 20.7998(11) Å, c = 20.6559(16) Å, V = 8936.4(12) Å^3, T = 100(2) K, Z = 8, D_{calc} = 1.849 g·cm^3, λ = 0.71073 Å, $\mu(Mo\text{-}K_\alpha)$ = 8.031 mm^{-1}, Gaussian correction ($T_{\min}$ = 0.45735, $T_{\max}$ = 0.61965), Bruker-AXS Kappa Mach3 with APEX-II detector and IµS micro focus Mo-anode X-ray source, 1.958 < θ < 35.630°, 646462 measured reflections, 10313 independent reflections, 8762 reflections with $I > 2\sigma(I)$, R_{int} = 0.0461. The structure was solved by *SHELXT* and refined by full-matrix least-squares (*SHELXL*) against F^2 to R_1 = 0.0196 [$I > 2\sigma(I)$], wR_2 = 0.0445 [all data], 335 parameters and 80 restraints.

Figure S36: The solid state structure of **2** MeCN solvate. H atoms have been removed for clarity and disordered regions shown in grey.

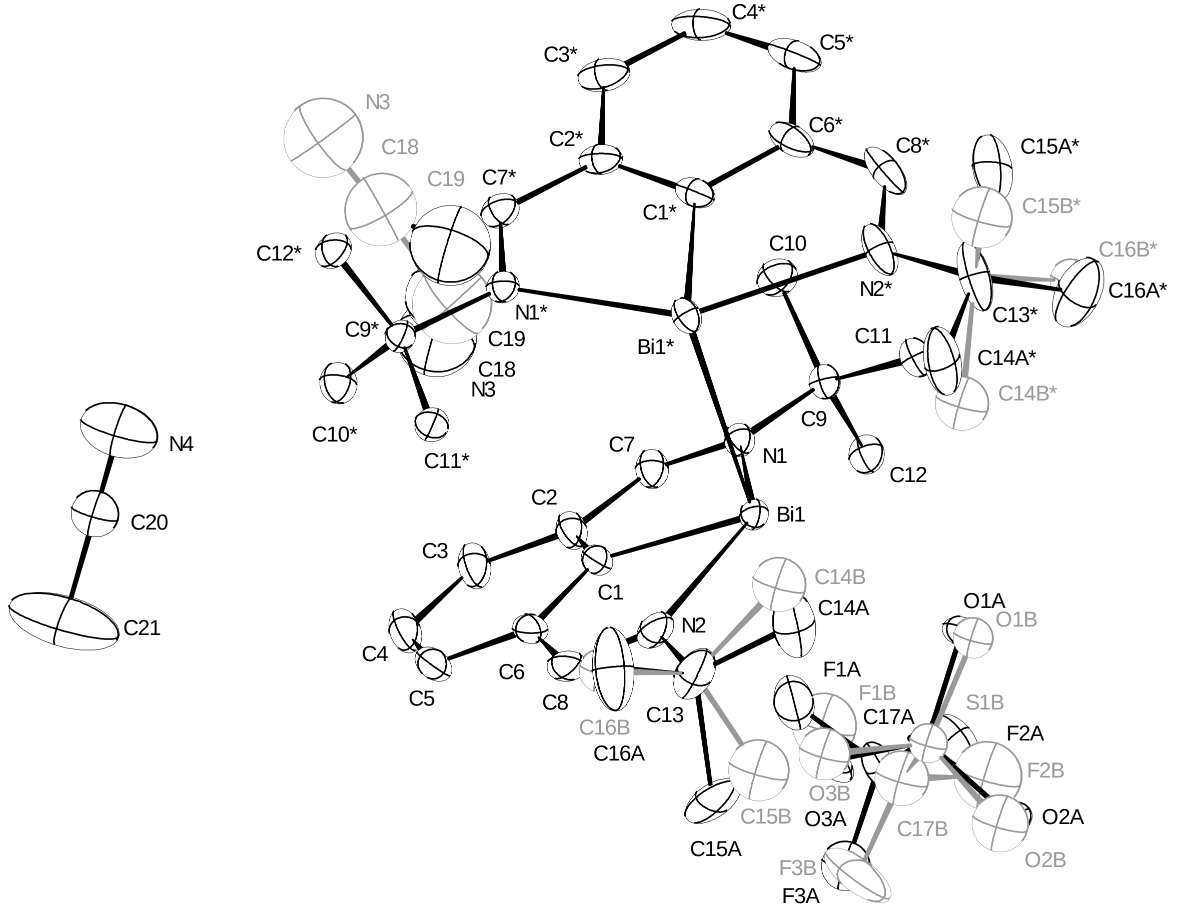

Figure S37: Crystal faces and unit cell determination/refinement of **2** MeCN solvate.

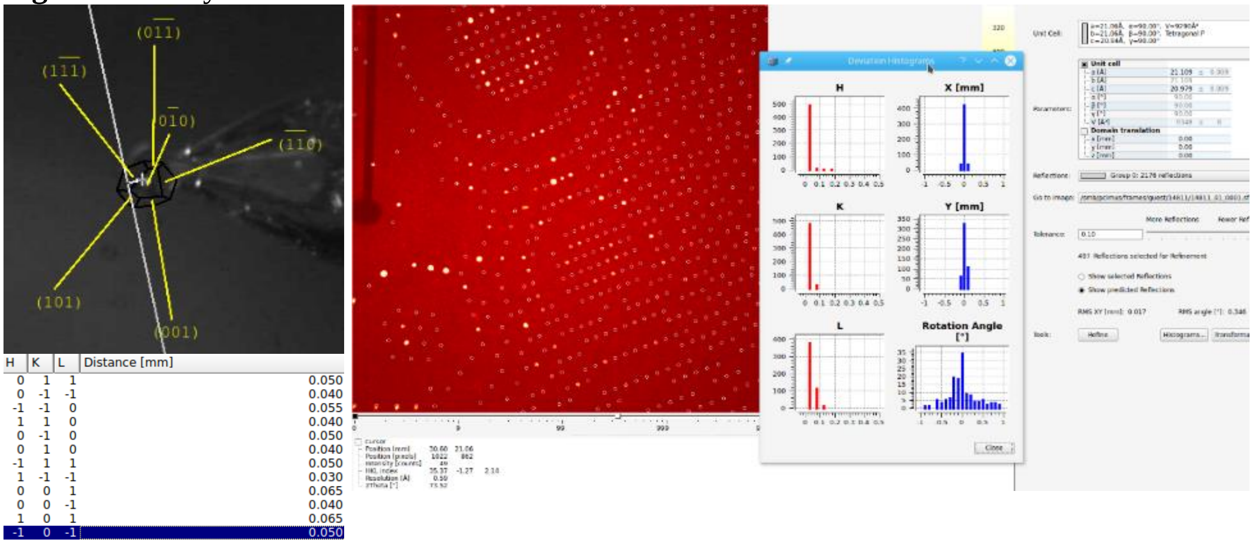

INTENSITY STATISTICS FOR DATASET

Resolution	#Data	#Theory	%Complete	Redundancy	Mean I	Mean I/s	Rmerge	Rsigma
Inf - 2.63	187	189	98.9	71.02	35.07	162.42	0.0223	0.0043
2.63 - 1.69	439	439	100.0	95.49	27.65	177.99	0.0222	0.0041
1.69 - 1.32	616	616	100.0	102.32	17.90	166.29	0.0239	0.0039
1.32 - 1.14	637	637	100.0	100.99	12.75	138.11	0.0300	0.0045
1.14 - 1.03	630	630	100.0	85.80	9.88	112.40	0.0366	0.0055
1.03 - 0.95	646	646	100.0	71.40	8.57	88.64	0.0424	0.0067
0.95 - 0.89	647	647	100.0	62.45	6.72	71.18	0.0518	0.0086
0.89 - 0.85	550	550	100.0	58.58	5.60	62.40	0.0609	0.0103
0.85 - 0.81	640	640	100.0	56.04	5.03	55.40	0.0690	0.0118
0.81 - 0.78	573	573	100.0	54.10	4.11	46.45	0.0822	0.0148
0.78 - 0.75	654	654	100.0	51.98	3.82	41.24	0.0886	0.0164
0.75 - 0.72	790	790	100.0	49.80	3.50	38.08	0.1004	0.0190
0.72 - 0.70	591	591	100.0	47.80	3.10	33.05	0.1107	0.0221
0.70 - 0.68	671	671	100.0	46.16	2.55	27.45	0.1333	0.0275
0.68 - 0.66	758	758	100.0	44.59	2.39	25.27	0.1443	0.0309
0.66 - 0.65	389	389	100.0	43.69	1.93	21.01	0.1751	0.0385
0.65 - 0.64	443	443	100.0	42.16	1.85	18.95	0.1876	0.0420
0.64 - 0.62	951	951	100.0	40.84	1.86	18.60	0.1904	0.0438
0.62 - 0.61	512	512	100.0	39.73	1.58	16.05	0.2201	0.0524
0.61 - 0.60	567	567	100.0	38.09	1.49	14.80	0.2332	0.0593
0.60 - 0.59	432	471	91.7	16.41	1.35	8.33	0.2644	0.1180
0.69 - 0.59	4399	4438	99.1	39.19	1.87	18.78	0.1810	0.0466
Inf - 0.59	12323	12364	99.7	57.66	6.40	59.89	0.0459	0.0116

Complete .cif-data of the compound are available under the CCDC number CCDC-**2351153**.

A resolution cut-off (SHEL 999 0.61) was applied to the data set to exclude the poorly determined reflections at high diffraction angles. Two reflections were omitted from the data set due to high I/σI before the final refinement cycles (OMIT 1 1 0; OMIT 1 1 2). The structure contains a disordered *tert.*-butyl group, a disordered trifluoromethanesulfonate anion and two disordered

MeCN solute molecules. The molecule of **2** is located on a twofold rotational axis, creating the complete molecule from the asymmetric unit. For the refinement of the disordered *tert.*-Butyl group, it was split into two parts and fixed occupancy 60:40%. The MeCN solute molecules are in special positions (double and quadruple rotational axis). The disordered trifluoromethanesulfonate and MeCN solute molecules were described using the DSR tool plug-in in Olex2.[13,14] For the trifluoromethanesulfonate, fixed occupancies of 70:30% were used for the final refinement cycles. The minor parts were described by isotropic displacement parameters.

Table S5: Crystal data and structure refinement of **2** MeCN solvate.

Identification code	14811	
Empirical formula	$C_{36} H_{49} Bi_2 F_6 N_5 O_6 S_2$	
Color	orange	
Formula weight	1243.88 g·mol^{-1}	
Temperature	100(2) K	
Wavelength	0.71073 Å	
Crystal system	Tetragonal	
Space group	*P*4/*ncc*, (no. 130)	
Unit cell dimensions	a = 20.7998(11) Å	α= 90°.
	b = 20.7998(11) Å	β= 90°.
	c = 20.6559(16) Å	γ = 90°.
Volume	8936.4(12) Å^3	
Z	8	
Density (calculated)	1.849 Mg·m^{-3}	
Absorption coefficient	8.031 mm^{-1}	
F(000)	4800 e	
Crystal size	0.169 x 0.111 x 0.10 mm^3	
θ range for data collection	1.958 to 35.630°.	
Index ranges	$-34 \le h \le 34$, $-34 \le k \le 34$, $-33 \le l \le 32$	
Reflections collected	646462	
Independent reflections	10313 [R_{int} = 0.0461]	
Reflections with I>2σ(I)	8762	
Completeness to θ = 25.242°	100.0%	
Absorption correction	Gaussian	
Max. and min. transmission	0.61965 and 0.45735	
Refinement method	Full-matrix least-squares on F^2	
Data / restraints / parameters	10313 / 80 / 335	
Goodness-of-fit on F^2	1.124	
Final R indices [I>2σ(I)]	R_1 = 0.0196	wR^2 = 0.0406
R indices (all data)	R_1 = 0.0291	wR^2 = 0.0445
Extinction coefficient	n/a	
Largest diff. peak and hole	1.121 and -0.805 e·Å^{-3}	

Table S6: Bond lengths [Å] and angles [°] of 2 MeCN solvate.

Bi(1)-Bi(1)#1	3.09439(19)	Bi(1)-N(1)	2.4698(14)
Bi(1)-N(2)	2.5438(16)	Bi(1)-C(1)	2.1879(17)
N(1)-C(7)	1.281(2)	N(1)-C(9)	1.493(2)
N(2)-C(8)	1.279(3)	N(2)-C(13)	1.490(3)
C(1)-C(2)	1.385(3)	C(1)-C(6)	1.390(2)
C(2)-C(3)	1.403(3)	C(2)-C(7)	1.462(3)
C(3)-H(3)	0.9500	C(3)-C(4)	1.389(3)
C(4)-H(4)	0.9500	C(4)-C(5)	1.387(4)
C(5)-H(5)	0.9500	C(5)-C(6)	1.401(3)
C(6)-C(8)	1.453(3)	C(7)-H(7)	0.9500
C(8)-H(8)	0.9500	C(9)-C(10)	1.526(2)
C(9)-C(11)	1.525(2)	C(9)-C(12)	1.531(2)
C(10)-H(10A)	0.9800	C(10)-H(10B)	0.9800
C(10)-H(10C)	0.9800	C(11)-H(11A)	0.9800
C(11)-H(11B)	0.9800	C(11)-H(11C)	0.9800
C(12)-H(12A)	0.9800	C(12)-H(12B)	0.9800
C(12)-H(12C)	0.9800	C(13)-C(14A)	1.400(5)
C(13)-C(15A)	1.624(5)	C(13)-C(16A)	1.509(10)
C(13)-C(14B)	1.659(9)	C(13)-C(15B)	1.446(10)
C(13)-C(16B)	1.509(15)	C(14A)-H(14A)	0.9800
C(14A)-H(14B)	0.9800	C(14A)-H(14C)	0.9800
C(15A)-H(15A)	0.9800	C(15A)-H(15B)	0.9800
C(15A)-H(15C)	0.9800	C(16A)-H(16A)	0.9800
C(16A)-H(16B)	0.9800	C(16A)-H(16C)	0.9800
C(14B)-H(14D)	0.9800	C(14B)-H(14E)	0.9800
C(14B)-H(14F)	0.9800	C(15B)-H(15D)	0.9800
C(15B)-H(15E)	0.9800	C(15B)-H(15F)	0.9800
C(16B)-H(16D)	0.9800	C(16B)-H(16E)	0.9800
C(16B)-H(16F)	0.9800	N(4)-C(20)	1.100(7)
C(20)-C(21)	1.408(10)	C(21)-H(21A)	1.0065
C(21)-H(21C)	1.0065	C(21)-H(21B)	1.0065
S(1A)-O(1A)	1.447(3)	S(1A)-O(2A)	1.447(2)
S(1A)-O(3A)	1.455(4)	S(1A)-C(17A)	1.800(4)
F(1A)-C(17A)	1.338(5)	F(2A)-C(17A)	1.318(4)

F(3A)-C(17A)	1.386(6)	S(1B)-O(1B)	1.423(8)
S(1B)-O(2B)	1.392(8)	S(1B)-O(3B)	1.405(11)
S(1B)-C(17B)	1.802(10)	F(1B)-C(17B)	1.377(7)
F(2B)-C(17B)	1.365(8)	F(3B)-C(17B)	1.379(8)
N(3)-C(18)	1.140(8)	C(18)-C(19)	1.467(9)
C(19)-H(19A)	0.9800	C(19)-H(19B)	0.9800
C(19)-H(19C)	0.9800		

N(1)-Bi(1)-Bi(1)#1	100.02(3)	N(1)-Bi(1)-N(2)	142.65(5)
N(2)-Bi(1)-Bi(1)#1	87.67(4)	C(1)-Bi(1)-Bi(1)#1	99.31(4)
C(1)-Bi(1)-N(1)	71.82(6)	C(1)-Bi(1)-N(2)	70.87(7)
C(7)-N(1)-Bi(1)	111.86(12)	C(7)-N(1)-C(9)	120.26(15)
C(9)-N(1)-Bi(1)	127.24(10)	C(8)-N(2)-Bi(1)	110.49(14)
C(8)-N(2)-C(13)	119.51(18)	C(13)-N(2)-Bi(1)	128.96(13)
C(2)-C(1)-Bi(1)	118.81(12)	C(2)-C(1)-C(6)	120.93(17)
C(6)-C(1)-Bi(1)	119.74(15)	C(1)-C(2)-C(3)	119.42(18)
C(1)-C(2)-C(7)	117.57(15)	C(3)-C(2)-C(7)	122.89(19)
C(2)-C(3)-H(3)	120.1	C(4)-C(3)-C(2)	119.9(2)
C(4)-C(3)-H(3)	120.1	C(3)-C(4)-H(4)	119.8
C(5)-C(4)-C(3)	120.36(19)	C(5)-C(4)-H(4)	119.8
C(4)-C(5)-H(5)	120.0	C(4)-C(5)-C(6)	119.98(19)
C(6)-C(5)-H(5)	120.0	C(1)-C(6)-C(5)	119.3(2)
C(1)-C(6)-C(8)	118.22(18)	C(5)-C(6)-C(8)	122.37(18)
N(1)-C(7)-C(2)	119.70(17)	N(1)-C(7)-H(7)	120.2
C(2)-C(7)-H(7)	120.2	N(2)-C(8)-C(6)	120.22(17)
N(2)-C(8)-H(8)	119.9	C(6)-C(8)-H(8)	119.9
N(1)-C(9)-C(10)	109.07(13)	N(1)-C(9)-C(11)	106.99(13)
N(1)-C(9)-C(12)	109.54(13)	C(10)-C(9)-C(12)	111.02(14)
C(11)-C(9)-C(10)	110.68(14)	C(11)-C(9)-C(12)	109.45(14)
C(9)-C(10)-H(10A)	109.5	C(9)-C(10)-H(10B)	109.5
C(9)-C(10)-H(10C)	109.5	H(10A)-C(10)-H(10B)	109.5
H(10A)-C(10)-H(10C)	109.5	H(10B)-C(10)-H(10C)	109.5
C(9)-C(11)-H(11A)	109.5	C(9)-C(11)-H(11B)	109.5
C(9)-C(11)-H(11C)	109.5	H(11A)-C(11)-H(11B)	109.5
H(11A)-C(11)-H(11C)	109.5	H(11B)-C(11)-H(11C)	109.5
C(9)-C(12)-H(12A)	109.5	C(9)-C(12)-H(12B)	109.5

C(9)-C(12)-H(12C)	109.5	H(12A)-C(12)-H(12B)	109.5
H(12A)-C(12)-H(12C)	109.5	H(12B)-C(12)-H(12C)	109.5
N(2)-C(13)-C(15A)	103.2(3)	N(2)-C(13)-C(16A)	114.2(4)
N(2)-C(13)-C(14B)	108.0(4)	N(2)-C(13)-C(16B)	106.9(5)
C(14A)-C(13)-N(2)	106.4(2)	C(14A)-C(13)-C(15A)	109.5(3)
C(14A)-C(13)-C(16A)	116.9(5)	C(16A)-C(13)-C(15A)	105.7(5)
C(15B)-C(13)-N(2)	115.1(5)	C(15B)-C(13)-C(14B)	103.3(5)
C(15B)-C(13)-C(16B)	119.3(6)	C(16B)-C(13)-C(14B)	103.1(6)
C(13)-C(14A)-H(14A)	109.5	C(13)-C(14A)-H(14B)	109.5
C(13)-C(14A)-H(14C)	109.5	H(14A)-C(14A)-H(14B)	109.5
H(14A)-C(14A)-H(14C)	109.5	H(14B)-C(14A)-H(14C)	109.5
C(13)-C(15A)-H(15A)	109.5	C(13)-C(15A)-H(15B)	109.5
C(13)-C(15A)-H(15C)	109.5	H(15A)-C(15A)-H(15B)	109.5
H(15A)-C(15A)-H(15C)	109.5	H(15B)-C(15A)-H(15C)	109.5
C(13)-C(16A)-H(16A)	109.5	C(13)-C(16A)-H(16B)	109.5
C(13)-C(16A)-H(16C)	109.5	H(16A)-C(16A)-H(16B)	109.5
H(16A)-C(16A)-H(16C)	109.5	H(16B)-C(16A)-H(16C)	109.5
C(13)-C(14B)-H(14D)	109.5	C(13)-C(14B)-H(14E)	109.5
C(13)-C(14B)-H(14F)	109.5	H(14D)-C(14B)-H(14E)	109.5
H(14D)-C(14B)-H(14F)	109.5	H(14E)-C(14B)-H(14F)	109.5
C(13)-C(15B)-H(15D)	109.5	C(13)-C(15B)-H(15E)	109.5
C(13)-C(15B)-H(15F)	109.5	H(15D)-C(15B)-H(15E)	109.5
H(15D)-C(15B)-H(15F)	109.5	H(15E)-C(15B)-H(15F)	109.5
C(13)-C(16B)-H(16D)	109.5	C(13)-C(16B)-H(16E)	109.5
C(13)-C(16B)-H(16F)	109.5	H(16D)-C(16B)-H(16E)	109.5
H(16D)-C(16B)-H(16F)	109.5	H(16E)-C(16B)-H(16F)	109.5
N(4)-C(20)-C(21)	180.0	C(20)-C(21)-H(21A)	112.4
C(20)-C(21)-H(21C)	112.4	C(20)-C(21)-H(21B)	112.4
H(21A)-C(21)-H(21C)	106.4	H(21A)-C(21)-H(21B)	106.4
H(21C)-C(21)-H(21B)	106.4	O(1A)-S(1A)-O(2A)	114.23(17)
O(1A)-S(1A)-O(3A)	114.3(3)	O(1A)-S(1A)-C(17A)	102.71(19)
O(2A)-S(1A)-O(3A)	116.3(4)	O(2A)-S(1A)-C(17A)	103.85(19)
O(3A)-S(1A)-C(17A)	103.1(4)	F(1A)-C(17A)-S(1A)	108.5(3)
F(1A)-C(17A)-F(3A)	114.6(4)	F(2A)-C(17A)-S(1A)	111.3(3)
F(2A)-C(17A)-F(1A)	108.5(4)	F(2A)-C(17A)-F(3A)	110.0(5)
F(3A)-C(17A)-S(1A)	103.9(4)	O(1B)-S(1B)-C(17B)	104.7(4)

O(2B)-S(1B)-O(1B)	114.8(5)	O(2B)-S(1B)-O(3B)	114.1(9)
O(2B)-S(1B)-C(17B)	102.4(5)	O(3B)-S(1B)-O(1B)	117.5(9)
O(3B)-S(1B)-C(17B)	100.3(8)	F(1B)-C(17B)-S(1B)	121.5(8)
F(1B)-C(17B)-F(3B)	80.5(9)	F(2B)-C(17B)-S(1B)	116.6(7)
F(2B)-C(17B)-F(1B)	101.5(8)	F(2B)-C(17B)-F(3B)	100.9(10)
F(3B)-C(17B)-S(1B)	128.3(9)	N(3)-C(18)-C(19)	178(2)
C(18)-C(19)-H(19A)	109.5	C(18)-C(19)-H(19B)	109.5
C(18)-C(19)-H(19C)	109.5	H(19A)-C(19)-H(19B)	109.5
H(19A)-C(19)-H(19C)	109.5	H(19B)-C(19)-H(19C)	109.5

Symmetry transformations used to generate equivalent atoms:

#1 -y+1,-x+1,-z+1/2

7.2 Single crystal structure analysis of **3** MeCN solvate

C_{36} H_{49} Bi_2 F_6 N_5 O_6 S_3, M_r = 1275.94 g mol^{-1}, yellow prism, crystal size 0.115 x 0.106 x 0.084 mm^3, orthorhombic, space group $P2_12_12$ [18], a = 15.1730(9) Å, b = 17.0083(10) Å, c = 8.4981(5) Å, V = 2193.1(2) Å^3,T = 100(2) K, Z = 2, D_{calc} = 1.932 g·cm^3, λ = 0.71073 Å, μ(Mo-K_α) = 8.230 mm^{-1}, Gaussian correction ($T_{\min}$ = 0.49587, $T_{\max}$ = 0.64594), Bruker-AXS Kappa Mach3 with APEX-II detector and IµS micro focus Mo-anode X-ray source, 1.799 < θ < 32.560°, 79659 measured reflections, 7992 independent reflections, 7510 reflections with $I > 2\sigma(I)$, R_{int} = 0.0443. The structure was solved by *SHELXT* and refined by full-matrix least-squares (*SHELXL*) against F^2 to R_1 = 0.0189 [$I > 2\sigma(I)$], wR_2 = 0.0412 [all data], 322parameters, 12 restraints and an absolute structure parameter Flack x –0.0288(18).

Figure S38: The solid state structure of **3** MeCN solvate. H atoms have been removed for clarity and disordered regions shown in grey.

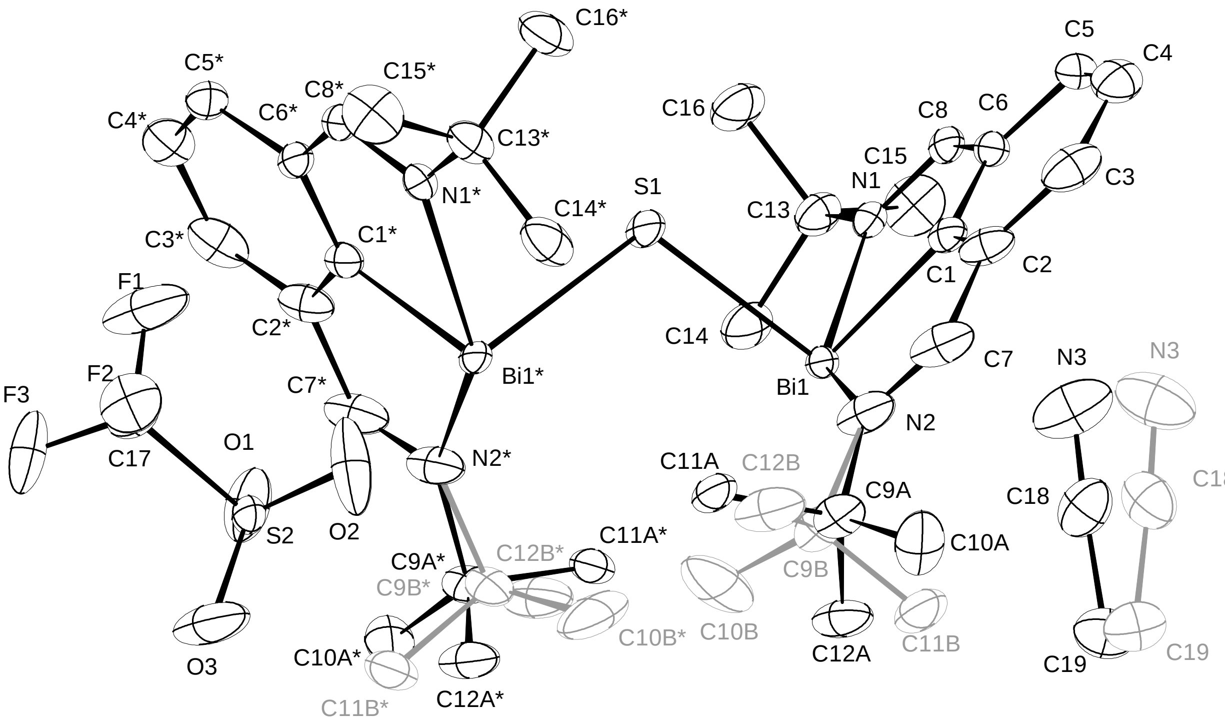

Figure S39: Crystal faces and unit cell determination/refinement of **3** MeCN solvate.

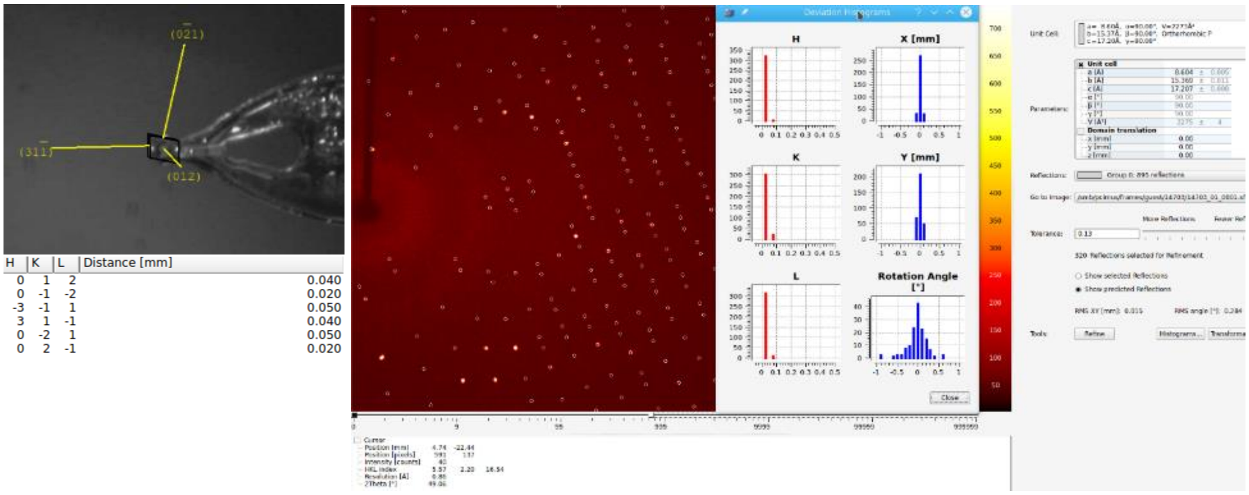

INTENSITY STATISTICS FOR DATASET

Resolution	#Data	#Theory	%Complete	Redundancy	Mean I	Mean I/s	Rmerge	Rsigma
Inf - 2.65	132	132	100.0	17.35	134.20	90.22	0.0291	0.0100
2.65 - 1.74	306	306	100.0	18.73	94.17	92.44	0.0290	0.0098
1.74 - 1.39	439	439	100.0	18.49	61.22	84.41	0.0312	0.0104
1.39 - 1.20	459	459	100.0	18.31	47.04	76.15	0.0359	0.0111
1.20 - 1.09	449	449	100.0	17.10	35.90	66.29	0.0434	0.0124
1.09 - 1.02	404	404	100.0	12.96	30.64	53.37	0.0500	0.0157
1.02 - 0.95	492	492	100.0	10.43	25.13	43.29	0.0577	0.0193
0.95 - 0.91	387	387	100.0	9.30	20.87	36.49	0.0669	0.0228
0.91 - 0.87	431	431	100.0	8.09	18.30	31.08	0.0698	0.0265
0.87 - 0.83	531	531	100.0	7.83	15.59	27.73	0.0748	0.0305
0.83 - 0.80	489	489	100.0	7.47	14.03	24.34	0.0817	0.0344
0.80 - 0.78	344	344	100.0	7.10	13.72	22.11	0.0857	0.0375
0.78 - 0.75	617	617	100.0	6.93	12.01	20.65	0.0919	0.0413
0.75 - 0.74	234	234	100.0	6.84	9.82	17.62	0.1052	0.0482
0.74 - 0.72	477	477	100.0	6.45	9.07	15.84	0.1139	0.0541
0.72 - 0.70	545	545	100.0	6.25	8.01	14.32	0.1180	0.0626
0.70 - 0.69	300	300	100.0	6.15	7.78	13.69	0.1259	0.0644
0.69 - 0.67	616	616	100.0	5.85	6.81	11.54	0.1444	0.0799
0.67 - 0.66	364	364	100.0	5.74	6.59	10.83	0.1499	0.0862
0.66 - 0.65	365	365	100.0	5.66	5.95	10.02	0.1618	0.0907
0.65 - 0.64	374	415	90.1	3.77	5.05	8.11	0.1581	0.1238
0.74 - 0.64	3041	3082	98.7	5.73	7.13	12.24	0.1328	0.0752
Inf - 0.64	8755	8796	99.5	9.49	23.19	34.34	0.0449	0.0232

Complete .cif-data of the compound are available under the CCDC number CCDC-**2351151**.

A resolution cut-off (SHEL 999 0.66) was applied to the data set to exclude the poorly determined reflections at high diffraction angles. The ISOR instruction was applied to treat the thermal elipsoides of N2 and C7. A tert-butyl group is disordered over two positions. The two parts were each refined with a fixed composition of 50:50%.

Table S7: Crystal data and structure refinement of **3** MeCN solvate.

Identification code	14703	
Empirical formula	C_{36} H_{49} Bi_{2} F_{6} N_{5} O_{6} S_{3}	
Color	yellow	
Formula weight	1275.94 g·mol^{-1}	
Temperature	100(2) K	
Wavelength	0.71073 Å	
Crystal system	Orthorhombic	
Space group	$P2_12_12$, (no. 18)	
Unit cell dimensions	a = 15.1730(9) Å	α= 90°.
	b = 17.0083(10) Å	β= 90°.
	c = 8.4981(5) Å	γ = 90°.
Volume	2193.1(2) Å^3	
Z	2	
Density (calculated)	1.932 Mg·m^{-3}	
Absorption coefficient	8.230 mm^{-1}	
F(000)	1232 e	
Crystal size	0.115 x 0.106 x 0.084 mm^3	
θ range for data collection	1.799 to 32.560°.	
Index ranges	$-22 \le h \le 22$, $-25 \le k \le 25$, $-12 \le l \le 12$	
Reflections collected	79659	
Independent reflections	7992 [R_{int} = 0.0443]	
Reflections with $I>2\sigma(I)$	7510	
Completeness to θ = 25.242°	100.0%	
Absorption correction	Gaussian	
Max. and min. transmission	0.64594 and 0.49587	
Refinement method	Full-matrix least-squares on F^2	
Data / restraints / parameters	7992 / 12 / 322	
Goodness-of-fit on F^2	1.055	
Final R indices [$I>2\sigma(I)$]	R_1 = 0.0189	wR^2 = 0.0400
R indices (all data)	R_1 = 0.0224	wR^2 = 0.0412
Absolute structure parameter	-0.0288(18)	
Extinction coefficient	n/a	
Largest diff. peak and hole	1.205 and -1.983 e·Å^{-3}	

Table S8: Bond lengths [Å] and angles [°] of **3** MeCN solvate.

Bi(1)-S(1)	2.5497(8)	Bi(1)-N(1)	2.453(3)
Bi(1)-N(2)	2.572(4)	Bi(1)-C(1)	2.200(3)
N(1)-C(8)	1.276(4)	N(1)-C(13)	1.493(5)
N(2)-C(7)	1.266(6)	N(2)-C(9A)	1.517(17)
N(2)-C(9B)	1.515(18)	C(1)-C(2)	1.384(5)
C(1)-C(6)	1.384(4)	C(2)-C(3)	1.395(6)
C(2)-C(7)	1.468(5)	C(3)-H(3)	0.9500
C(3)-C(4)	1.387(6)	C(4)-H(4)	0.9500
C(4)-C(5)	1.392(6)	C(5)-H(5)	0.9500
C(5)-C(6)	1.396(5)	C(6)-C(8)	1.463(5)
C(7)-H(7)	0.9500	C(8)-H(8)	0.9500
C(13)-C(14)	1.518(6)	C(13)-C(15)	1.523(6)
C(13)-C(16)	1.525(5)	C(14)-H(14A)	0.9800
C(14)-H(14B)	0.9800	C(14)-H(14C)	0.9800
C(15)-H(15A)	0.9800	C(15)-H(15B)	0.9800
C(15)-H(15C)	0.9800	C(16)-H(16A)	0.9800
C(16)-H(16B)	0.9800	C(16)-H(16C)	0.9800
C(9A)-C(10A)	1.509(16)	C(9A)-C(11A)	1.51(2)
C(9A)-C(12A)	1.543(17)	C(10A)-H(10A)	0.9800
C(10A)-H(10B)	0.9800	C(10A)-H(10C)	0.9800
C(11A)-H(11A)	0.9800	C(11A)-H(11B)	0.9800
C(11A)-H(11C)	0.9800	C(12A)-H(12A)	0.9800
C(12A)-H(12B)	0.9800	C(12A)-H(12C)	0.9800
C(9B)-C(10B)	1.554(18)	C(9B)-C(11B)	1.49(2)
C(9B)-C(12B)	1.517(18)	C(10B)-H(10D)	0.9800
C(10B)-H(10E)	0.9800	C(10B)-H(10F)	0.9800
C(11B)-H(11D)	0.9800	C(11B)-H(11E)	0.9800
C(11B)-H(11F)	0.9800	C(12B)-H(12D)	0.9800
C(12B)-H(12E)	0.9800	C(12B)-H(12F)	0.9800
S(2)-O(1)	1.424(3)	S(2)-O(2)	1.421(3)
S(2)-O(3)	1.414(3)	S(2)-C(17)	1.823(4)
F(3)-C(17)	1.313(5)	C(17)-F(1)	1.336(5)
C(17)-F(2)	1.321(4)	N(3)-C(18)	1.147(12)

C(18)-C(19)	1.458(13)	C(19)-H(19A)	0.9800
C(19)-H(19B)	0.9800	C(19)-H(19C)	0.9800
S(1)-Bi(1)-N(2)	98.40(7)	N(1)-Bi(1)-S(1)	83.39(7)
N(1)-Bi(1)-N(2)	142.26(11)	C(1)-Bi(1)-S(1)	93.58(8)
C(1)-Bi(1)-N(1)	72.04(11)	C(1)-Bi(1)-N(2)	70.22(12)
Bi(1)#1-S(1)-Bi(1)	107.54(5)	C(8)-N(1)-Bi(1)	112.3(2)
C(8)-N(1)-C(13)	120.9(3)	C(13)-N(1)-Bi(1)	126.8(2)
C(7)-N(2)-Bi(1)	110.8(2)	C(7)-N(2)-C(9A)	112.7(6)
C(7)-N(2)-C(9B)	130.9(6)	C(9A)-N(2)-Bi(1)	135.5(6)
C(9B)-N(2)-Bi(1)	117.1(5)	C(2)-C(1)-Bi(1)	120.4(2)
C(6)-C(1)-Bi(1)	118.0(2)	C(6)-C(1)-C(2)	121.5(3)
C(1)-C(2)-C(3)	118.9(3)	C(1)-C(2)-C(7)	118.0(4)
C(3)-C(2)-C(7)	122.9(4)	C(2)-C(3)-H(3)	119.8
C(4)-C(3)-C(2)	120.3(3)	C(4)-C(3)-H(3)	119.8
C(3)-C(4)-H(4)	119.9	C(3)-C(4)-C(5)	120.1(4)
C(5)-C(4)-H(4)	119.9	C(4)-C(5)-H(5)	120.1
C(4)-C(5)-C(6)	119.8(3)	C(6)-C(5)-H(5)	120.1
C(1)-C(6)-C(5)	119.3(3)	C(1)-C(6)-C(8)	117.8(3)
C(5)-C(6)-C(8)	122.9(3)	N(2)-C(7)-C(2)	120.4(4)
N(2)-C(7)-H(7)	119.8	C(2)-C(7)-H(7)	119.8
N(1)-C(8)-C(6)	119.8(3)	N(1)-C(8)-H(8)	120.1
C(6)-C(8)-H(8)	120.1	N(1)-C(13)-C(14)	107.0(3)
N(1)-C(13)-C(15)	110.6(3)	N(1)-C(13)-C(16)	108.0(3)
C(14)-C(13)-C(15)	110.5(4)	C(14)-C(13)-C(16)	110.0(3)
C(15)-C(13)-C(16)	110.8(4)	C(13)-C(14)-H(14A)	109.5
C(13)-C(14)-H(14B)	109.5	C(13)-C(14)-H(14C)	109.5
H(14A)-C(14)-H(14B)	109.5	H(14A)-C(14)-H(14C)	109.5
H(14B)-C(14)-H(14C)	109.5	C(13)-C(15)-H(15A)	109.5
C(13)-C(15)-H(15B)	109.5	C(13)-C(15)-H(15C)	109.5
H(15A)-C(15)-H(15B)	109.5	H(15A)-C(15)-H(15C)	109.5
H(15B)-C(15)-H(15C)	109.5	C(13)-C(16)-H(16A)	109.5
C(13)-C(16)-H(16B)	109.5	C(13)-C(16)-H(16C)	109.5
H(16A)-C(16)-H(16B)	109.5	H(16A)-C(16)-H(16C)	109.5
H(16B)-C(16)-H(16C)	109.5	N(2)-C(9A)-C(12A)	100.7(9)
C(10A)-C(9A)-N(2)	120.0(12)	C(10A)-C(9A)-C(11A)	112.6(11)

C(10A)-C(9A)-C(12A)	109.6(13)	C(11A)-C(9A)-N(2)	103.2(11)
C(11A)-C(9A)-C(12A)	109.9(12)	C(9A)-C(10A)-H(10A)	109.5
C(9A)-C(10A)-H(10B)	109.5	C(9A)-C(10A)-H(10C)	109.5
H(10A)-C(10A)-H(10B)	109.5	H(10A)-C(10A)-H(10C)	109.5
H(10B)-C(10A)-H(10C)	109.5	C(9A)-C(11A)-H(11A)	109.5
C(9A)-C(11A)-H(11B)	109.5	C(9A)-C(11A)-H(11C)	109.5
H(11A)-C(11A)-H(11B)	109.5	H(11A)-C(11A)-H(11C)	109.5
H(11B)-C(11A)-H(11C)	109.5	C(9A)-C(12A)-H(12A)	109.5
C(9A)-C(12A)-H(12B)	109.5	C(9A)-C(12A)-H(12C)	109.5
H(12A)-C(12A)-H(12B)	109.5	H(12A)-C(12A)-H(12C)	109.5
H(12B)-C(12A)-H(12C)	109.5	N(2)-C(9B)-C(10B)	115.5(9)
N(2)-C(9B)-C(12B)	108.7(11)	C(11B)-C(9B)-N(2)	105.0(12)
C(11B)-C(9B)-C(10B)	108.6(12)	C(11B)-C(9B)-C(12B)	112.6(10)
C(12B)-C(9B)-C(10B)	106.7(14)	C(9B)-C(10B)-H(10D)	109.5
C(9B)-C(10B)-H(10E)	109.5	C(9B)-C(10B)-H(10F)	109.5
H(10D)-C(10B)-H(10E)	109.5	H(10D)-C(10B)-H(10F)	109.5
H(10E)-C(10B)-H(10F)	109.5	C(9B)-C(11B)-H(11D)	109.5
C(9B)-C(11B)-H(11E)	109.5	C(9B)-C(11B)-H(11F)	109.5
H(11D)-C(11B)-H(11E)	109.5	H(11D)-C(11B)-H(11F)	109.5
H(11E)-C(11B)-H(11F)	109.5	C(9B)-C(12B)-H(12D)	109.5
C(9B)-C(12B)-H(12E)	109.5	C(9B)-C(12B)-H(12F)	109.5
H(12D)-C(12B)-H(12E)	109.5	H(12D)-C(12B)-H(12F)	109.5
H(12E)-C(12B)-H(12F)	109.5	O(1)-S(2)-C(17)	104.36(19)
O(2)-S(2)-O(1)	113.2(2)	O(2)-S(2)-C(17)	103.1(2)
O(3)-S(2)-O(1)	116.5(3)	O(3)-S(2)-O(2)	115.1(3)
O(3)-S(2)-C(17)	102.2(2)	F(3)-C(17)-S(2)	112.0(3)
F(3)-C(17)-F(1)	106.4(4)	F(3)-C(17)-F(2)	107.1(3)
F(1)-C(17)-S(2)	110.8(3)	F(2)-C(17)-S(2)	112.3(3)
F(2)-C(17)-F(1)	108.0(3)	N(3)-C(18)-C(19)	177.0(13)
C(18)-C(19)-H(19A)	109.5	C(18)-C(19)-H(19B)	109.5
C(18)-C(19)-H(19C)	109.5	H(19A)-C(19)-H(19B)	109.5
H(19A)-C(19)-H(19C)	109.5	H(19B)-C(19)-H(19C)	109.5

Symmetry transformations used to generate equivalent atoms:

#1 -x+1,-y+1,z

7.3 Single crystal structure analysis of **4** pentane solvate

C_{51} H_{68} Bi_2 F_6 N_4 O_6 S_4, M_r = 1493.29 g mol^{-1}, yellow plate, crystal size 0.051 x 0.042 x 0.018 mm^3, monoclinic, space group *C*2/*c* [15], *a* = 37.6761(15) Å, *b* = 8.5550(3) Å, *c* = 17.3531(7) Å, β = 90.927(2)°, *V* = 5592.5(4) Å^3,*T* = 100(2) K, *Z* = 4, D_{calc} = 1.774 g·cm^3, λ = 0.71073 Å, μ(*Mo*-K_α) = 6.504 mm^{-1}, Gaussian correction ($T_{\min}$ = 0.75774, $T_{\max}$ = 0.90805), Bruker-AXS Kappa Mach3 with APEX-II detector and IµS micro focus Mo-anode X-ray source, 1.081 < θ < 30.508°, 86800 measured reflections, 8547 independent reflections, 7011 reflections with $I > 2\sigma(I)$, R_{int} = 0.0546. The structure was solved by *SHELXT* and refined by full-matrix least-squares (*SHELXL*) against F^2 to R_1 = 0.0263 [$I > 2\sigma(I)$], wR_2 = 0.0492 [all data], 337 parameters and 0 restraints.

Figure S40: The solid state structure of **4** pentane solvate. H atoms have been removed for clarity and disordered regions shown in grey.

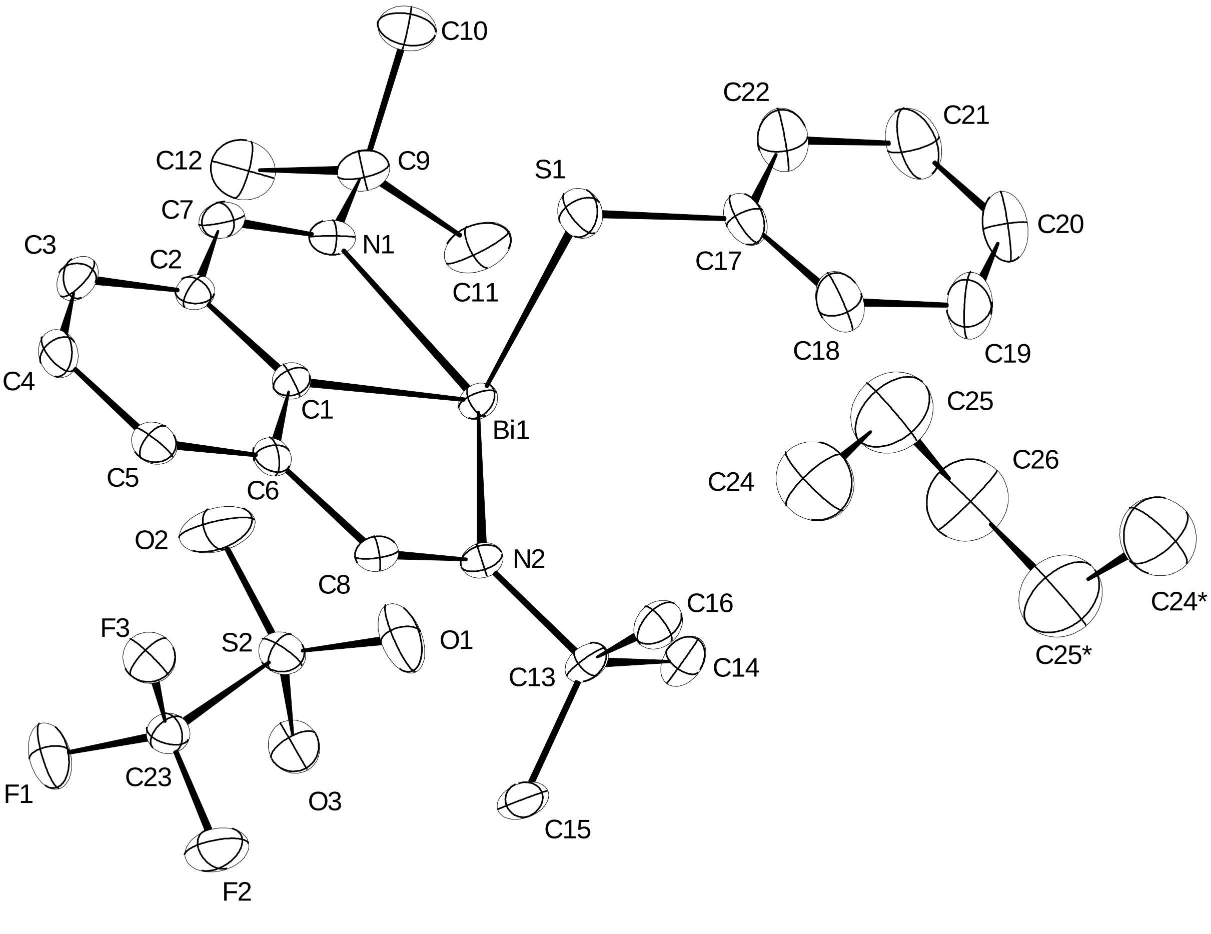

Figure S41: Crystal faces and unit cell determination/refinement of **4** pentane solvate.

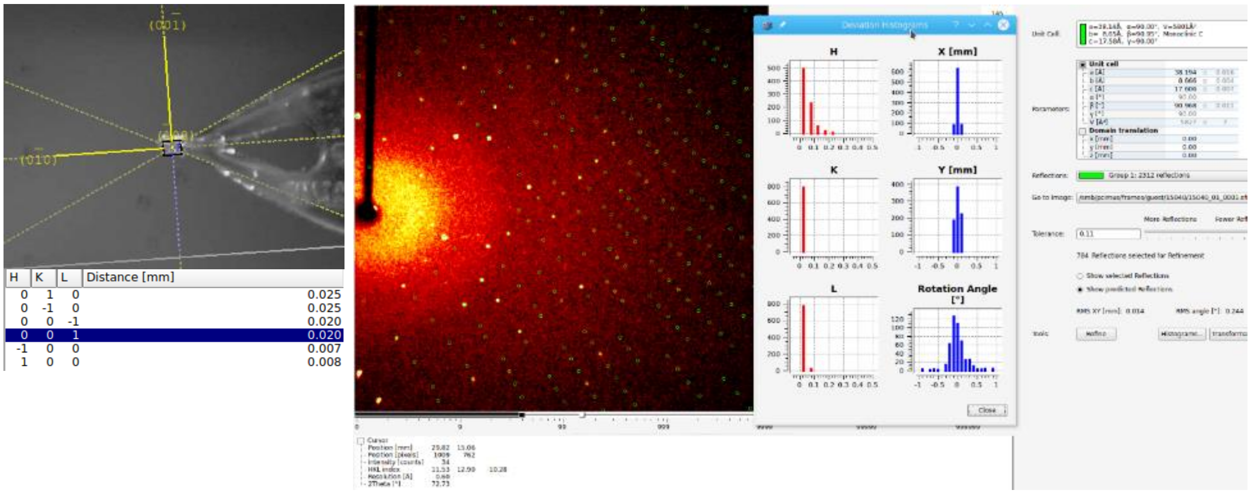

INTENSITY STATISTICS FOR DATASET

Resolution	#Data	#Theory	%Complete	Redundancy	Mean I	Mean I/s	Rmerge	Rsigma
Inf - 2.88	149	149	100.0	15.03	83.61	75.64	0.0232	0.0084
2.88 - 1.90	349	349	100.0	16.30	62.65	70.23	0.0233	0.0094
1.90 - 1.49	501	501	100.0	16.90	48.91	60.62	0.0270	0.0105
1.49 - 1.30	493	493	100.0	16.72	34.74	50.94	0.0352	0.0130
1.30 - 1.17	539	539	100.0	16.62	29.63	46.38	0.0440	0.0151
1.17 - 1.08	506	506	100.0	15.20	24.52	39.06	0.0534	0.0184
1.08 - 1.02	471	471	100.0	11.59	23.84	32.35	0.0580	0.0236
1.02 - 0.97	470	470	100.0	10.10	18.62	24.17	0.0705	0.0309
0.97 - 0.92	577	577	100.0	8.71	16.84	20.92	0.0829	0.0382
0.92 - 0.89	434	434	100.0	7.95	15.00	17.57	0.0939	0.0453
0.89 - 0.85	635	635	100.0	7.44	13.85	15.56	0.1011	0.0522
0.85 - 0.83	383	383	100.0	7.19	11.93	13.49	0.1199	0.0617
0.83 - 0.80	621	621	100.0	6.98	11.05	12.15	0.1290	0.0690
0.80 - 0.78	457	457	100.0	6.75	10.81	11.41	0.1401	0.0750
0.78 - 0.76	534	534	100.0	6.54	9.74	10.13	0.1572	0.0857
0.76 - 0.74	584	584	100.0	6.30	8.51	8.87	0.1757	0.1016
0.74 - 0.73	322	322	100.0	6.34	7.54	7.90	0.1967	0.1152
0.73 - 0.71	654	656	99.7	6.03	8.07	7.90	0.1974	0.1157
0.71 - 0.70	386	386	100.0	5.89	6.18	6.16	0.2466	0.1525
0.70 - 0.69	387	387	100.0	5.70	6.01	5.75	0.2624	0.1625
0.69 - 0.68	489	502	97.4	4.04	5.86	4.69	0.2365	0.2119
0.78 - 0.68	3356	3371	99.6	5.84	7.59	7.51	0.1974	0.1253
Inf - 0.68	9941	9956	99.8	9.50	19.23	23.67	0.0555	0.0378

Complete .cif-data of the compound are available under the CCDC number CCDC-**2351152**.

A resolution cut-off (SHEL 999 0.7) was applied to the data set to exclude the poorly determined reflections at high diffraction angles.

Table S9: Crystal data and structure refinement of **4** pentane solvate.

Identification code	15040	
Empirical formula	$C_{51} H_{68} Bi_2 F_6 N_4 O_6 S_4$	
Color	yellow	
Formula weight	1493.29 g·mol^{-1}	
Temperature	100(2) K	
Wavelength	0.71073 Å	
Crystal system	Monoclinic	
Space group	*C*2/*c*, (no. 15)	
Unit cell dimensions	a = 37.6761(15) Å	α= 90°.
	b = 8.5550(3) Å	β= 90.927(2)°.
	c = 17.3531(7) Å	γ = 90°.
Volume	5592.5(4) Å^3	
Z	4	
Density (calculated)	1.774 Mg·m^{-3}	
Absorption coefficient	6.504 mm^{-1}	
F(000)	2936 e	
Crystal size	0.051 x 0.042 x 0.018 mm^3	
θ range for data collection	1.081 to 30.508°.	
Index ranges	-53 ≤ h ≤ 53, -12≤ k ≤ 12, -24 ≤ l ≤ 24	
Reflections collected	86800	
Independent reflections	8547 [R_{int} = 0.0546]	
Reflections with I>2σ(I)	7011	
Completeness to θ = 25.242°	100.0%	
Absorption correction	Gaussian	
Max. and min. transmission	0.90805 and 0.75774	
Refinement method	Full-matrix least-squares on F^2	
Data / restraints / parameters	8547 / 0 / 337	
Goodness-of-fit on F^2	1.031	
Final R indices [I>2σ(I)]	R_1 = 0.0263	wR^2 = 0.0454
R indices (all data)	R_1 = 0.0414	wR^2 = 0.0492
Extinction coefficient	n/a	
Largest diff. peak and hole	1.607 and -1.449 e·Å^{-3}	

Table S10: Bond lengths [Å] and angles [°] of **4** pentane solvate.

Bi(1)-S(1)	2.5702(8)	Bi(1)-N(1)	2.491(2)
Bi(1)-N(2)	2.488(2)	Bi(1)-C(1)	2.194(3)
S(1)-C(17)	1.780(3)	S(2)-O(1)	1.443(2)
S(2)-O(2)	1.436(2)	S(2)-O(3)	1.433(2)
S(2)-C(23)	1.823(3)	F(1)-C(23)	1.326(3)
F(2)-C(23)	1.330(3)	F(3)-C(23)	1.329(3)
N(1)-C(7)	1.273(4)	N(1)-C(9)	1.487(4)
N(2)-C(8)	1.276(4)	N(2)-C(13)	1.496(3)
C(1)-C(2)	1.385(4)	C(1)-C(6)	1.387(4)
C(2)-C(3)	1.392(4)	C(2)-C(7)	1.469(4)
C(3)-H(3)	0.9500	C(3)-C(4)	1.391(4)
C(4)-H(4)	0.9500	C(4)-C(5)	1.385(4)
C(5)-H(5)	0.9500	C(5)-C(6)	1.391(4)
C(6)-C(8)	1.473(4)	C(7)-H(7)	0.9500
C(8)-H(8)	0.9500	C(9)-C(10)	1.524(4)
C(9)-C(11)	1.522(4)	C(9)-C(12)	1.523(4)
C(10)-H(10A)	0.9800	C(10)-H(10B)	0.9800
C(10)-H(10C)	0.9800	C(11)-H(11A)	0.9800
C(11)-H(11B)	0.9800	C(11)-H(11C)	0.9800
C(12)-H(12A)	0.9800	C(12)-H(12B)	0.9800
C(12)-H(12C)	0.9800	C(13)-C(14)	1.522(4)
C(13)-C(15)	1.528(4)	C(13)-C(16)	1.521(4)
C(14)-H(14C)	0.9800	C(14)-H(14A)	0.9800
C(14)-H(14B)	0.9800	C(15)-H(15A)	0.9800
C(15)-H(15B)	0.9800	C(15)-H(15C)	0.9800
C(16)-H(16A)	0.9800	C(16)-H(16B)	0.9800
C(16)-H(16C)	0.9800	C(17)-C(18)	1.385(5)
C(17)-C(22)	1.383(4)	C(18)-H(18)	0.9500
C(18)-C(19)	1.389(5)	C(19)-H(19)	0.9500
C(19)-C(20)	1.369(5)	C(20)-H(20)	0.9500
C(20)-C(21)	1.370(5)	C(21)-H(21)	0.9500
C(21)-C(22)	1.392(5)	C(22)-H(22)	0.9500

C(24)-H(24A)	0.9800	C(24)-H(24B)	0.9800
C(24)-H(24C)	0.9800	C(24)-C(25)	1.484(7)
C(25)-H(25A)	0.9900	C(25)-H(25B)	0.9900
C(25)-C(26)	1.508(6)	C(26)-H(26A)	0.9900
C(26)-H(26B)	0.9900		

N(1)-Bi(1)-S(1)	90.68(6)	N(2)-Bi(1)-S(1)	92.39(6)
N(2)-Bi(1)-N(1)	142.47(8)	C(1)-Bi(1)-S(1)	89.30(7)
C(1)-Bi(1)-N(1)	71.37(9)	C(1)-Bi(1)-N(2)	71.28(9)
C(17)-S(1)-Bi(1)	98.22(10)	O(1)-S(2)-C(23)	102.11(14)
O(2)-S(2)-O(1)	114.25(16)	O(2)-S(2)-C(23)	103.99(14)
O(3)-S(2)-O(1)	114.42(14)	O(3)-S(2)-O(2)	115.56(15)
O(3)-S(2)-C(23)	104.31(13)	C(7)-N(1)-Bi(1)	112.11(19)
C(7)-N(1)-C(9)	122.2(2)	C(9)-N(1)-Bi(1)	125.68(18)
C(8)-N(2)-Bi(1)	112.58(18)	C(8)-N(2)-C(13)	120.8(2)
C(13)-N(2)-Bi(1)	126.46(17)	C(2)-C(1)-Bi(1)	119.2(2)
C(2)-C(1)-C(6)	121.4(3)	C(6)-C(1)-Bi(1)	119.4(2)
C(1)-C(2)-C(3)	119.0(3)	C(1)-C(2)-C(7)	117.5(2)
C(3)-C(2)-C(7)	123.5(3)	C(2)-C(3)-H(3)	120.1
C(4)-C(3)-C(2)	119.8(3)	C(4)-C(3)-H(3)	120.1
C(3)-C(4)-H(4)	119.6	C(5)-C(4)-C(3)	120.7(3)
C(5)-C(4)-H(4)	119.6	C(4)-C(5)-H(5)	120.2
C(4)-C(5)-C(6)	119.6(3)	C(6)-C(5)-H(5)	120.2
C(1)-C(6)-C(5)	119.4(3)	C(1)-C(6)-C(8)	117.5(2)
C(5)-C(6)-C(8)	123.0(3)	N(1)-C(7)-C(2)	119.7(3)
N(1)-C(7)-H(7)	120.1	C(2)-C(7)-H(7)	120.1
N(2)-C(8)-C(6)	119.2(2)	N(2)-C(8)-H(8)	120.4
C(6)-C(8)-H(8)	120.4	N(1)-C(9)-C(10)	107.4(2)
N(1)-C(9)-C(11)	106.8(2)	N(1)-C(9)-C(12)	111.1(3)
C(11)-C(9)-C(10)	110.4(3)	C(11)-C(9)-C(12)	110.2(3)
C(12)-C(9)-C(10)	110.8(3)	C(9)-C(10)-H(10A)	109.5
C(9)-C(10)-H(10B)	109.5	C(9)-C(10)-H(10C)	109.5
H(10A)-C(10)-H(10B)	109.5	H(10A)-C(10)-H(10C)	109.5
H(10B)-C(10)-H(10C)	109.5	C(9)-C(11)-H(11A)	109.5
C(9)-C(11)-H(11B)	109.5	C(9)-C(11)-H(11C)	109.5
H(11A)-C(11)-H(11B)	109.5	H(11A)-C(11)-H(11C)	109.5

H(11B)-C(11)-H(11C)	109.5	C(9)-C(12)-H(12A)	109.5
C(9)-C(12)-H(12B)	109.5	C(9)-C(12)-H(12C)	109.5
H(12A)-C(12)-H(12B)	109.5	H(12A)-C(12)-H(12C)	109.5
H(12B)-C(12)-H(12C)	109.5	N(2)-C(13)-C(14)	107.2(2)
N(2)-C(13)-C(15)	109.9(2)	N(2)-C(13)-C(16)	108.4(2)
C(14)-C(13)-C(15)	109.5(2)	C(16)-C(13)-C(14)	110.7(3)
C(16)-C(13)-C(15)	111.0(2)	C(13)-C(14)-H(14C)	109.5
C(13)-C(14)-H(14A)	109.5	C(13)-C(14)-H(14B)	109.5
H(14C)-C(14)-H(14A)	109.5	H(14C)-C(14)-H(14B)	109.5
H(14A)-C(14)-H(14B)	109.5	C(13)-C(15)-H(15A)	109.5
C(13)-C(15)-H(15B)	109.5	C(13)-C(15)-H(15C)	109.5
H(15A)-C(15)-H(15B)	109.5	H(15A)-C(15)-H(15C)	109.5
H(15B)-C(15)-H(15C)	109.5	C(13)-C(16)-H(16A)	109.5
C(13)-C(16)-H(16B)	109.5	C(13)-C(16)-H(16C)	109.5
H(16A)-C(16)-H(16B)	109.5	H(16A)-C(16)-H(16C)	109.5
H(16B)-C(16)-H(16C)	109.5	C(18)-C(17)-S(1)	120.2(3)
C(22)-C(17)-S(1)	121.1(3)	C(22)-C(17)-C(18)	118.7(3)
C(17)-C(18)-H(18)	119.6	C(17)-C(18)-C(19)	120.7(3)
C(19)-C(18)-H(18)	119.6	C(18)-C(19)-H(19)	120.0
C(20)-C(19)-C(18)	120.0(3)	C(20)-C(19)-H(19)	120.0
C(19)-C(20)-H(20)	120.0	C(19)-C(20)-C(21)	120.1(3)
C(21)-C(20)-H(20)	120.0	C(20)-C(21)-H(21)	119.9
C(20)-C(21)-C(22)	120.3(3)	C(22)-C(21)-H(21)	119.9
C(17)-C(22)-C(21)	120.3(3)	C(17)-C(22)-H(22)	119.9
C(21)-C(22)-H(22)	119.9	F(1)-C(23)-S(2)	111.6(2)
F(1)-C(23)-F(2)	107.8(2)	F(1)-C(23)-F(3)	107.9(2)
F(2)-C(23)-S(2)	110.8(2)	F(3)-C(23)-S(2)	110.90(19)
F(3)-C(23)-F(2)	107.7(2)	H(24A)-C(24)-H(24B)	109.5
H(24A)-C(24)-H(24C)	109.5	H(24B)-C(24)-H(24C)	109.5
C(25)-C(24)-H(24A)	109.5	C(25)-C(24)-H(24B)	109.5
C(25)-C(24)-H(24C)	109.5	C(24)-C(25)-H(25A)	108.2
C(24)-C(25)-H(25B)	108.2	C(24)-C(25)-C(26)	116.4(4)
H(25A)-C(25)-H(25B)	107.3	C(26)-C(25)-H(25A)	108.2
C(26)-C(25)-H(25B)	108.2	C(25)#1-C(26)-C(25)	112.8(6)
C(25)#1-C(26)-H(26A)	109.0	C(25)-C(26)-H(26A)	109.0
C(25)#1-C(26)-H(26B)	109.0	C(25)-C(26)-H(26B)	109.0

H(26A)-C(26)-H(26B)	107.8

Symmetry transformations used to generate equivalent atoms:

#1 -x+1,y,-z+3/2

7.4 Cambridge Structural Database (CSD) Searches

Database surveys were performed on March 30, 2024 to search for Bi–Bi, Bi–S and S–aryl distances as well as Bi–S–Bi and Bi–S–aryl angles for comparison to those in **2**, **3** and **4**. (WebCSD Version 1.9.32; https://www.ccdc.cam.ac.uk/structures/WebCSD/StructureSearch)

Figure S42: Parameters used for Bi–Bi distance CSD search.

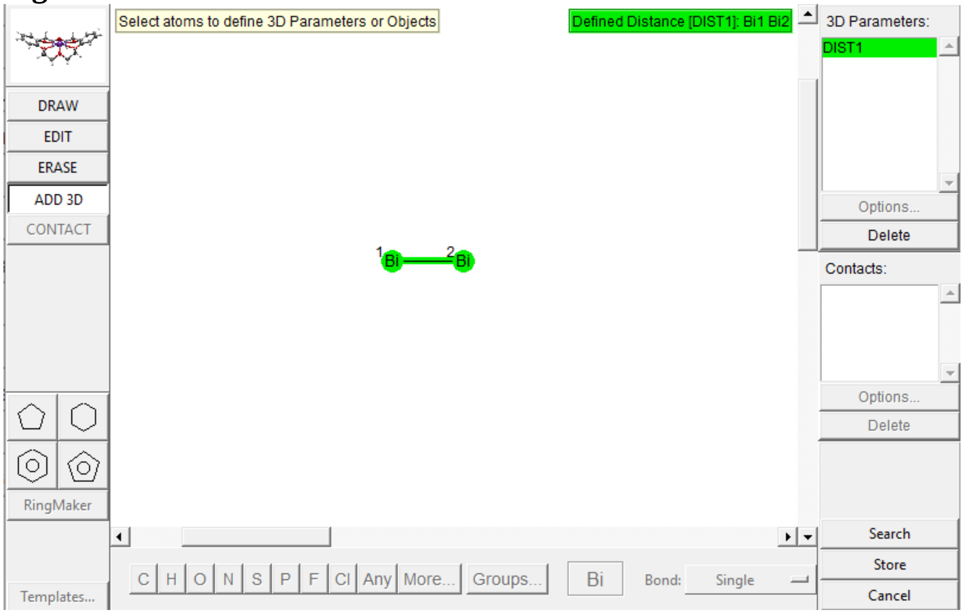

Figure S43: Parameters used for Bi–S distance and Bi–S–Bi angle CSD search.

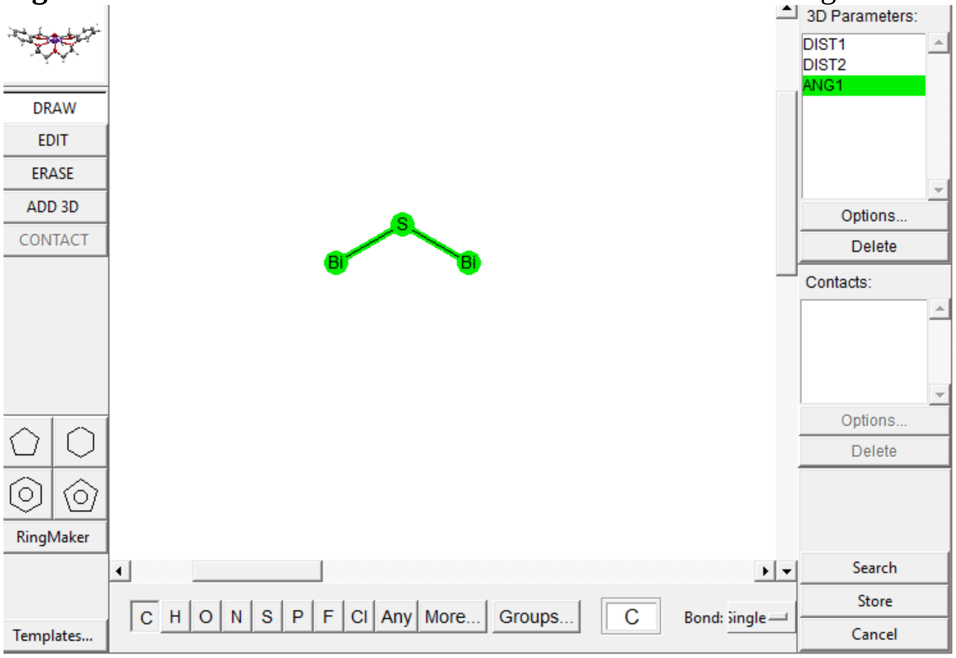

Figure S44: Parameters used for Bi–S, S–aryl distances and Bi–S–aryl angle CSD search.

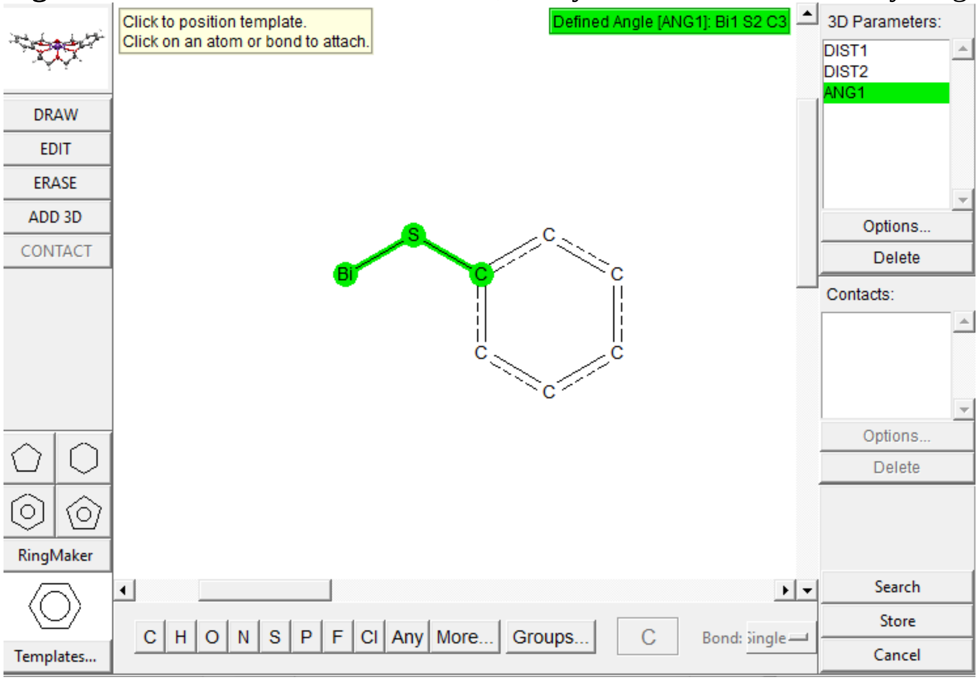

Table S11: Overview of search results for Bi–Bi distances.

Refcode	*Deposition #*	*Bi–Bi (Å)*	*R-factor (R)*	*Publication Year*
AZAHAM	1500596	3.021	2.48	2016
AZAHAM01	1446889	3.02	2.74	2016
AZAHAM02	1500597	3.023	2.65	2016
AZAHAM03	1500598	3.024	2.57	2016
AZAHAM04	1500599	3.03	3.11	2016
AZAHAM05	1500600	3.039	4.43	2016
BOZVER	1114845	2.99	5.55	1983
BOZVER01	1114846	2.983	3.8	1992
BOZVER02	1114847	2.987	3.4	1992
BOZVER10	1114848	2.99	5.55	1984
BOZVER11	1962843	2.987	3.64	2021
BOZVER11	1962843	2.996	3.64	2021
BUKXOV	742476	3.006	5.15	2010
DEVSUW	1985754	3.002	6.22	2022
DEVSUW	1985754	3.007	6.22	2022
DEVSUW	1985754	3.009	6.22	2022
DEVSUW	1985754	3.093	6.22	2022
DEVWAG	1985755	2.961	3.87	2022
DIMMUL	2243049	2.82	8.39	2023
DIMMUL	2243049	2.82	8.39	2023
DIMNAS	2243050	2.935	2.42	2023
DIMNUM	2243054	2.87	3.46	2023

DIMNUM	2243054	2.872	3.46	2023
ECEVEQ	1559486	2.87	5.14	2017
ECEWER	1559488	2.818	6.76	2017
ECEWER	1559488	2.819	6.76	2017
ECEWER	1559488	2.819	6.76	2017
EGENOU	689595	3.063	10.09	2008
EGEQEM	1149034	3.054	5.84	2002
ENIVUV	2012044	3.02	1.42	2021
EZOHUW	242847	2.943	2.36	2004
EZOHUW	242847	2.955	2.36	2004
EZOHUW01	242846	2.941	2.36	2004
EZOHUW01	242846	2.952	2.36	2004
EZOJEI	242849	2.963	2.3	2004
FARSOK	2125092	3.03	2.7	2022
FARSOK	2125092	3.032	2.7	2022
FARSUQ	2125093	3.00	5.66	2022
FARSUQ	2125093	3.003	5.66	2022
FIYXUH	247981	3.087	2.42	2005
GAVKIZ	808072	2.983	3.94	2011
GEPLOC	1166085	3.035	8.4	1983
GEPLOC01	1585346	3.048	3.95	2018
GUTLUE	1416634	2.796	5.61	2015
GUTMAL	1416635	2.861	3.45	2015
GUTMAL	1416635	2.863	3.45	2015
GUTMEP	1416636	2.887	2.96	2015
GUXGEO	1983873	2.803	3.74	2020
GUYMIW	195482	3.066	2.77	2003
HADVAN	2080779	2.927	4.33	2021
HADVAN	2080779	2.93	4.33	2021
HAHHUX	2080853	3.001	5.77	2021
HAWYAH	879222	2.961	2.43	2012
HAWYAH	879222	2.961	2.43	2012
HOBMOB	675732	3.023	3.93	2008
JAWWOX	2125603	2.897	5.5	2022
JOJHOG	704047	2.954	2.65	2008
JUGFAS	1190325	2.992	3.62	1992
KIKSAA	789992	3.02	1.63	2012
LOMHEC	999347	3.182	1.94	2014
MUDHAV	740995	3.065	1.78	2009
MUDHEZ	740996	3.055	1.84	2009
MUDHID	740997	3.071	2.29	2009
ODAMAH	164446	2.984	6.2	2001
ODAMEL	164447	2.983	2	2001

PERTIT	2203730	2.98	4.94	2023
PERTIT	2203730	2.98	4.94	2023
RAJEWEI	2077928	2.82	2.86	2021
RAJWAE	2077927	2.81	4.22	2021
RAJWAE	2077927	2.811	4.22	2021
RAJWEI	2077928	2.823	2.86	2021
RAJWIM	2077929	2.807	2.55	2021
RAJWIM	2077929	2.81	2.55	2021
REBYUT	284976	3.099	3.84	2006
REBZAA	284977	3.179	6.47	2006
REBZAA	284977	3.209	6.47	2006
RITMAM	2278294	2.98	3.38	2023
SABSUN	1990333	3.021	3.3	2020
SOBCUH	102011	2.947	3.49	1998
SORRAU	978281	3.013	6.24	2014
WESHAE	606899	2.942	7.39	2006
WEXFIO	1292751	3.045	6.2	1994
XACJEU	1937537	2.872	2.62	2020
XACJIY	1937538	2.865	3.34	2020
YOGLIR	981810	3.045	2.6	2014
YOGYIE	981809	3.037	2.32	2014

Figure S45: Plot of the Bi–Bi distance in **2** versus reported neutral Bi(II) dimers.

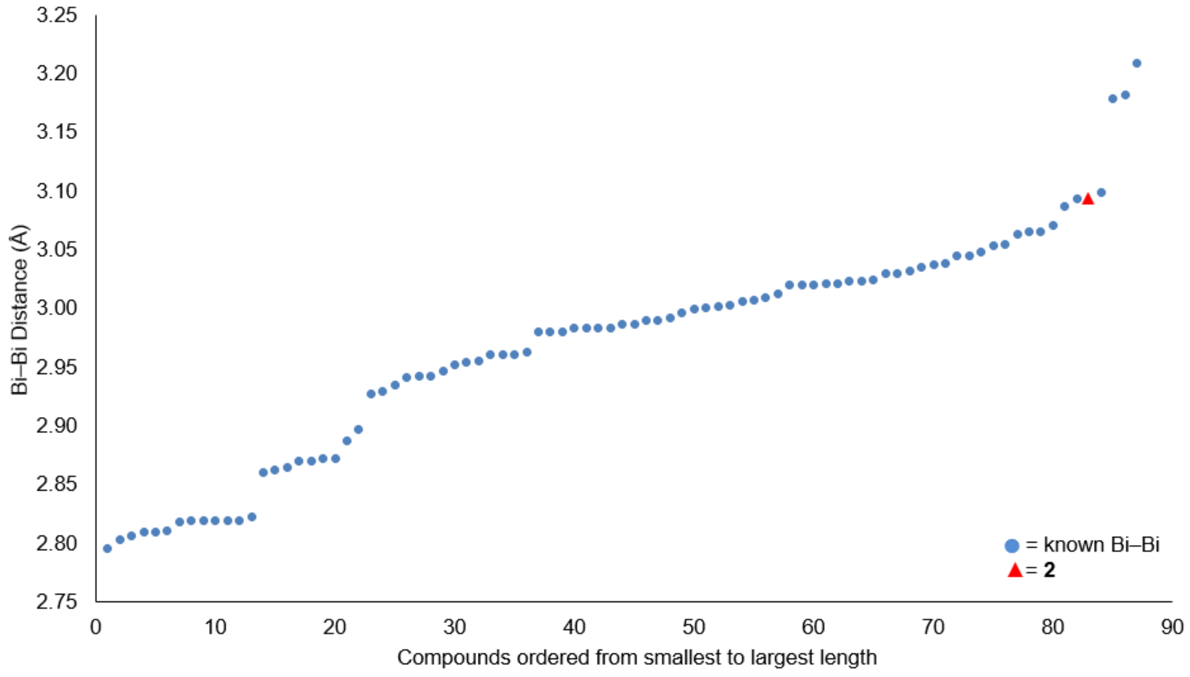

Table S12: Overview of search results for Bi–S distances and Bi–S–Bi angles.

Refcode	**Deposition #**	***Bi–S–Bi (°)***	***Bi–S (Å)***	***Bi–S (Å)***	***R-factor (R)***	***Publication Year (I)***
GEQSUT	1588407	110.968	2.535	2.528	2.87	2017
GEQTEE	1588409	96.274	2.562	2.527	3.34	2017
KIKSEE	789991	112.893	2.545	2.545	1.7	2012
KISKUT	650122	101.23	2.507	2.505	6.35	2007
LUQWUP	185871	92.483	2.572	2.557	2.15	2002
NOCDOZ	652892	94.991	2.577	2.578	2.82	2008
NOCGAO	658672	98.166	2.556	2.556	3.57	2008
RITNIV	2278301	104.8	2.52	2.53	3.11	2023
WAZWEB	824590	93.073	2.589	2.589	4.07	2012
WAZWIF	824591	88.763	2.601	2.601	2.26	2012
WAZWOL	824592	91.944	2.585	2.572	4.23	2012
YOZJON	713308	93.834	2.56	2.555	5.88	2009
YOZJON	713308	94.191	2.549	2.547	5.88	2009
YULYEJ	58776	98.734	2.52	2.545	8.26	1995

Figure S46: Plot of the Bi–S distances in **3** versus reported compounds containing two bismuth atoms bridged by a μ_2-sulfide ligand.

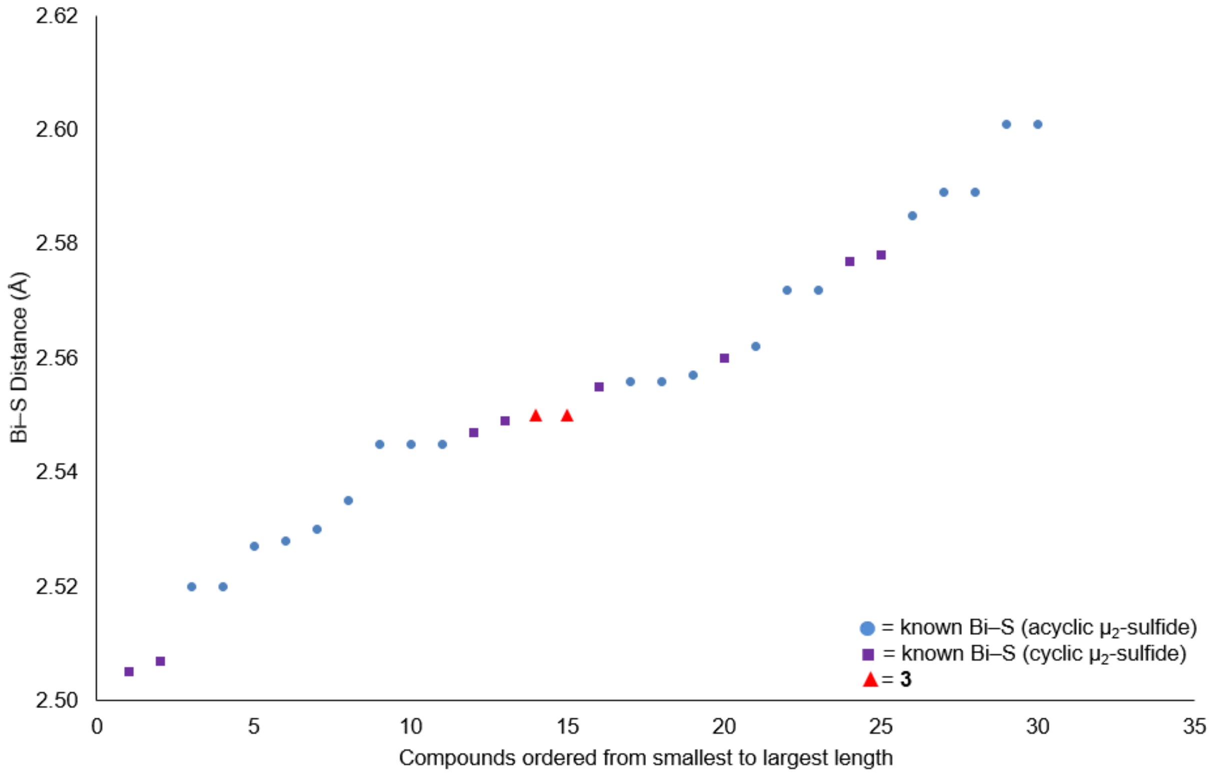

Figure S47: Plot of the Bi–S–Bi angle in **3** versus reported compounds containing two bismuth atoms bridged by a μ_2-sulfide ligand.

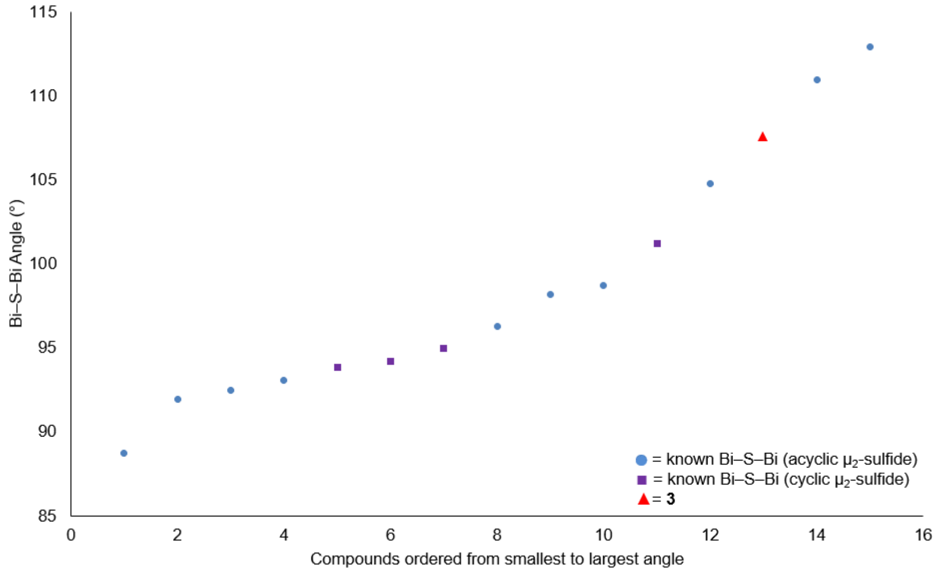

Table S13: Overview of search results for Bi–S and S–aryl distances and Bi–S–aryl angles.

Refcode	***Deposition #***	***Bi–S–aryl (°)***	***Bi–S (Å)***	***S–aryl (Å)***	***R-factor (R)***	***Publication Year***
ABONIP	234081	103.739	2.634	1.763	2.71	2004
DIFMIO	1140501	103.126	2.59	1.74	3.1	1985
DIFMIO	1140501	103.453	2.532	1.765	3.1	1985
KIMMAW	905886	106.09	2.615	1.773	3.22	2013
OJAJER	1948150	95.063	2.659	1.773	1.24	2020
OKIJUN	207456	98.307	2.602	1.783	4.43	2003
PAFQIY	836302	107.776	2.588	1.784	2.46	2012
PAFQOE	836303	98.247	2.544	1.784	2.7	2012
RITMIU	2278296	98.7	2.54	1.79	4.2	2023
XOMHUF	1884685	97.574	2.615	1.777	2.78	2019
ZAZYUU	1309990	104.682	2.661	1.751	2.61	1995
ZAZZAB	1309991	105.465	2.664	1.764	4.1	1995

Figure S48: Plot of the Bi–S distance in **4** versus reported compounds containing bismuth coordinated by a mono thioarylate ligand.

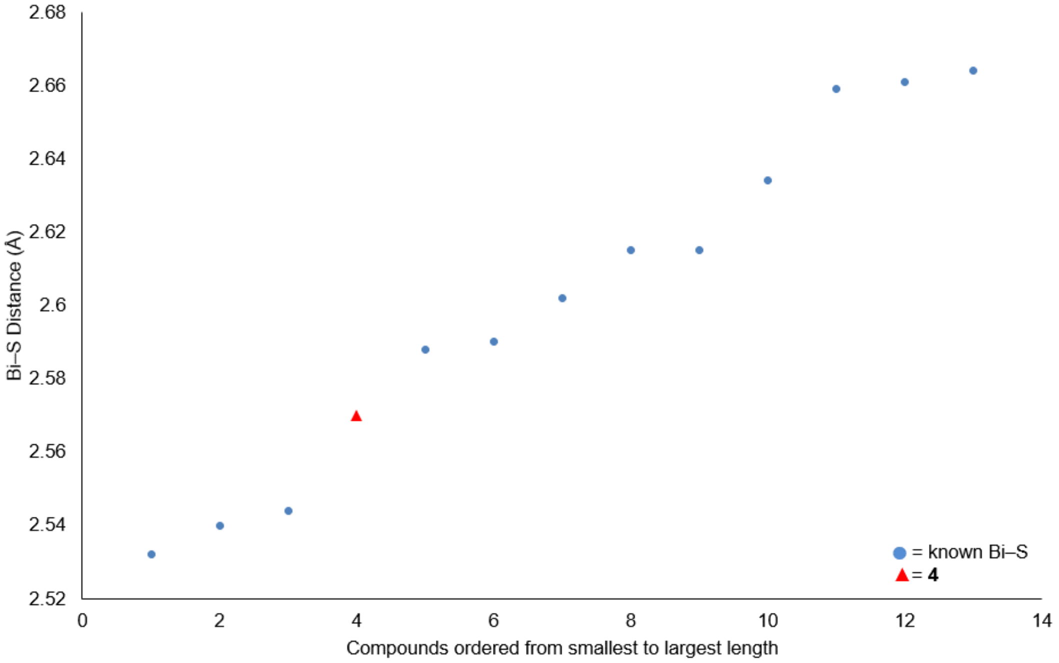

Figure S49: Plot of the S–aryl distance (S(1)–C(17)) in **4** versus reported compounds containing bismuth coordinated by a mono thioarylate ligand.

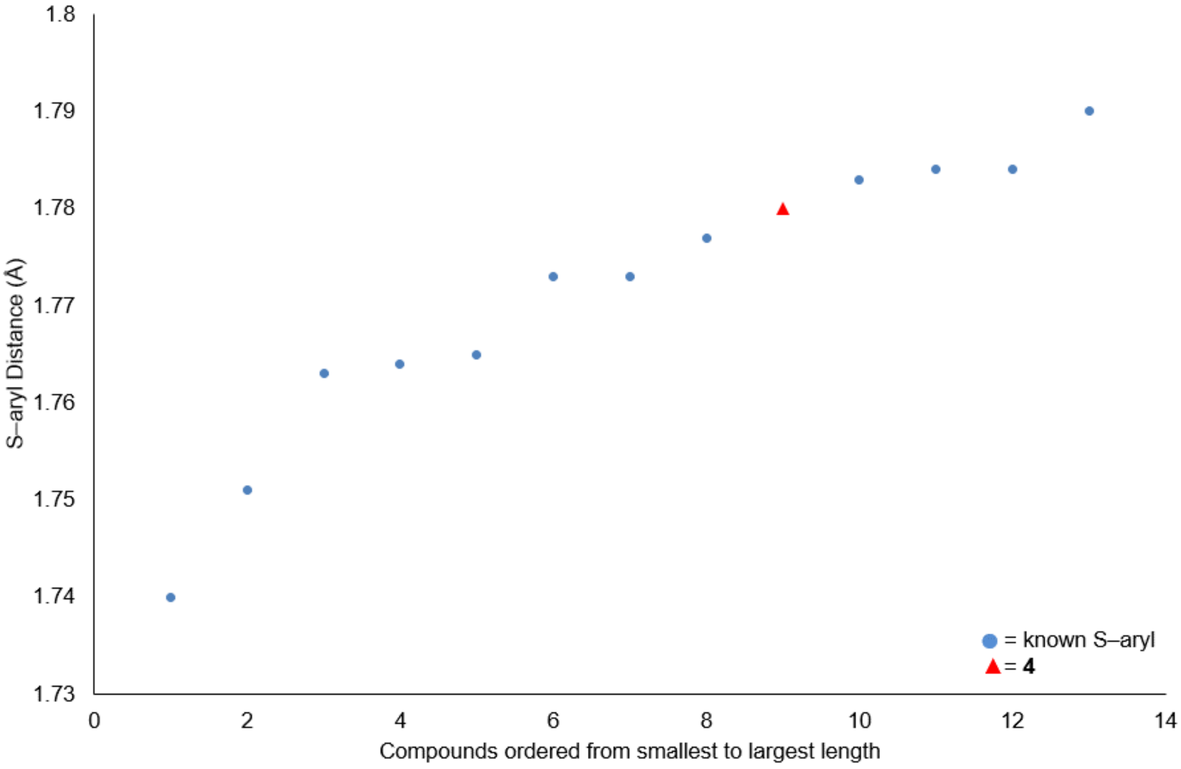

Figure S50: Plot of the Bi–S–aryl angle (Bi(1)–S(1)–C(17)) in **4** versus reported compounds containing bismuth coordinated by a mono thioarylate ligand.

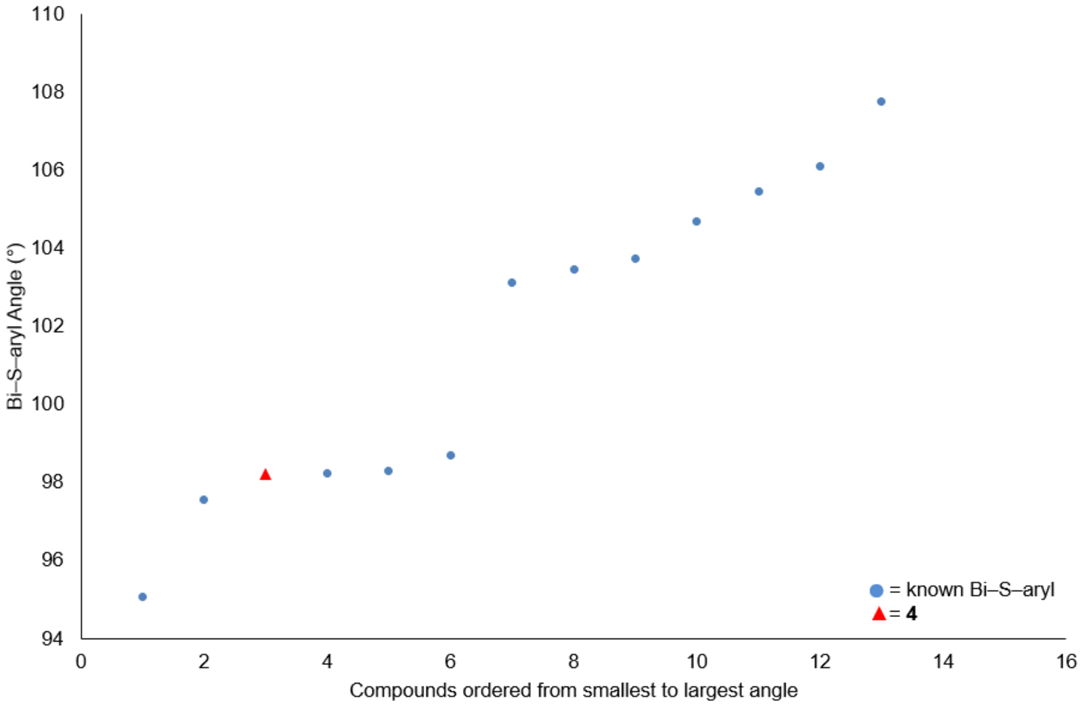

7.5 TEM EDX

Sample Preparation: For these experiments, a TEM vacuum transfer holder (Gatan Model 647) was transferred into a glove box. Inside the glove box, a tiny amount of crystalline sample of either **2** or **3** was placed on lacey carbon film supported by a copper TEM grid and the sample holder was sealed under inert conditions. After extracting from glove box, the device was inserted into the high vacuum column of a 200 kV STEM (Hitachi HD-2700 C_S) equipped with an Octane (T Ultra W 200mm^2) EDAX detector. In order to have a statistically meaningful result, three different positions (A, B and C) were analyzed.

7.5.1 TEM EDX analysis of compound **2**

Figure S51: TEM images of the crystalline sample of **2**.

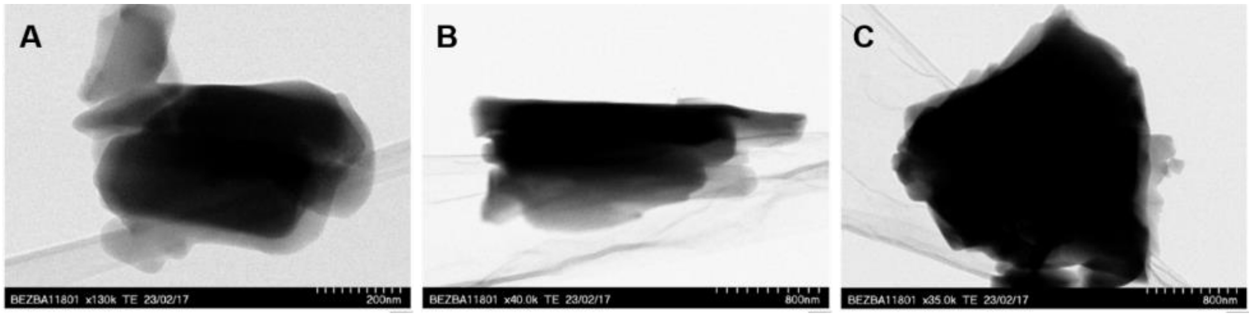

Figure S52: Overlay of EDX spectra (position A, B, C) of **2** with automated element search and a partial view of all three spectra from each measured spot, showing the absence of characteristic Cl lines and confirming the identity of the Bi atom.

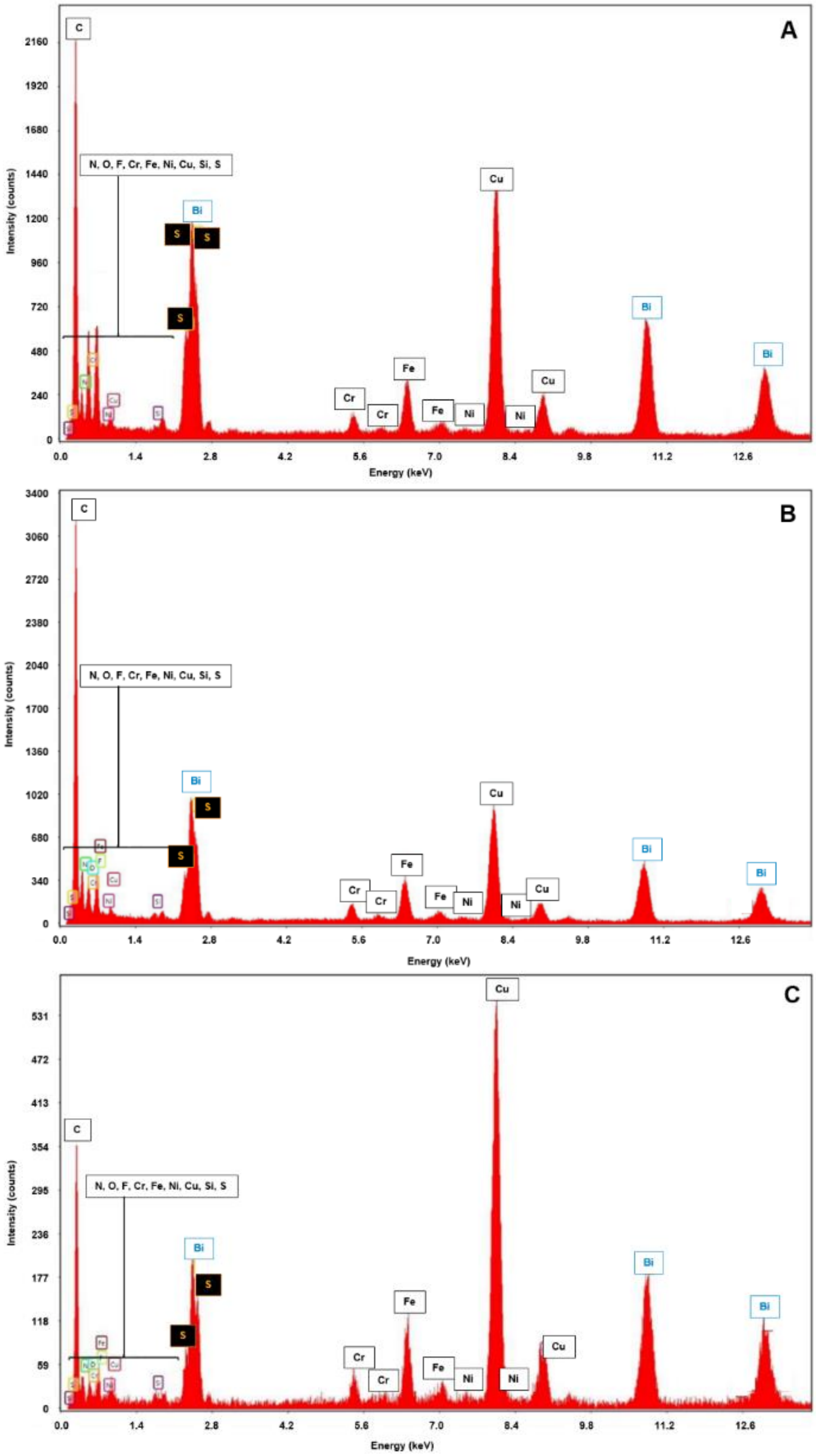

7.5.2 TEM EDX analysis of Compound **3**

Refinement of the crystal structure of **3** revealed a high residual electron density between the two Bi atoms. However, the refinement showed that both S and Cl could be properly refined at this position. Neither the distances nor the angles could provide reliable information about the nature of the bridging atom. The compounds were therefore analyzed by TEM-EDX to confirm that it was a bridging S atom and to rule out the presence of Cl. For this purpose, three different crystallites were analyzed and the X-ray spectra obtained were checked for the presence of characteristic Cl lines. In all cases no Cl line could be found, confirming the structure containing a bridging S atom.

Figure S53: TEM images of the crystalline sample of **3**.

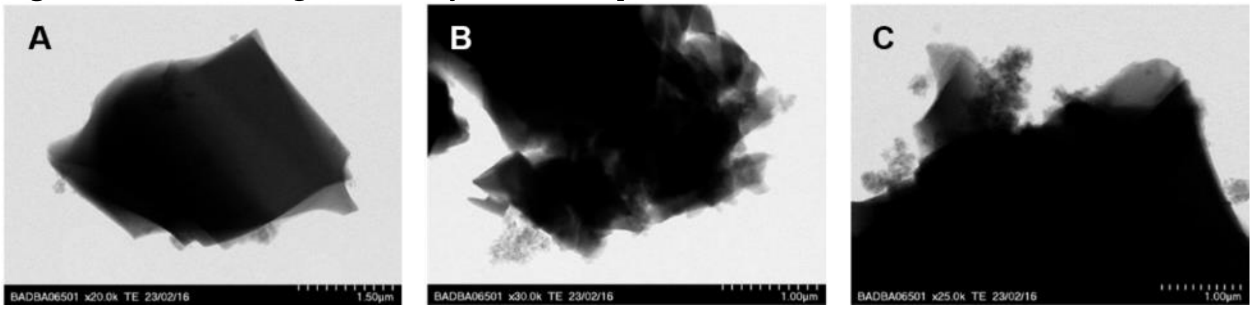

Figure S54: Overlay of EDX spectra (position A, B, C) of **3** with automated element search and a partial view of all three spectra showing the absence of characteristic Cl lines.

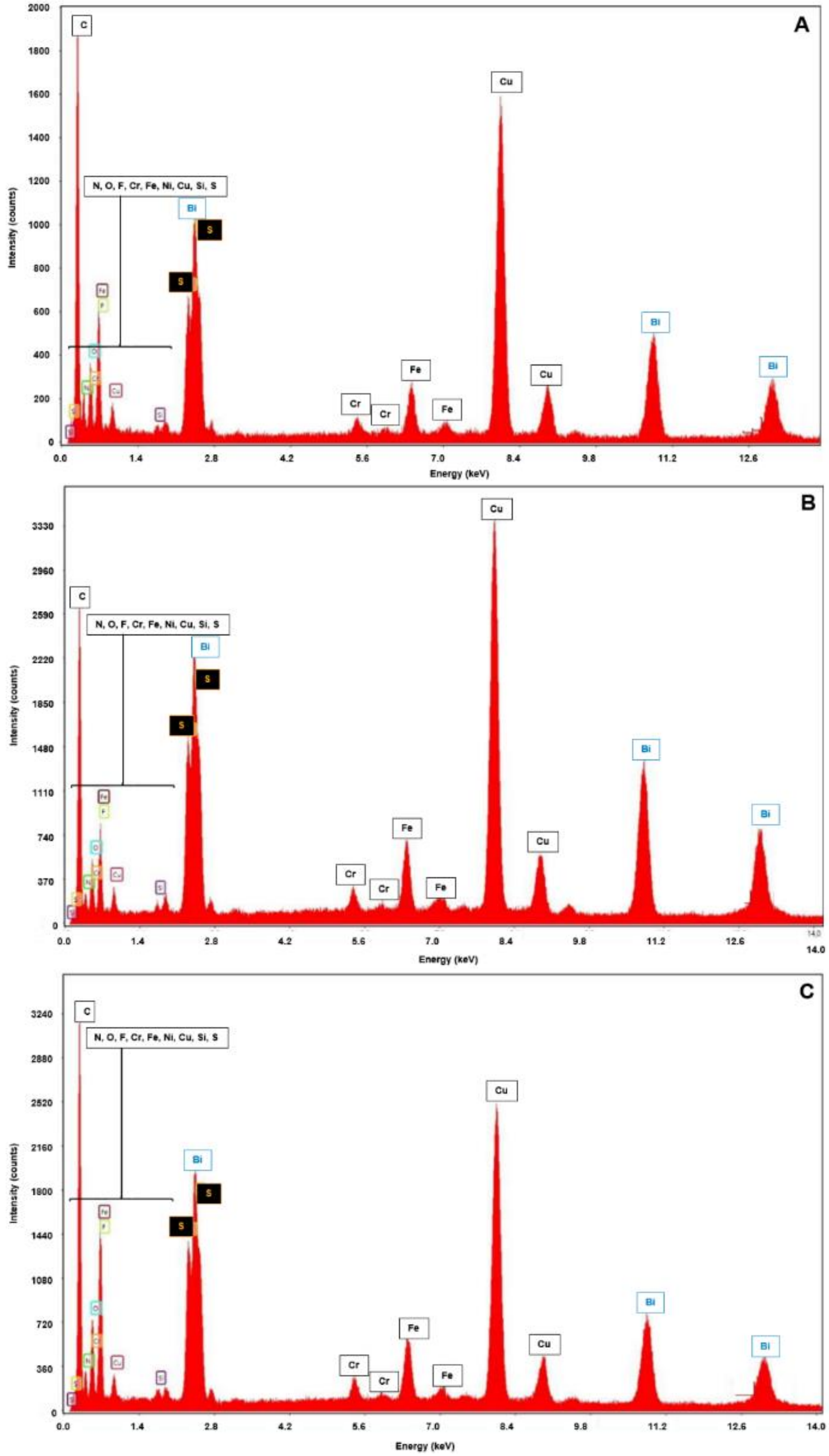

8. NMR Spectra

Figure S55: ^{1}H NMR spectrum (600 MHz, CD_3CN) of **2** isolated from the SF_6 reaction mixture. Inset zoom of the aromatic region and showing a 3% impurity of **3** (*).

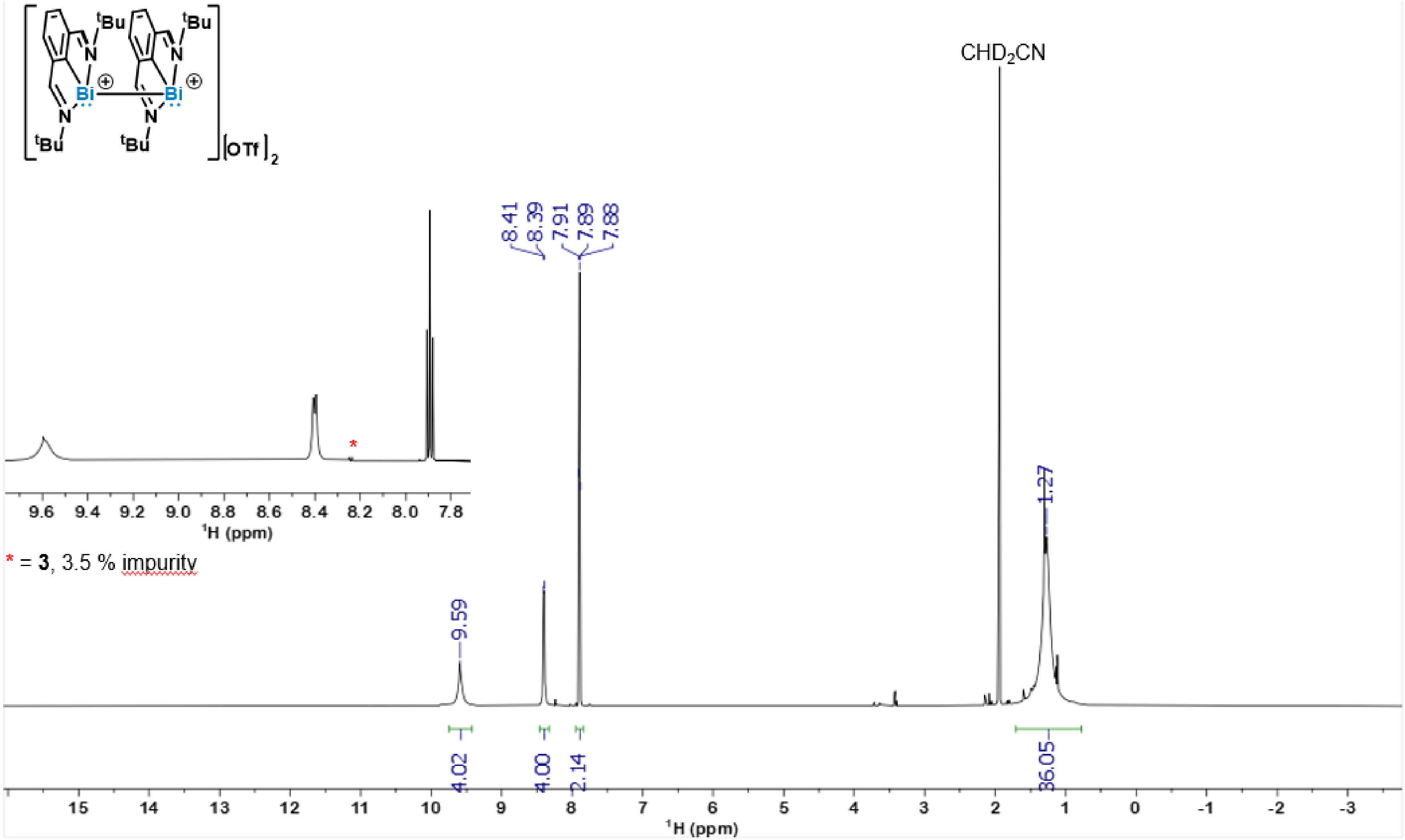

Figure S56: $^{13}C\{^{1}H\}$ NMR spectrum (150.9 MHz) of **2** in CD_3CN.

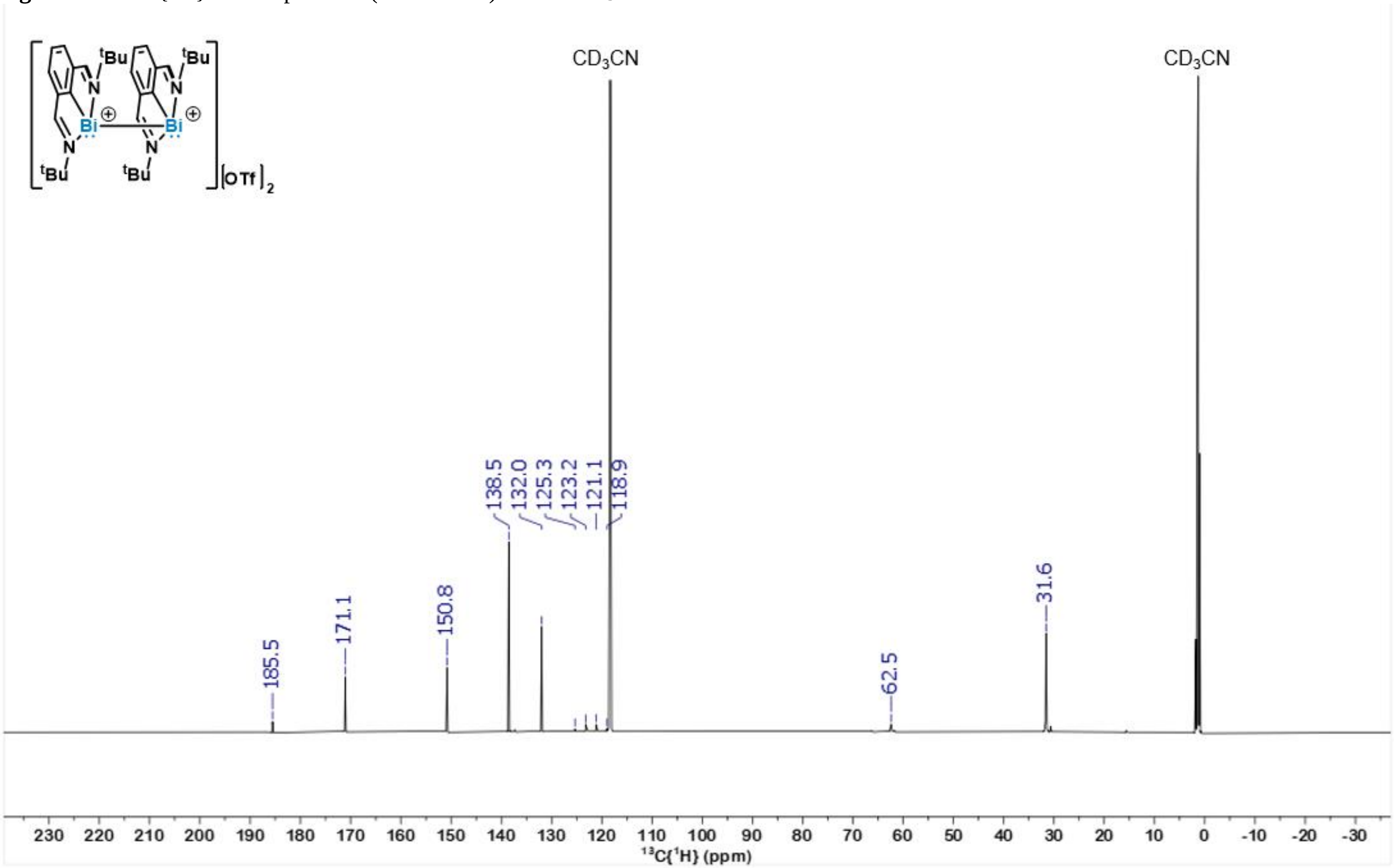

Figure S57: ^{19}F NMR spectrum (564.8 MHz) of **2** in CD_3CN.

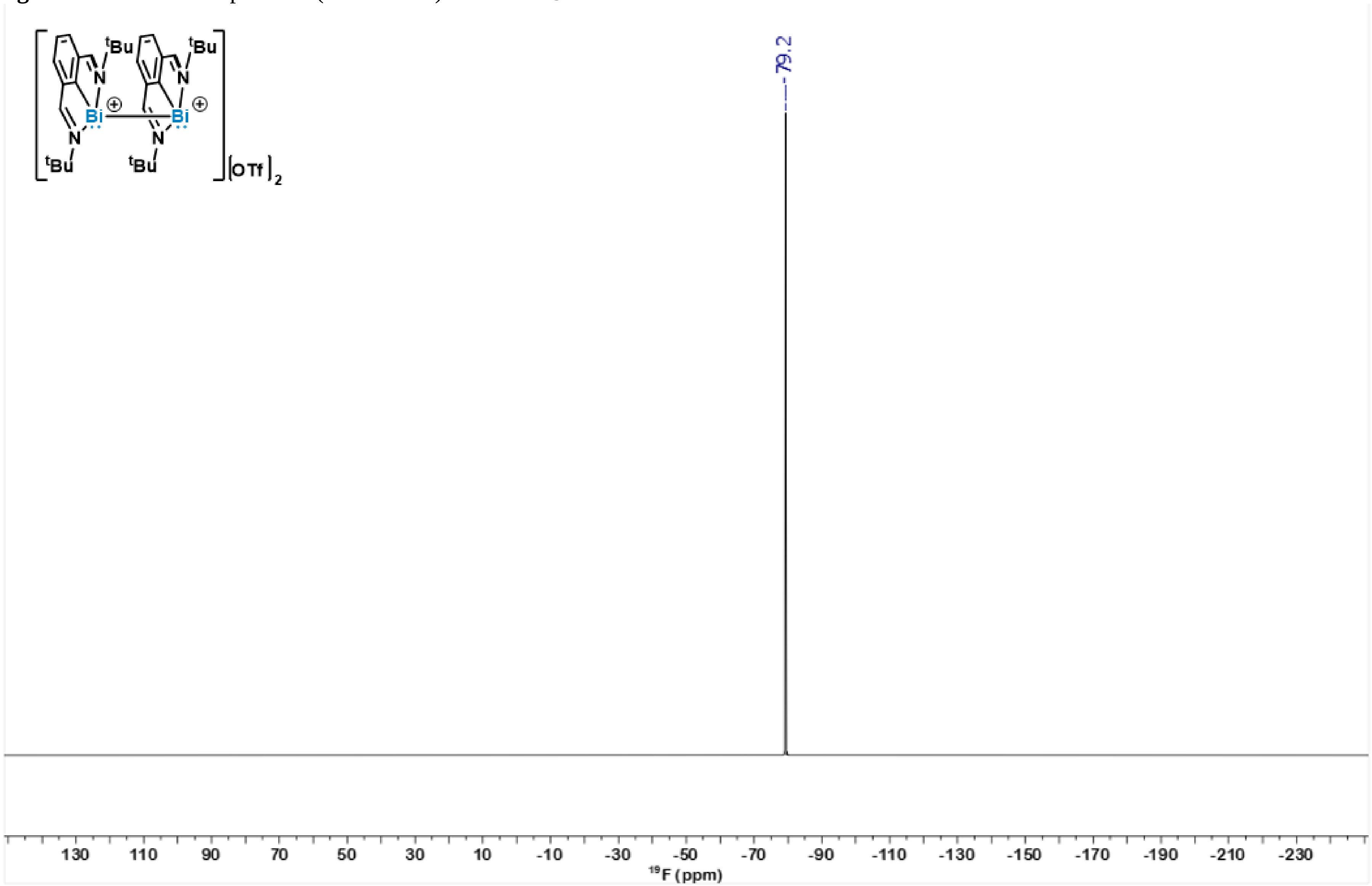

Figure S58: ^1H NMR spectrum (600 MHz) of **3** in CD_3CN with inset zoom of the aromatic region.

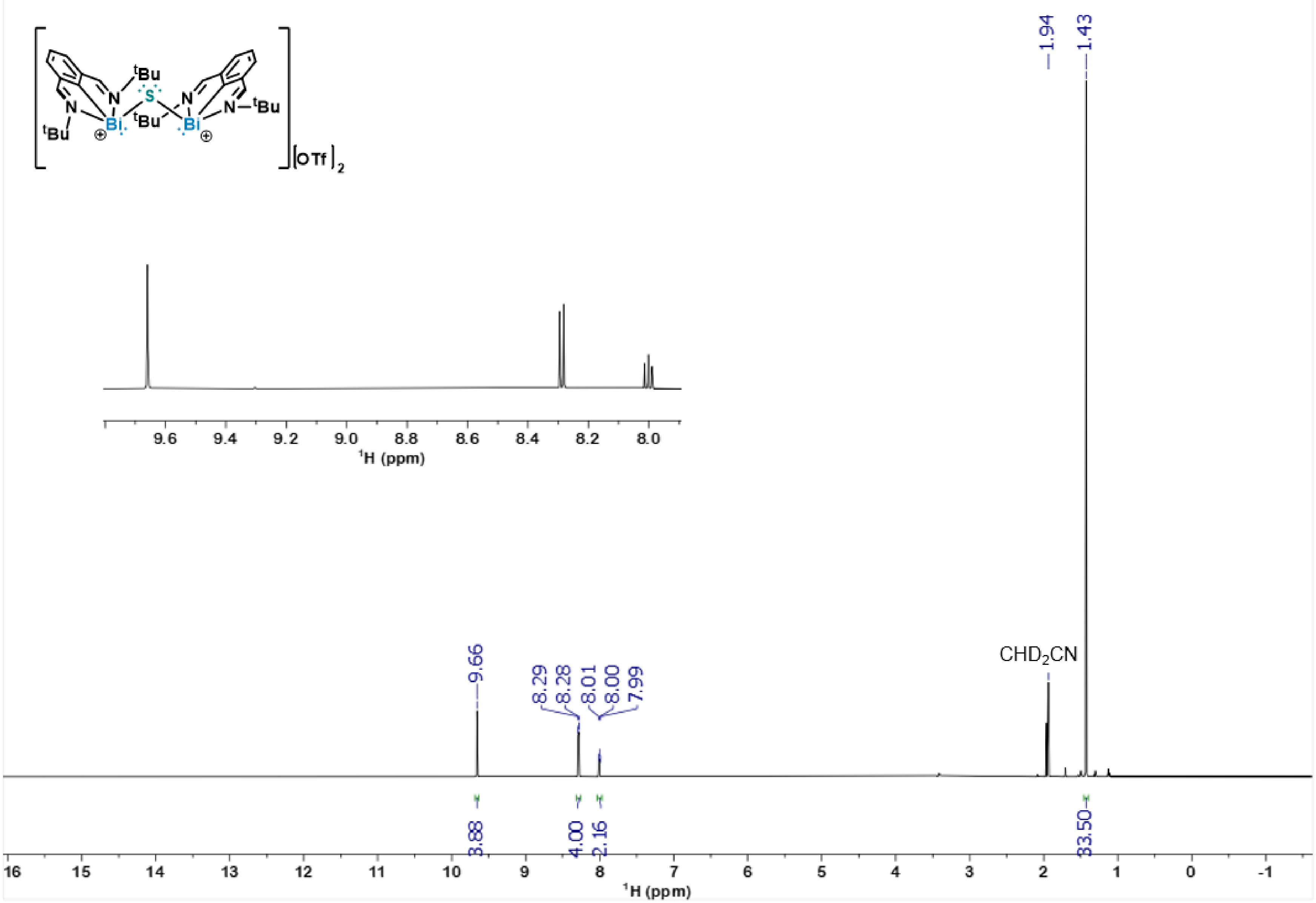

Figure S59: $^{13}C\{^1H\}$ NMR spectrum (150.9 MHz) of **3** in CD_3CN.

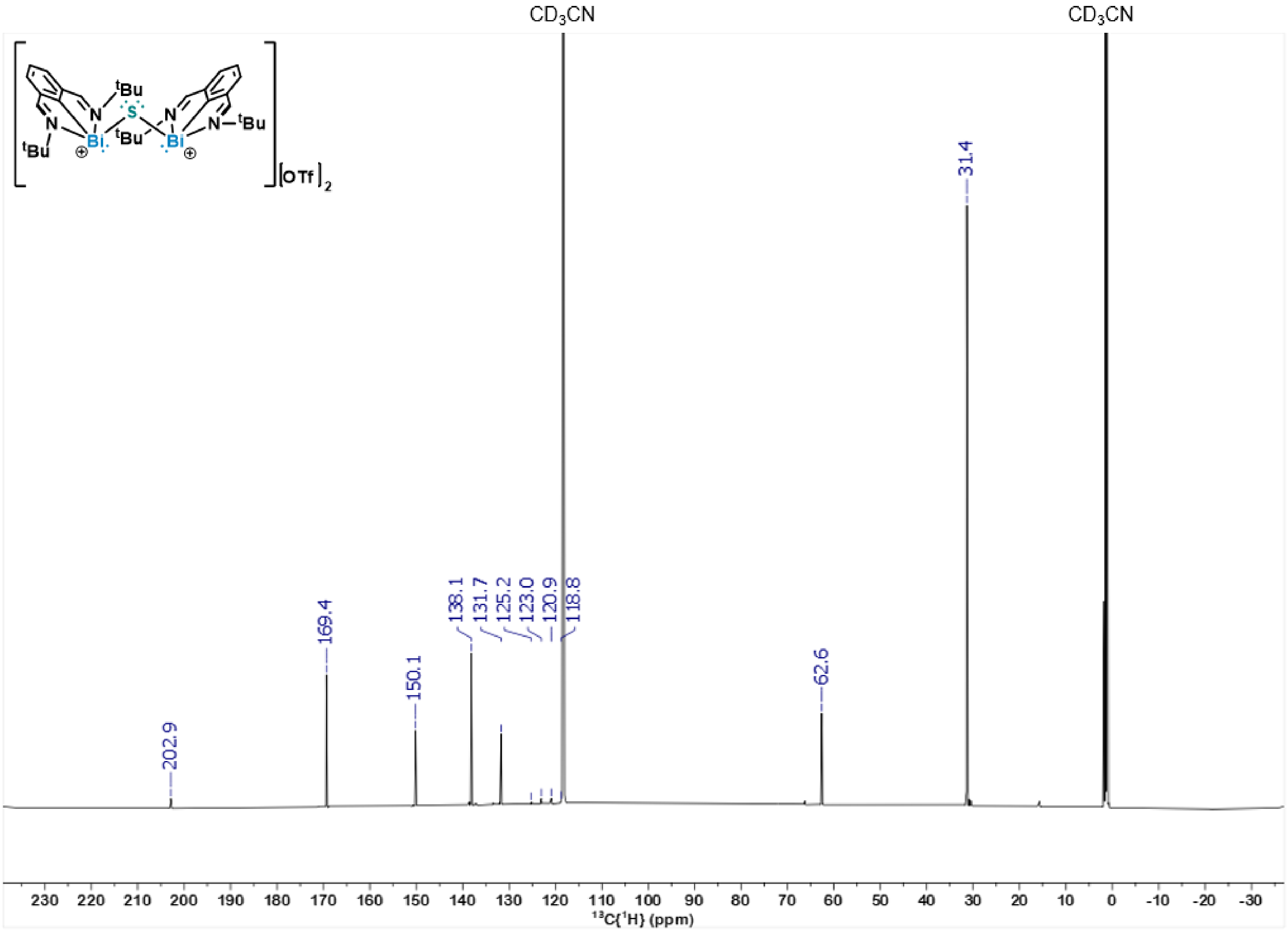

Figure S60: ^{19}F NMR spectrum (564.8 MHz) of **3** in CD_3CN.

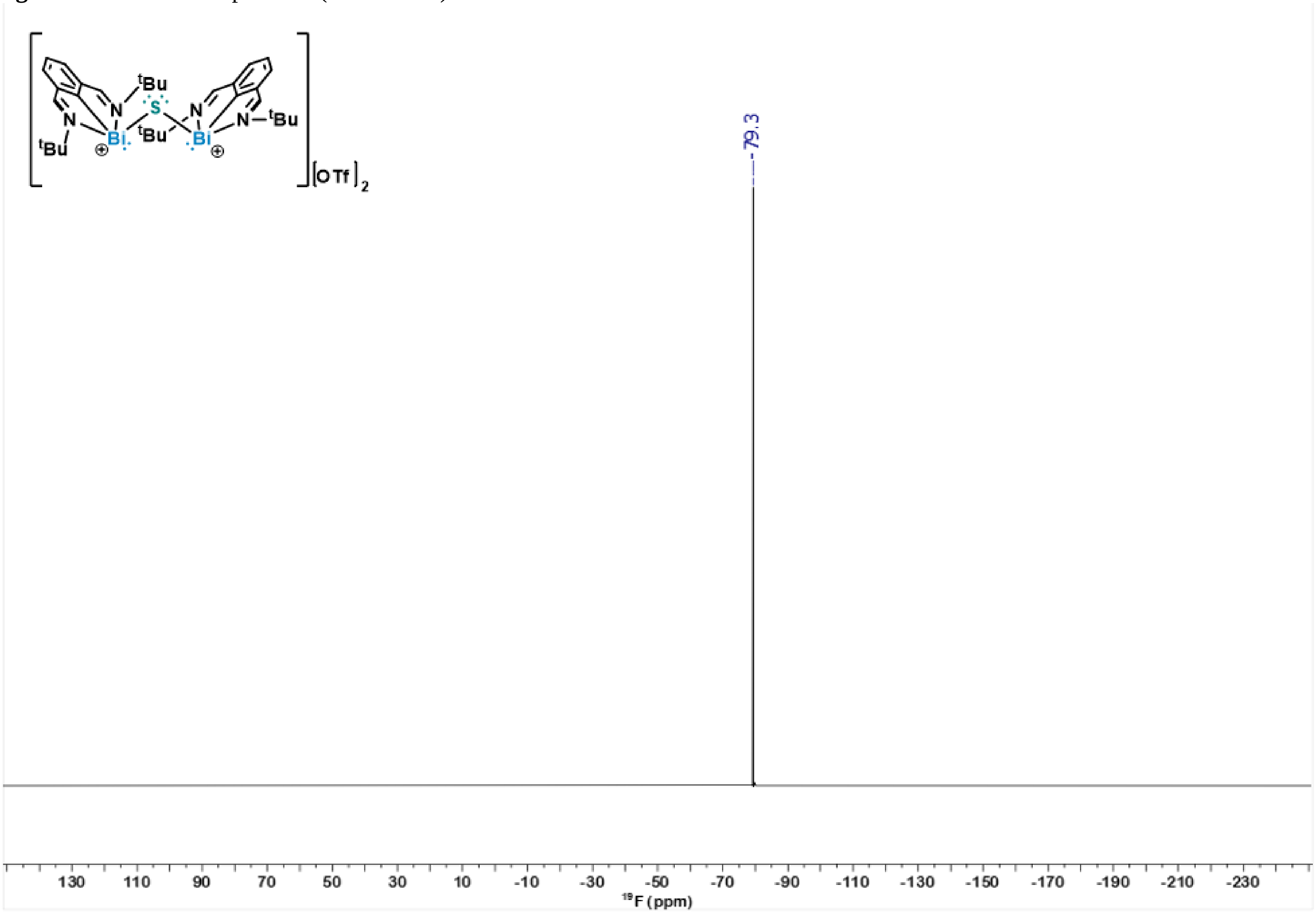

Figure S61: ^{1}H NMR spectrum (600 MHz) of **4** in CD_3CN.

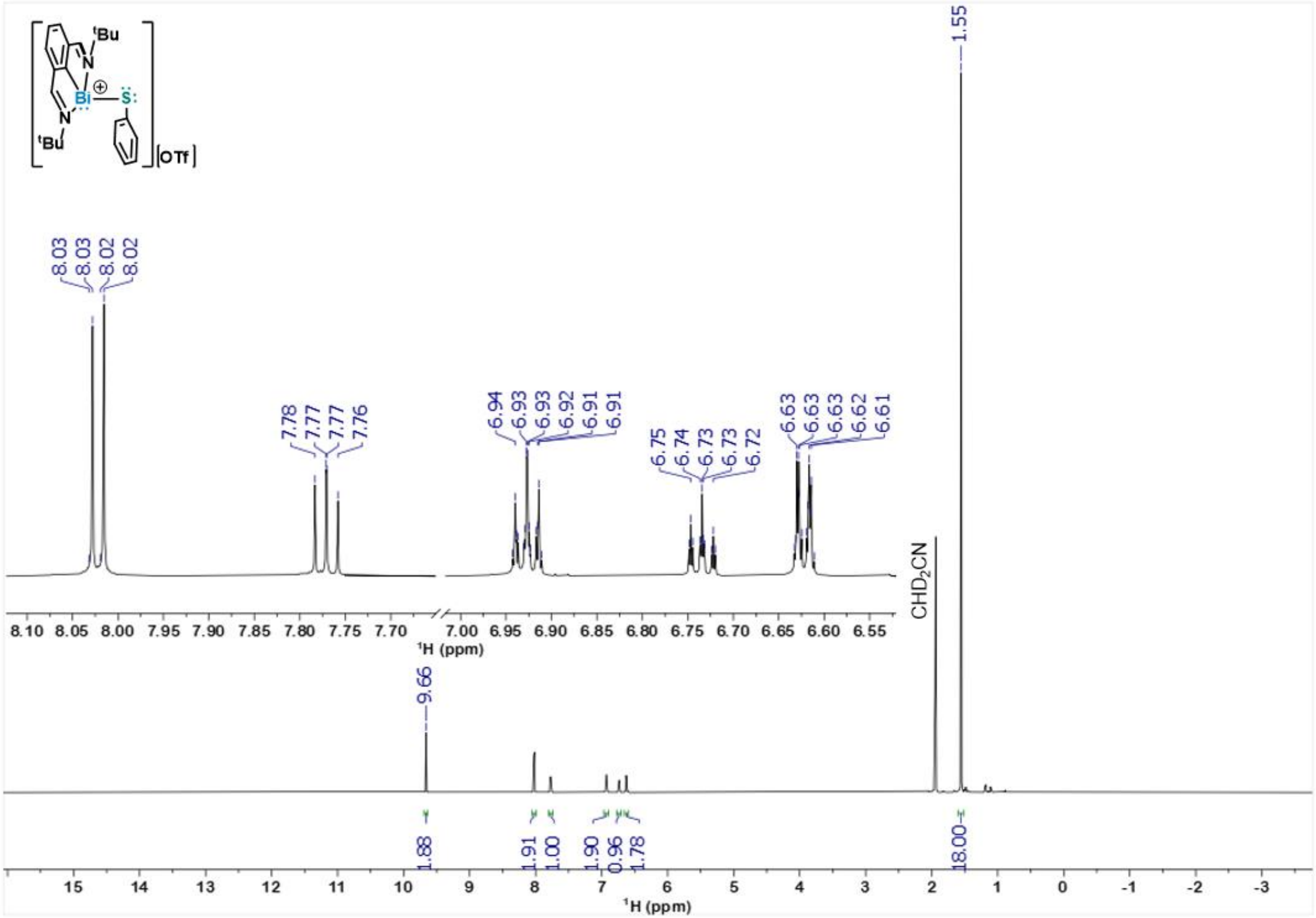

Figure S62: $^{13}C\{^{1}H\}$ NMR spectrum (150.9 MHz) of **4** in CD_3CN.

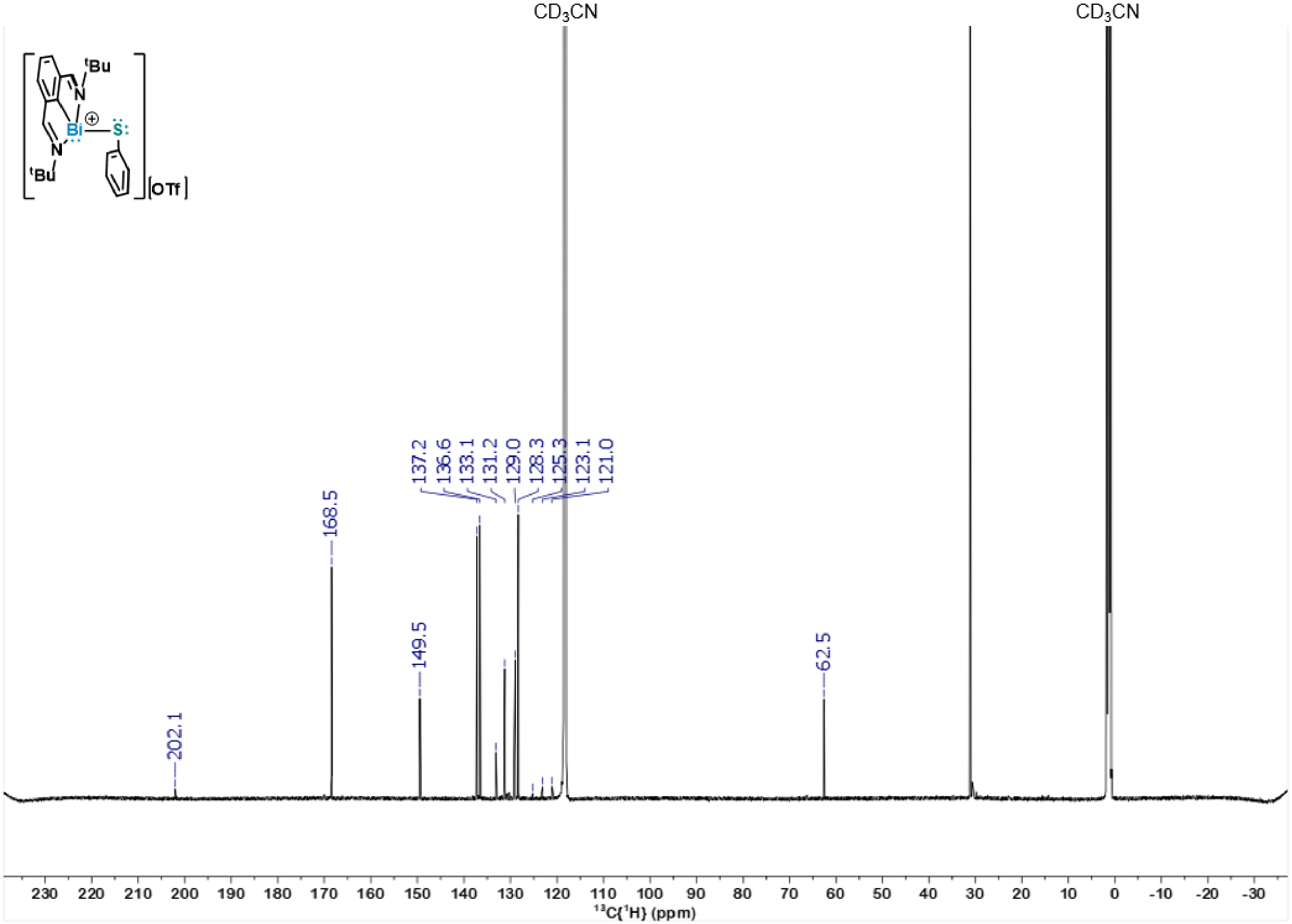

Figure S63: ^{19}F NMR spectrum (564.8 MHz) of **4** in CD_3CN.

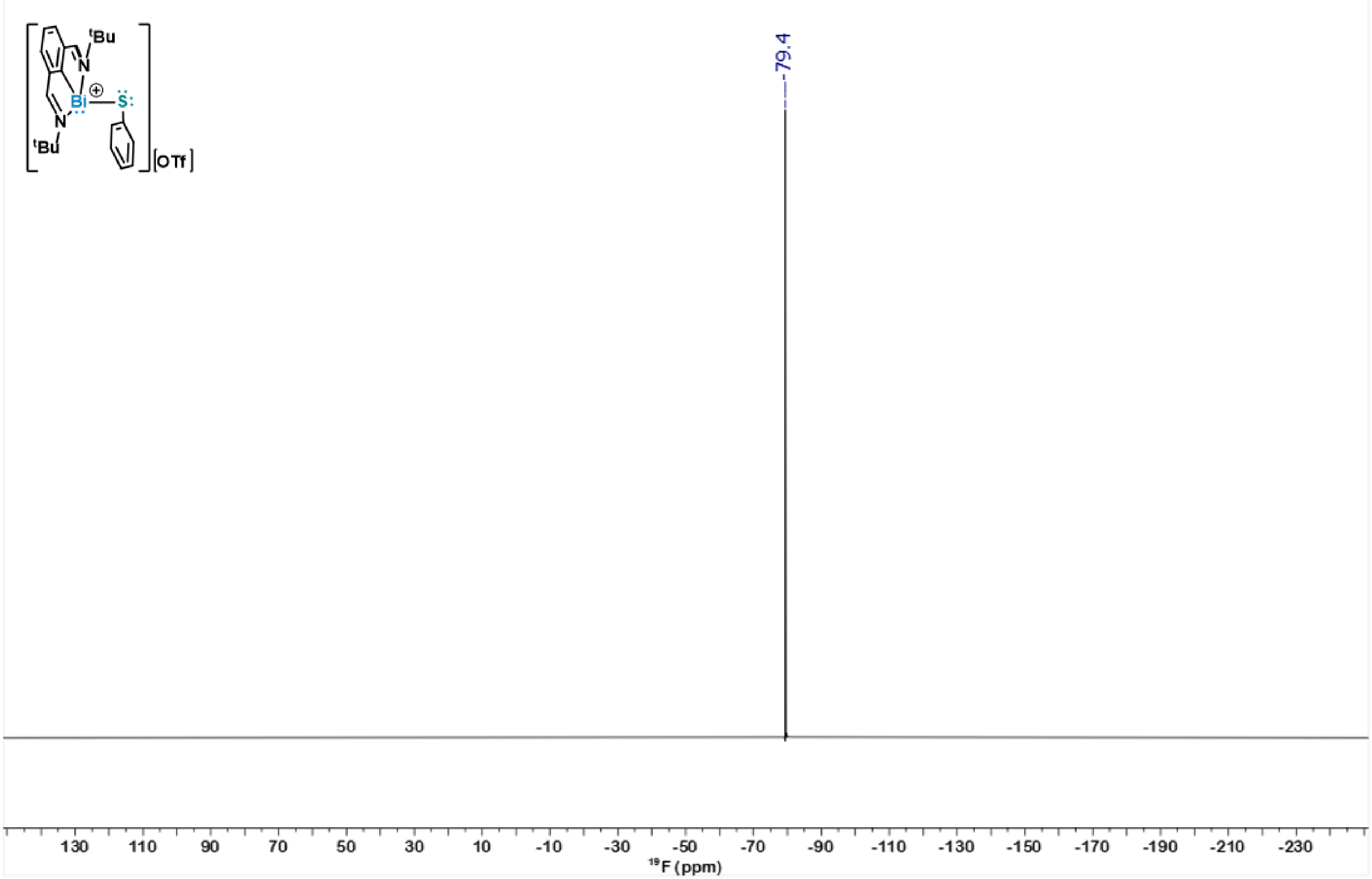

9. Mass Spectra

Figure S64: Positive ion ESI mass spectrum for **3**.

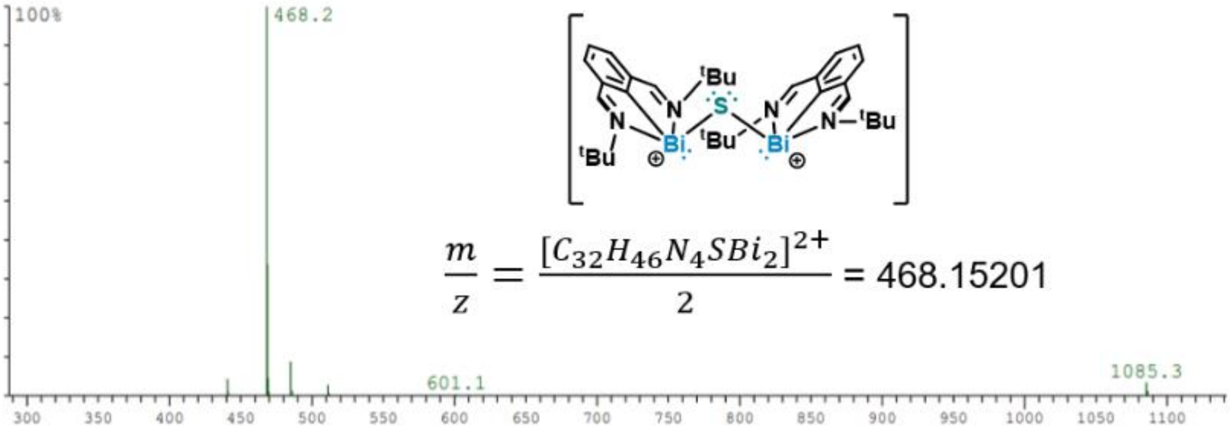

Figure S65: Negative ion ESI mass spectrum for **3**. $[CO_3F_3S]^-$ ($[M]^-$) m/z 149.0.

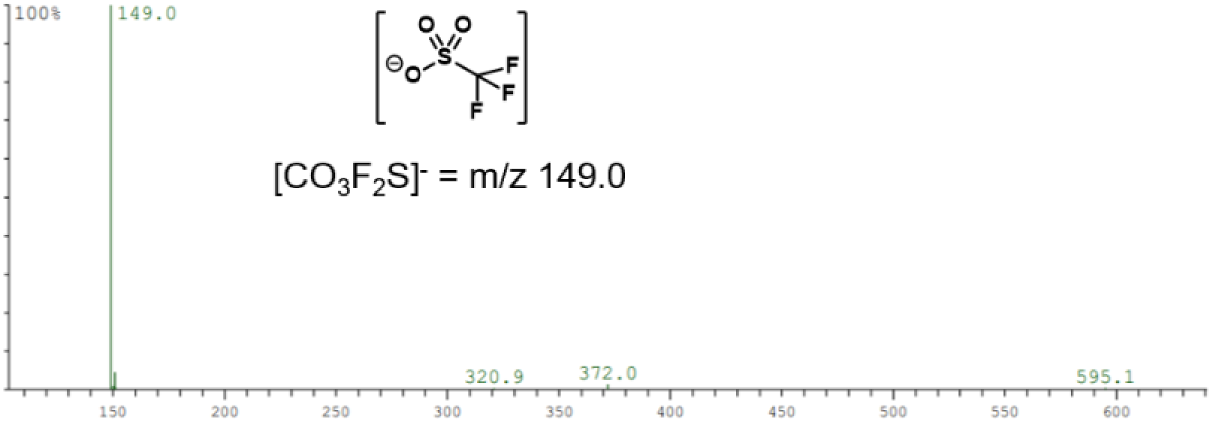

Figure S66: Positive ion ESI mass spectrum for **4**.

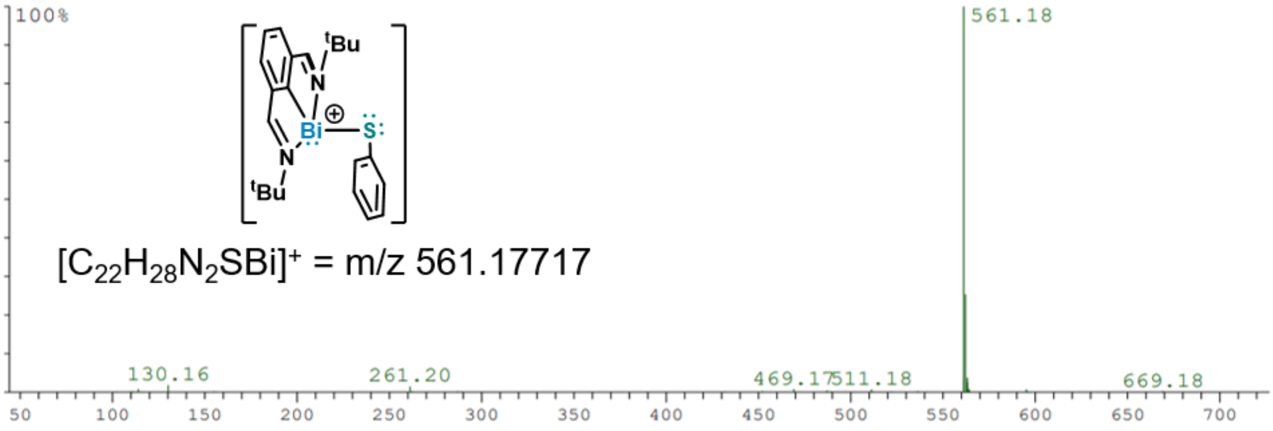

Figure S67: Negative ion ESI mass spectrum for **4**.

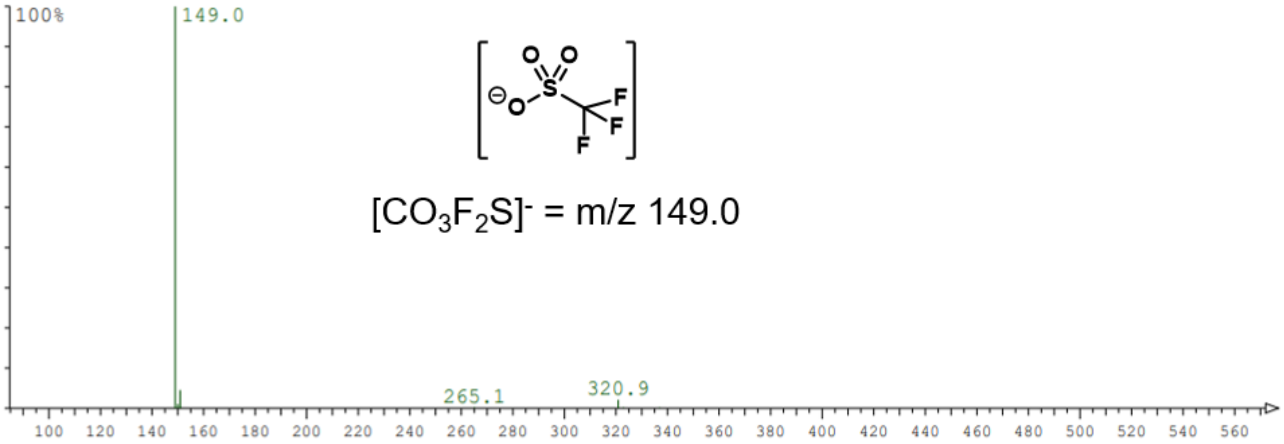

10. References

(1) Wang, F.; Planas, O.; Cornella, J. Bi(I)-Catalyzed Transfer-Hydrogenation with Ammonia-Borane. *J. Am. Chem. Soc.* **2019**, *141*, 4235–4240.

(2) Vránová, I.; Jambor, R.; Růžička, A.; Jirásko, R.; Dostál, L. Reactivity of *N,C,N*-Chelated Antimony(III) and Bismuth(III) Chlorides with Lithium Reagents: Addition vs Substitution. *Organometallics* **2015**, *34*, 534–541.

(3) Pang, Y.; Leutzsch, M.; Nöthling, N.; Cornella, J. Catalytic Activation of N_2O at a Low-Valent Bismuth Redox Platform. *J. Am. Chem. Soc.* **2020**, *142*, 19473–19479.

(4) Moon, H. W.; Wang, F.; Bhattacharyya, K.; Planas, O.; Leutzsch, M.; Nöthling, N.; Auer, A. A.; Cornella, J. Mechanistic Studies on the Bismuth-Catalyzed Transfer Hydrogenation of Azoarenes. *Angew. Chem. Int. Ed.* **2023**.

(5) Bailey, B. C.; Basuli, F.; Huffman, J. C.; Mindiola, D. J. Terminal Titanium(IV) (Trimethylsilyl)Imides Prepared by Oxidatively Induced Trimethylsilyl Abstraction. *Organometallics* **2006**, *25*, 2725–2728.

(6) Hopkins, P. B.; Fuchs, P. L. Chlorosulfenylation-Dehydrochlorination Reactions. New and Improved Methodology for the Synthesis of Unsaturated Aryl Sulfides and Aryl Sulfones. *J. Org. Chem.* **1978**, *43*, 1208-1217.

(7) Schwamm, R. J.; Lein, M.; Coles, M. P.; Fitchett, C. M. Bismuth(III) Complex of the $[S_4]^{\bullet-}$ Radical Anion: Dimer Formation via Pancake Bonds. *J. Am. Chem. Soc.* **2017**, *139*, 16490–16493.

(8) Sun, S. Benzenesulfenyl Triflate. *Encyclopedia of Reagents for Organic Synthesis*; John Wiley & Sons, Ltd, **2004**.

(9) Mato, M.; Spinnato, D.; Leutzsch, M.; Moon, H. W.; Reijerse, E. J.; Cornella, J. Bismuth Radical Catalysis in the Activation and Coupling of Redox-Active Electrophiles. *Nat. Chem.* **2023**, 1138-1145.

(10) Sun, S. G; Chen S. P.; Li, N. H.; Lu, G. Q.; Chen, B. Z.; Xu, F. C. Chemical States of Bismuth and Sulfur Adatoms on the Polycrystalline Pt Electrode Surface towards HCOOH Oxidation Combined Studies of Cyclic Voltammetry, in Situ FTIRS and XPS on the Origin of Electrocatalytic Activity of Adatoms. *Colloid. Surface. A* **1998**, *134*, 207–220.

(11) Nascimento, V. B.; de Carvalho, V. E.; Raniago, R.; Soares, E. A.; Ladeira, L. O.; Pfannes, H. D. XPS and EELS Study of the Bismuth Selenide. *J. Electron Spectrosc.* **1999**, *104*, 99–107.

(12) Fantauzzi, M.; Elsener, B.; Atzei D.; Rigoldi, A.; Rossi, A. Exploiting XPS for the Identification of Sulfides and Polysulfides. *RSC Adv.* **2015**, *5*, 75953–75963.

(13) Kratzert, D.; Holstein J. J.; Krossing, I. DSR: enhanced modelling and refinement of disordered structures with SHELXL. *J. Appl. Cryst*. **2015**, *48*, 933-938.

(14) Kratzert, D.; Krossing, I. Recent improvements in DSR. *J. Appl. Cryst*. **2018**, *51*, 928-934.
